# Prevalence, hormonal correlates, severity, and neural basis of neurocognitive impairment in patients with hypothyroidism: Systematic review and meta‐analyses

**DOI:** 10.1002/alz.70924

**Published:** 2025-11-26

**Authors:** Daniel Pankowski, Kinga Wytrychiewicz‐Pankowska

**Affiliations:** ^1^ Vizja University Warsaw Poland; ^2^ Faculty of Psychology University of Warsaw Warsaw Poland

**Keywords:** cognitive impairment, hypothyroidism, neuroimaging, prevalence, TSH

## Abstract

The aims of this review were to assess the associations between neuropsychological test results and thyroid hormone levels, as well as the imaging abnormalities in the brain associated with hypothyroidism. For this systematic review and meta‐analysis, 85 prior studies met the inclusion criteria. Among these studies, the prevalence of neurocognitive impairment assessed with the Mini‐Mental State Examination (MMSE) in hypothyroidism was 0.29 [95% CI:0.26−0.33]. The pooled correlation strength between thyroid‐stimulating hormone and the MMSE was *r* = −0.46 [95% CI:−0.69 to −0.15]. Patients with hypothyroidism performed lower than controls on the MMSE (standardized mean difference (SMD) = −1.134, [95% CI:−2.020 to −0.248]), Wechsler Memory Scale (SMD = −1.286 [95% CI:−2.110 to −0.462]), and had longer P300 latency (SMD = 1.124, [95% CI: 0.746−1.502]). Qualitative analysis of neuroimaging data indicated that drug‐naïve individuals diagnosed with hypothyroidism have numerous structural and functional alterations. Meta‐regressions showed that the results are dependent on both hormone levels and sociodemographic variables. Neurocognitive difficulties in individuals with hypothyroidism is a complex phenomenon that requires further investigation.

**Highlights:**

- Neurocognitive impairment (NCI) occurs in about one third of patients with hypothyroidism.
- Thyroid‐stimulating hormone level is negatively associated with neuropsychological test results.
- Memory functions in patients with hypothyroidism were lower than in the control group.
- Sociodemographic and clinical characteristics were moderators of NCI.
- Studies indicate abnormalities in the structure and metabolism of the central nervous system in patients with hypothyroidism.

## 1 INTRODUCTION

Neurocognitive impairment (NCI) in people with medical problems is a growing clinical and social problem.^1^, ^2^ A number of studies indicate that various factors are associated with the deterioration of neurocognitive functioning, such as treatment,^3^, ^4^, ^5^ mood,^6^ or various biological mechanisms that directly or indirectly affect the central nervous system (CNS).^7^ NCI may adversely impact patients’ quality of life^8^ or worsen their adherence to medical recommendations.^9^

The data indicate that individuals diagnosed with hypothyroidism (HT) also have difficulties with neurocognitive functioning,^10^, ^11^ which includes various domains such as memory or executive functions.^12^ In addition to NCI, a number of other symptoms involving the CNS can be observed in this clinical group, such as depression or coma.^13^ Moreover, studies conducted on animal models confirm the negative effect of HT on the functioning of the CNS.^14^, ^15^

Recent reviews and meta‐analyses focusing on odds ratios and risk ratios have indicated no association between NCI and HT.^16^, ^17^ In addition, individual participant data analysis conducted on a sample of 74,565 participants did not show an association between subclinical hypothyroidism (SCH), HT, and NCI; however, overall participants’ median age was very high (74 years), and many people were diagnosed based solely on their thyroid‐stimulating hormone (TSH) level.^18^ It can be assumed that NCI may be more pronounced in the clinical group that has a medically confirmed diagnosis, especially when it is related to severe HT symptoms. However, these results are not consistent with the results of numerous case–control studies in which the selection of participants was much more precise. Previous meta‐analyses focused primarily on odds ratios,^12^, ^17^, ^19^ and risk ratios,^16^ omitting, for the identified limitations in the available literature, as well as the need for an in‐depth analysis of the topic discussed, contributed to an attempt at a comprehensive assessment of NCI in a group of people with HT using various types of indicators. The current systematic review focuses on the following issues:

1. Assessment of the frequency of NCI in patients with HT.
2. Assessment of the strength of the relationship between the level of select thyroid hormones and the results on neuropsychological tests.
3. Assessment of the intensity of NCI in subsequent neurocognitive domains in people with HT by analyzing the results of neuropsychological tests in case–control studies.
4. Assessment of differences in the structure and functioning of the CNS in people with HT by analyzing case–control studies using various neuroimaging techniques.

## 2 MATERIALS AND METHODS

### 2.1 Search strategy

This review was preregistered with international prospective register of systematic reviews (PROSPERO) (registration number CRD42023451054) prior to data collection. The list of excluded articles and all research components will be published on the registration website. The database search was carried out between August 3 and 8, 2023, with no time restrictions, according to the preferred reporting items for systematic reviews and meta‐analyses (PRISMA) protocol. The update was performed between January 21 and 22, 2025 using the same search methodology, but was limited to records that were published since 2023. The following databases were searched: EBSCO (Academic Research Source eJournals, Academic Search Ultimate, APA PsycArticles, ERIC, Health Source—Consumer Edition, Health Source: Nursing/Academic Edition, MasterFILE Premier, and MEDLINE), PubMed, ScienceDirect, EMBASE (update), the Cochrane Library, and DARE.

A logic grid was created using synonyms for hypothyroidism (Hypothyroidism OR Thyroid Stimulating Hormone Deficiency OR TSH Deficiency OR Hashimoto) and cognitive impairment (Cognitive Dysfunction OR Cognitive Impairment OR Cognitive Disorder OR Mild Cognitive Impairment OR MCI OR Cognitive Decline OR Mental Deterioration OR Memory OR Executive Functions OR Attention OR Processing speed OR Fluency OR Fog OR Dementia). Keywords were selected using Medical Subject Headings (MeSH) terms and additional terms previously identified in the literature on neurocognitive functioning.

Only English‐language reports published in peer‐reviewed scientific journals were included in the systematic review, although no restrictions were applied regarding the study sample's nationality. The decision to focus exclusively on sources that have undergone rigorous double‐blind peer review stems from the inclusion of only the highest quality data. Sources in languages other than English were excluded due to concerns about potential errors resulting from translation. In addition, references and review articles were analyzed to identify additional potential data sources. In the event of difficulties with access to the full‐text reports, the authors were contacted via e‐mail and/or ResearchGate. In the absence of a response within 1 week, the article was excluded from further review.

### 2.2 Selection criteria

**FIGURE 1:**
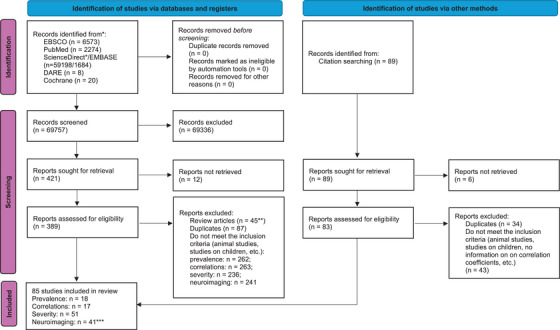
Flow chart of subsequent stages of data selection. ^*^For ScienceDirect, due to search limitations, three reviews were performed: the first one used the following keywords: (Hypothyroidism OR Thyroid‐Stimulating Hormone Deficiency OR TSH Deficiency OR Hashimoto) AND (Cognitive Dysfunction OR Cognitive Impairment OR Cognitive Disorder OR Mild Cognitive Impairment): 8743 results; (Hypothyroidism OR Thyroid‐Stimulating Hormone Deficiency OR TSH Deficiency OR Hashimoto's) AND (MCI OR Cognitive Decline OR Mental Deterioration OR Memory OR Executive Functions): 15,542 results; (Hypothyroidism OR Thyroid‐Stimulating Hormone Deficiency OR TSH Deficiency OR Hashimoto's) AND (Attention OR Processing speed OR Fluency OR Fog OR Dementia) 34,913 results. ^**^Including duplicates. ^***^The number of studies does not add up due to the use of different neuroimaging methods in selected studies: *From*: Page MJ, McKenzie JE, Bossuyt PM, Boutron I, Hoffmann TC, Mulrow CD, et al. The PRISMA 2020 statement: An updated guideline for reporting systematic reviews. *BMJ* 2021;372: n71. doi: 10.1136/bmj.n71. For more information, visit: http://www.prisma‐statement.org/.

First, both authors performed a screening of titles and abstracts in parallel using predefined selection criteria (see Population, Exposure, Comparison, Outcomes; PECO below). Screeners were blinded to one another's decisions. Each author created a database of articles deemed eligible for inclusion in the next stage by copying citations to an Excel sheet. After this stage, the screening results were compared, but no report was excluded. In the next stage, full versions of the texts identified as potentially relevant to the subject of the systematic review were carefully examined. Using the list of sources in the Excel file, both authors independently and in a blinded manner assessed whether the subsequent texts met the inclusion criteria. The results were compared and discussed. The process for selecting reports in this phase is shown in Figure 1.

Before starting the screening, criteria were defined in accordance with PECO, which were to be used to create a database of articles:

1. Population: Adults with medically confirmed diagnosis of HT. At the registration level, it was assumed that people who declared a diagnosis of HT would also be included; however, in order to maintain the greatest possible homogeneity of the group, it was decided to only include studies that contained information on confirmation of the diagnosis by a physician or results of hormonal tests.
2. Exposure: Both studies in which the subjects were treated with levothyroxine (LT4), as well as those describing the results of studies in which pharmacotherapy was not initiated, were included in the review. Both treatment status and pharmacotherapy, including the duration and dose of LT4, were to be used as moderators. This step was taken because data from the literature on other health problems indicate that, despite treatment, NCI may persist in various cognitive domains.
3. Comparison: For the purposes of the initial screening, it was not assumed that comparators would be used as the basic inclusion/exclusion criterion. Only at the later stages of study selection in the analysis of the severity of NCI in the group of people with HT were studies included in the analyses that compared the results of people with HT to other healthy/euthyroid people—in this case, only people without a diagnosis of chronic diseases and other health problems that could affect the level of neurocognitive functioning were included.
4. Outcomes: Results of neuropsychological tests. In the case of outcomes, neither a specific cognitive domain nor a selected method was decided, but rather the broadest possible description of the topic. In the case of studies using different neuroimaging methods, they were included in further analyses, regardless of the use of neuropsychological tests.

Studies using different research designs were included in the subsequent stages, but the present review describes the results of cross‐sectional comparisons. In the case of interventional studies, only the baseline or the measurement in which patients were HT was considered. Neither case studies nor case series were included in the analyses. Review articles were included in a separate pool for citation review.

Using the above criteria, a database of articles was created, which were subjected to further selection. For the purposes of subsequent analyses, the following criteria were applied:

#### 2.2.1 Prevalence

The following criteria were used to assess the prevalence of NCI in patients with HT:

1. The study must describe the methods of diagnosis of NCI based on neuropsychological tools.
2. The percentage of the sample diagnosed with NCI must be reported.
3. The sample selection must be random—the inclusion criteria did not include information about excluding patients below the cutoff point for the diagnosis of NCI.

Relationship between hormones and results of neuropsychological tests:

In the case of strength of the relationship between thyroid hormones and indicators of NCI in patients with HT, the inclusion criteria only permitted studies that:

1. Reported the strength of this relationship using correlation coefficients, and
2. the correlation coefficients concerned patients with HT specifically (i.e., not the general population).

#### 2.2.2 Severity of NCI

1. The study had to report a comparison of patients with HT with healthy/euthyroid controls.
2. In the case of longitudinal and experimental studies, only baseline measurements were taken into account.

#### 2.2.3 Neuroimaging studies

In the comparison of results of studies using different neuroimaging techniques, only case–control studies were taken into account. For neuroimaging data, Condition O (the study must have used validated objective neuropsychological methods to assess neurocognitive functions) was not necessary. In addition, the following additional criteria were adopted:

1. The study had to report a comparison of HT patients with healthy/euthyroid controls.
2. In the case of longitudinal and experimental studies, only baseline (or when patients were HT) measurements/comparisons were taken into account.

### 2.3 Data extraction

The authors independently extracted data from each study using predefined Excel sheet. The authors were blinded during this process; upon completion, the extracted data tables were compared and a final version was agreed upon in discussion. For all studies included in the review, the following data was extracted: authors, year of publication, the country where the study was conducted, sample size, sociodemographic variables (percentage of women, mean age, and mean number of years of education), clinical indicators (mean time since diagnosis and mean disease severity assessed using validated tools), mean TSH, free triiodothyronine (fT3), and free thyroxine (fT4) levels and levothyroxine doses, body mass index (BMI), TSH ranges, and the main study findings. For the subsequent analyses, the following results were also extracted:

1. The percentage of HT patients with an NCI diagnosis.
2. Correlation coefficients between hormone levels and neuropsychological test results.
3. The mean values and standard deviations (SDs) of neuropsychological tests results in patients with HT and healthy/euthyroid control groups.
4. The means and SDs of results obtained with neuroimaging techniques in patients with HT and healthy/euthyroid participants.

For selected analyses, the information about depressive symptoms, NCI assessment method, and definition of NCI was extracted. Because our analyses included cross‐sectional comparisons, it was decided not to extract either funding or conflict of interest data—the risk of potential influence, for example, from industry, on the results is very low.

### 2.4 Quality assessment

To evaluate the quality of the selected studies, we used the Joanna Briggs Institute Critical Appraisal Checklist for analytical cross‐sectional and case–control studies.^20^ Each author independently scored each study—interrater compatibility was satisfactory and scoring differences were reconciled through discussion. The quality assessment results for the studies included in the systematic review are provided in Table S1.

### 2.5 Statistical analysis

Pooled analysis was performed only when multiple studies used the same NCI assessment method. This decision was based on the fact that different neuropsychological tests assess different neurocognitive domains/functions, and including scores from different methods could bias the results and could constitute a potential source of heterogeneity. According to the Cochrane guidelines (https://training.cochrane.org/handbook/current), meta‐regression is recommended when data are available from a minimum of 10 studies. However, due to the expected small number of replications, as well as problems with reporting appropriate data, we decided to perform this type of calculation with a minimum of five studies, what was indicated in the pre‐registration. Accordingly, we performed pooled analyses only when data were available for at least five studies using the same NCI measurement method.

No imputation methods were planned for addressing missing data—they will be omitted in collective analyses. In the case when data are presented in other units, online tools will be used that will allow their conversion—for example, in the case of hormones, https://unitslab.com/ will be used. For the needs of the current review, it was not assumed that the results of, for example, regression analyses will be converted to correlation coefficients, due to the very frequent control of other variables (adjusted coefficients) that could distort the results of meta‐analyses. In the case of medians and standard errors, the data were incorporated into the meta‐analysis, but their influence on the results will be controlled in the next steps (jackknife analyses).

All meta‐analyses were performed using RStudio with the “meta” library. The overall rates and 95% confidence intervals (CIs) were calculated using a random‐effects model. Publication bias and outlier publications were identified using Baujat and funnel plots. Additional sensitivity analysis was performed using the jackknife method. In this type of analysis, meta‐analyses were performed by excluding individual studies one by one, thereby assessing the impact of the removed studies on both the effect size and the heterogeneity of the results. This also allowed us to assess the stability of the obtained results. The heterogeneity of effects among studies was estimated using the *Q* (Wald‐type) test and quantified using the *I* ^2^ test. Any *p*‐values < 0.05 were considered statistically significant. The “metareg” function was used to evaluate potential moderators. For meta‐analysis of NCI prevalence, the proportions were assessed; for correlations—*r*. In the case of analysis of differences between subgroups, the use of standardized mean difference (SMD) is assumed.

### 2.6 Ethics

In the preparation of the systematic review, no contact was made with the study participants and we relied only on published materials. Therefore, the consent of the local ethics board was not needed.

## 3 RESULTS

### 3.1 Prevalence of NCI in individuals with HT

The systematic review identified 18 articles that met the inclusion criteria.^21^, ^22^, ^23^, ^24^, ^25^, ^26^, ^27^, ^28^, ^29^, ^30^, ^31^, ^32^, ^33^, ^34^, ^35^, ^36^, ^37^, ^38^ A total of 2123 HT patients (including SCH) participated in the studies. The mean age of patients ranged from 30.3 years^21^ to 79.2 years.^22^ NCI was diagnosed using various test methods and its prevalence ranged from 0%^23^ to 95.2% (Digit Span Test [DST]).^24^ The very high variability of results seems to reflect either various difficulties in neurocognitive functioning experienced by HT patients (i.e., impairments in different neurocognitive domains such as short‐term/working memory; see the very high prevalence in the case of the DST), or may result from differences in the samples structures, for example, in the mean TSH concentration or age. A detailed description of the studies included in the systematic review is included in Table S2.

Many tools were used in single studies. Therefore, based on the data collected, we decided to conduct a meta‐analysis covering only the results obtained with the Mini‐Mental State Examination (MMSE): it was the only tool that was used in sufficient number of studies, and thus it was possible to extract adequate data for pooled analysis.

#### 3.1.1 Prevalence of NCI in HT: meta‐analysis of MMSE studies

**FIGURE 2:**
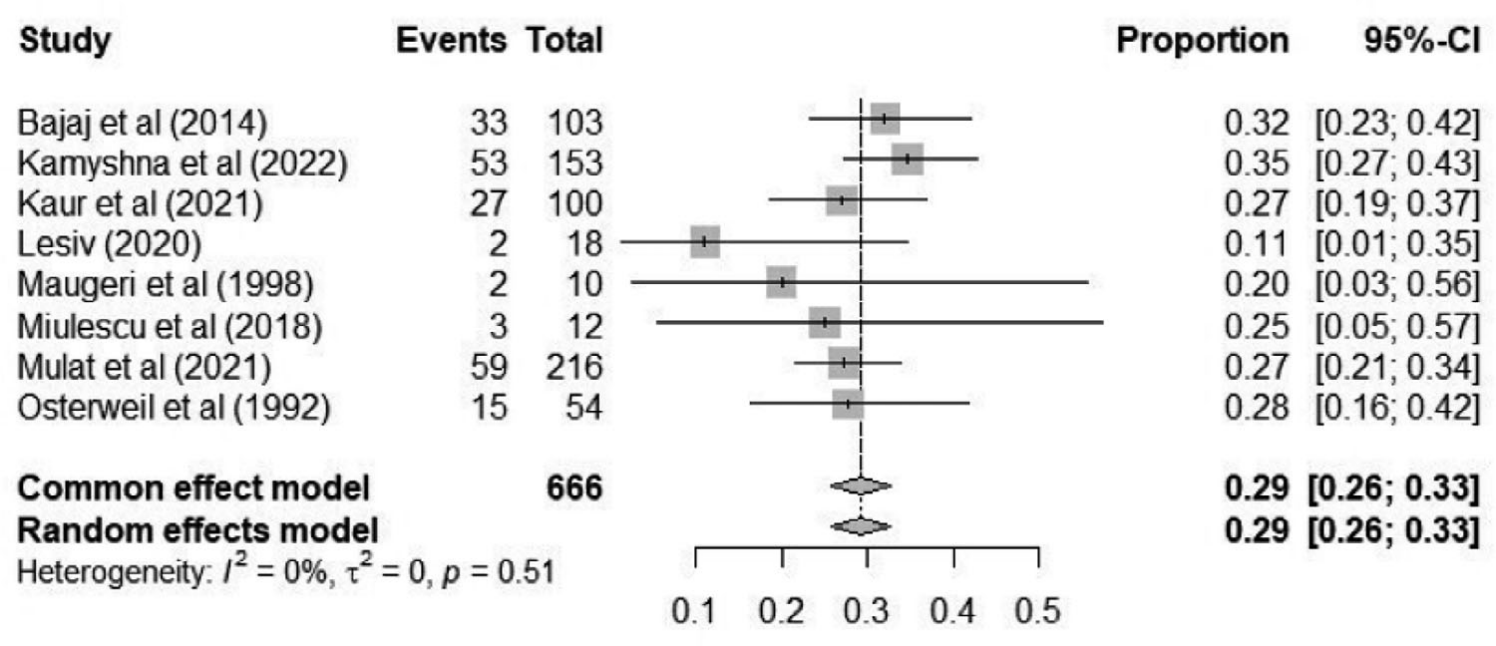
Prevalence of neurocognitive impairment in hypothyroid patients assessed with Mini‐Mental State Examination (MMSE): forest plot.

The eight studies using the MMSE to diagnose NCI identified in the database search as meeting the inclusion criteria were pooled to give an overall point prevalence (PR) estimate of 0.29 (95% CI: 0.26–0.33; see Figure 2) of the total analysed population. Statistically significant heterogeneity was not observed between the studies (*Q *= 6.26; *p *> 0.05). See also Figures S1 and S2 for the Baujat and funnel plots, respectively.

Subsequently, sensitivity analyses were performed using the jackknife method (see Table S3). The results of the analyses indicated that removing subsequent studies included in the meta‐analysis did not result in change in heterogeneity and PRs: they ranged from 0.275 to 0.3.

### 3.2 Correlations between thyroid hormone levels and NCI assessed with standardized methods

The systematic review identified 17 articles reporting correlations between thyroid hormone levels and NCI that met the inclusion criteria.^23^, ^24^, ^31^, ^33^, ^39^, ^40^, ^41^, ^42^, ^43^, ^44^, ^45^, ^46^, ^47^, ^48^, ^49^, ^50^, ^51^ In the case of longitudinal studies or experimental designs, the focus was only on data from the first measurement (baseline) or the measurement in which the subjects were HT. A total of 860 people diagnosed with HT (including SCH) participated in the studies. The samples varied in terms of treatment use, mean age (from 28 in Menicucci et al.^39^ to 68.6 in Osterweil et al.^33^) and percentage of women (from 46% in Osterweil et al.^33^ to 100% in, e.g., Menicucci et al.^39^). As in the case of PRs, NCI was examined using various test methods, with the most frequently used neuropsychological test being the MMSE. Despite the various test methods used to measure NCI, the studies mostly indicated that TSH was negatively related to neurocognitive functioning (the higher the TSH level, the greater the difficulties in neurocognitive abilities). A lack of statistically significant correlation was also a frequent result, which in some cases could have been be due to group size.^44^ Most of the results focused on TSH, but the collected data also reported correlations between fT3, fT4, T3, or T4 and neurocognitive tests.^33^, ^47^ Detailed data on the studies identified in the systematic review are presented in Table S4.

#### 3.2.1 Relationship between TSH and MMSE results in HT: meta‐analysis

**FIGURE 3:**
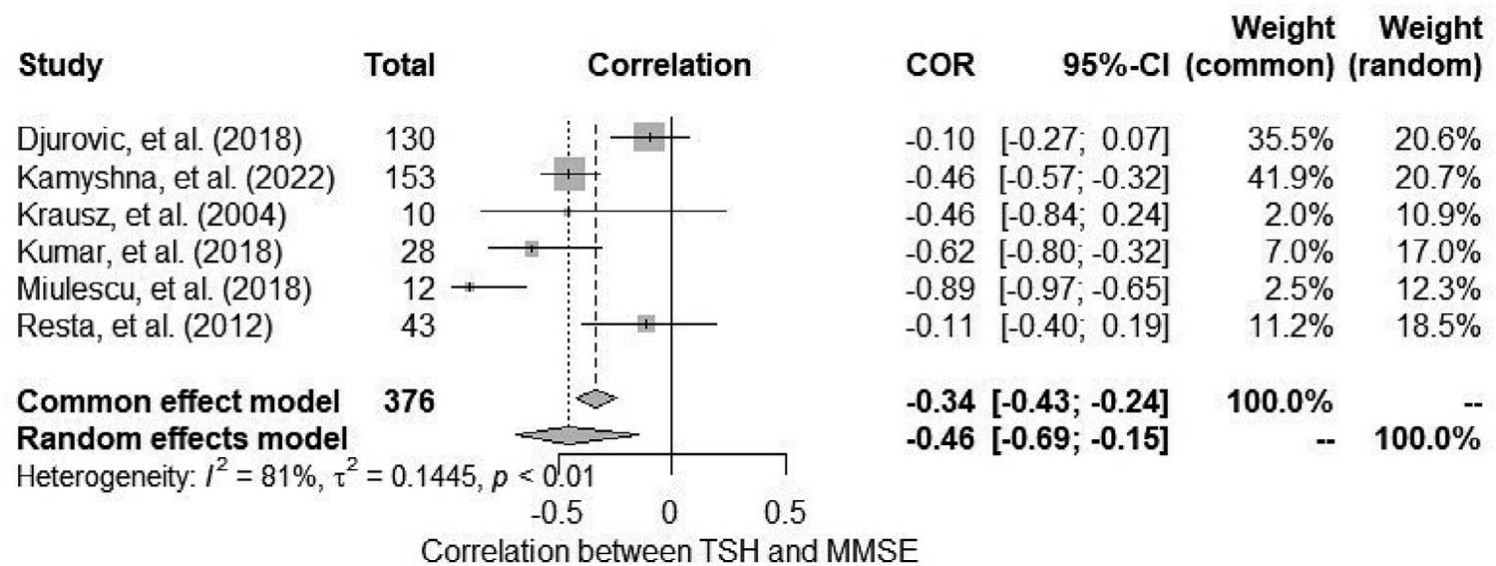
Correlations between thyroid stimulating hormone (TSH) levels and Mini‐Mental State Examination (MMSE) scores in hypothyroid patients: forest plot.

Only the correlation values between TSH and the MMSE were repeated in a sufficient number of studies. The six studies identified in the database search as meeting the inclusion criteria were pooled to give an overall correlation of –0.46 (95% CI: –0.69, –0.15), (see Figure 3). Statistically significant heterogeneity was observed between the studies (*Q *= 26.88; *p *< 0.001). See also Figures S3 and S4 for the funnel and Baujat plots.

Further analyses focusing on the sources of heterogeneity were impossible to perform due to deficiencies in data reporting as well as the small number of studies. Visual inspection indicated three significant outlier studies—in Djurovic et al.^42^ and Resta et al.,^48^ the correlation coefficients were at the level of –0.1, whereas in the case of Miulescu et al.,^31^ the data indicated a very strong relationship (*r* = –0.89). In the next step, the jackknife method (Table S5) was used, which confirmed that when the above‐mentioned studies are removed from the meta‐analysis, the largest change in correlation coefficients occurs: changes in the strength of correlation oscillate around0.1. In addition, removing Djurovic et al.^42^ resulted in the greatest decrease in heterogeneity. However, with this study removed, the results are still not homogeneous.

### 3.3 Severity of neurocognitive impairment: test‐by‐test analyses

The systematic review identified 51 studies that met the inclusion criteria.^21^, ^23^, ^24^, ^25^, ^27^, ^28^, ^29^, ^33^, ^36^, ^39^, ^41^, ^42^, ^43^, ^45^, ^46^, ^47^, ^48^, ^50^, ^51^, ^52^, ^53^, ^54^, ^55^, ^56^, ^57^, ^58^, ^59^, ^60^, ^61^, ^62^, ^63^, ^64^, ^65^, ^66^, ^67^, ^68^, ^69^, ^70^, ^71^, ^72^, ^73^, ^74^, ^75^, ^76^, ^77^, ^78^, ^79^, ^80^, ^81^, ^82^, ^83^ Both authors also agreed include the study by Baldini et al.^52^ in the systematic review, which analyzed groups of people with goiters. For a similar reason, the studies by Leyhe et al.,^46^, ^68^ in which the control group consisted of people with goiters and after thyroid surgery who were healthy/euthyroid, were also included. However, special attention was paid to them in the pooled analysis, and especially in the jackknife analyses.

The studies included a total of 2346 patients diagnosed with HT (including SCH) and 11,777 control group participants. The mean age ranged from 20.3^53^ to 80.1^54^ years. The causes of HT reported in the studies included, among others, autoimmune thyroiditis, postoperative HT, or severe forms of congenital HT. Comparisons of neurocognitive functioning were made using a wide variety of tools, including screening scales, tests assessing specific cognitive functions, or entire batteries. A detailed description of the studies’ results is included in Table S6.

Results presented using medians and the interquartile range (IQR; e.g., Menicucci et al.^39^) or means and standard errors of the mean (SEMs) were also included in the meta‐analyses, and the effect of their inclusion was assessed at a later stage during Jackknife analyses. A description of the results, including the meta‐analyses of Trail‐Making Test Parts A and B (TMT A and B), DST, and Fluency, are included in File S1.

#### 3.3.1 The Mini‐Mental State Examination

**FIGURE 4:**
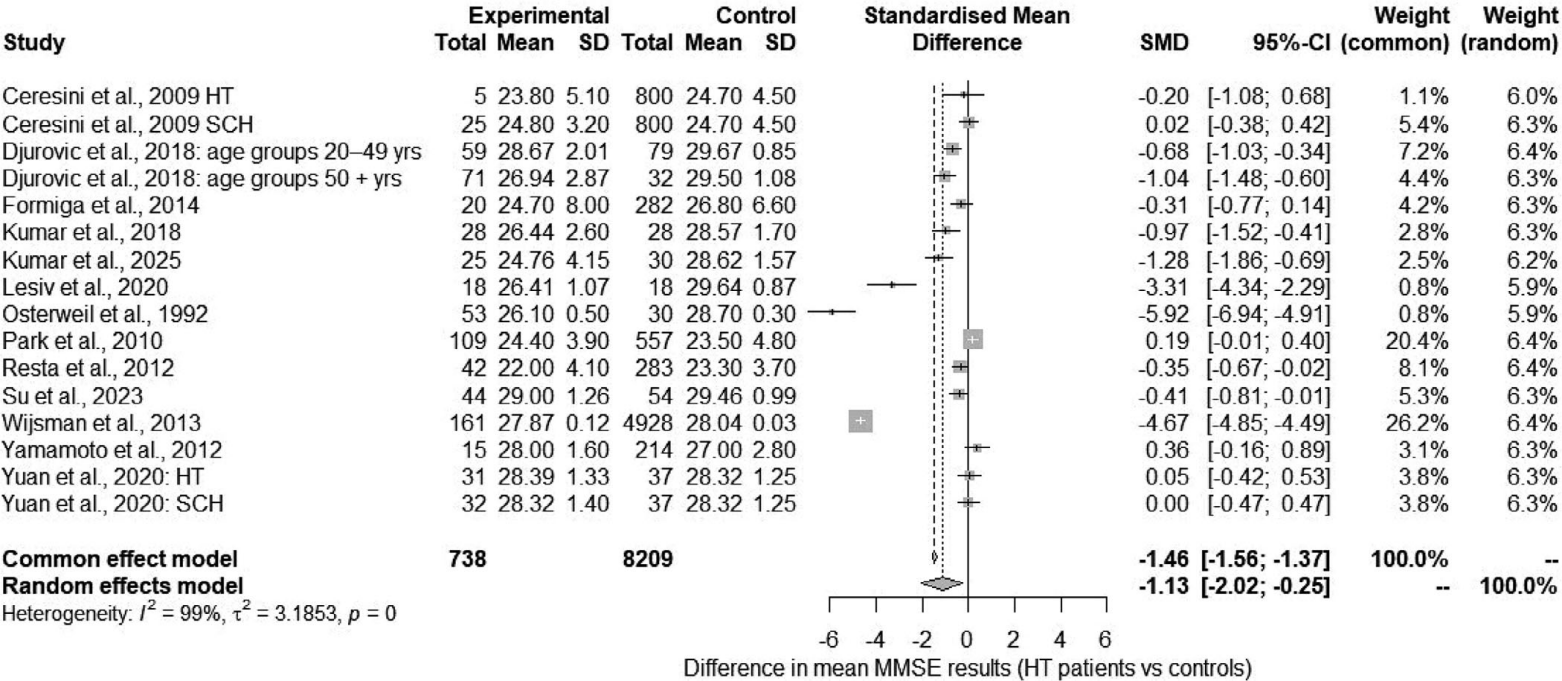
Severity of neurocognitive impairment assessed with Mini‐Mental State Examination (MMSE): forest plot.

For the study by Yamamoto et al.,^54^ only the baseline measurement was used for the meta‐analysis. For other studies, comparisons were made taking into account, among others, division by patient age^42^ or diagnosis (SCH and HT^51^). A total of 16 comparisons were included in the meta‐analysis, yielding a pooled effect size of −1.134 (95% CI: −2.020, −0.248), (see Figure 4). The results of the analyses indicated statistically significant heterogeneity between the studies (*Q* = 1844.14, *p* < 0.001). See Figures S5 and S6 for the Baujat and funnel plots.

None of the following were statistically significant moderators: percentage of women, mean sample ages, mean years of education, mean TSH level, mean BMI, quality of included studies, or mean fT4 level (see File S2 for detailed description). Mean fT3 (five comparisons) was a statistically significant moderator (*QM*(1) = 12.236, *p* < 0.001), (see Figure S7 for the bubble plot). When fT3 was taken into account, the results were homogenous (*Q* = 1.833, *p* = 0.608).

#### 3.3.2 Wechsler Memory Scale (WMS)

**FIGURE 5:**
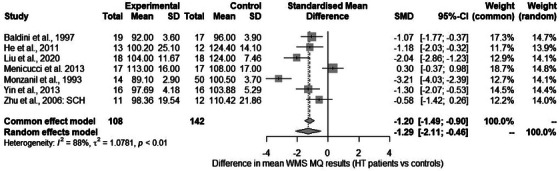
Severity of neurocognitive impairment assessed with Wechsler Memory Scale (WMS) memory quotient (MQ): forest plot.

We identified seven studies in which the memory quotient (MQ) results were reported. The meta‐analysis showed an effect size of −1.286 (95% CI: −2.110, −0.462), (see Figure 5 for the forest plot and Figures S8 and S9 for the Baujat and funnel plots). This result was heterogeneous (*Q* = 48.54, *p* < 0.001). Further analyses showed that none of the variables included were statistically significant moderators (see also File S2 for further details).

Analyses were also performed for the Mental Control subscale. In the study by Menicucci et al.,^39^ the results were reported using medians and IQR. The pooled analysis showed an effect size of −1.075 (95% CI: −1.625, −0.525), (see Figure S10 for the forest plot and Figures S11 and S12 for the Baujat and funnel plots, respectively). This result was heterogeneous (*Q* = 10.99, *p* = 0.027), and it was not possible to identify its source (see also File S2 for detailed meta‐regression results).

Next, we carried out the jackknife sensitivity analysis. We removed studies one by one while controlling the effect size and the parameters related to the heterogeneity of the results. Detailed results are presented in Table S7.

### 3.4 Electromyography, evoked potentials, and electroencephalography

The search procedure identified 13 studies that used evoked potentials (both visual and auditory), two studies using electromyography (EMG), and one study that focused on electroencephalography (EEG) changes.^24^, ^33^, ^39^, ^72^, ^73^, ^84^, ^85^, ^86^, ^87^, ^88^, ^89^, ^90^, ^91^, ^92^, ^93^, ^94^ A detailed description of the studies is provided in Table S8. Qualitative descriptions of the studies presenting data obtained using EEG, EMG, visual, and auditory evoked potentials are presented in File S3.

#### 3.4.1 evoked potentials and electroencephalography: meta‐analyses

**FIGURE 6:**
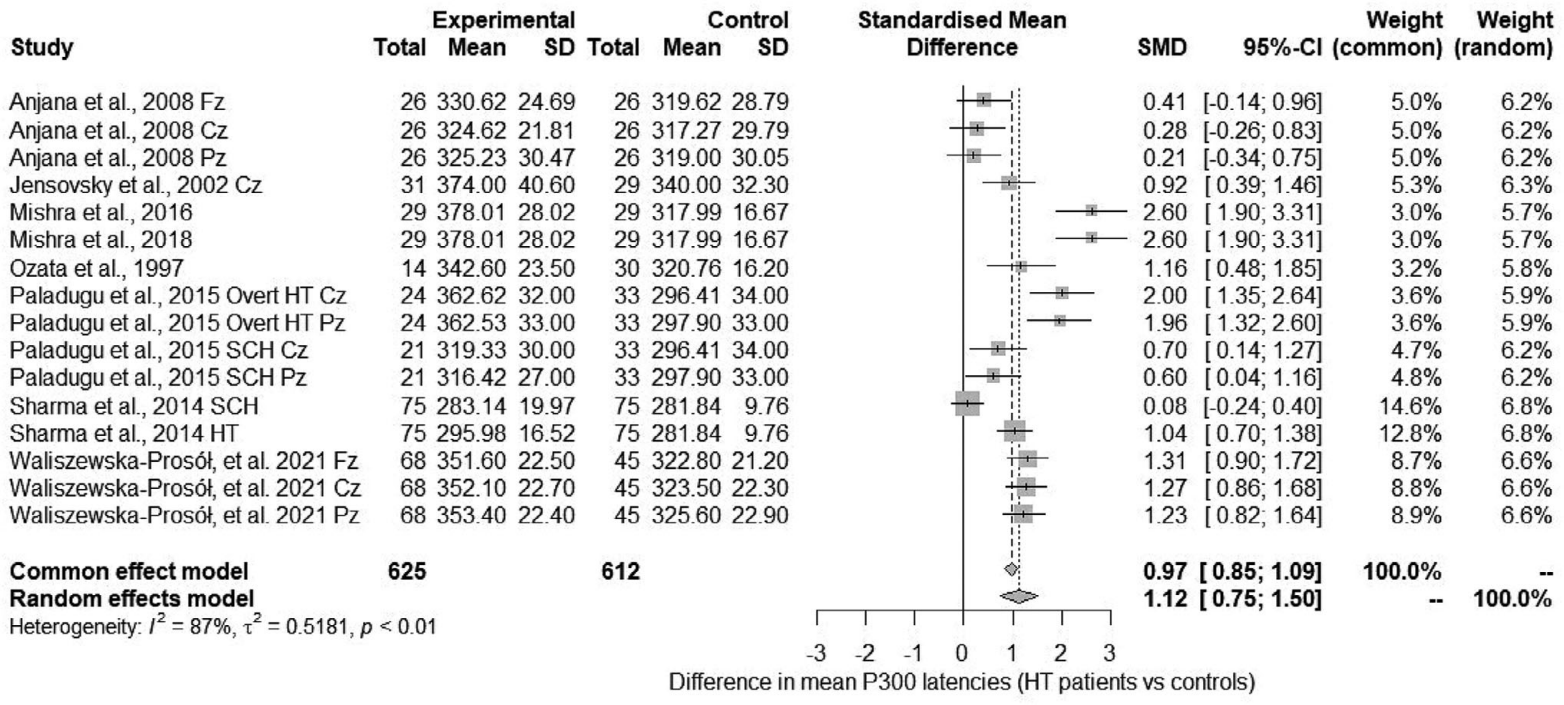
Differences in P300 latencies: forest plot.

The description of the results including meta‐analyses of N200 latencies and P300 amplitude is included in File S4. P300 latency was reported in eight articles.^72^, ^73^, ^85^, ^86^, ^90^, ^91^, ^93^, ^94^ In some studies, mean values were given,^72^, ^73^, ^93^ while in others, these values were given for consecutive electrodes. Paladugu et al.^91^ and Sharma et al.^93^ gave the values for HT and SCH separately. A total of 16 comparisons were included in the meta‐analysis (see Figure 6).

The results of the meta‐analysis indicated a statistically significant between‐group difference with an effect size of SMD = 1.124 (95% CI: 0.746, 1.502), (see Funnel and Baujat plots in Figures S13–S14) and statistically significant heterogeneity (*Q* = 116.27; *p* < 0.001). Further analyses showed that female ratio (*QM*(1) = 0.003, *p* = 0.96; mean age, *QM*(1) = 0.573, *p* = 0.45; mean TSH levels, *QM*(1) = 3.213, *p* = 0.07; mean fT4 levels, k = 8, *QM*(1) = 0.102, *p* = 0.75; and mean fT3 levels, k = 8, *QM*(1) = 0.837, *p* = 0.36, were not statistically significant moderators. Jackknife analyses are presented in Table S9.

### 3.5 DTI, PET, MRI, fMRI, MRS, and SPECT studies

The systematic review identified a total of 25 studies^36^, ^44^, ^45^, ^46^, ^47^, ^50^, ^57^, ^62^, ^66^, ^69^, ^80^, ^82^, ^83^, ^94^, ^95^, ^96^, ^97^, ^98^, ^99^, ^100^, ^101^, ^102^, ^103^, ^104^, ^105^ that used data obtained from diffusion tensor imaging (DTI), positron emission tomography (PET), magnetic resonance imaging (MRI), functional MRI (fMRI), single‐photon emission computed tomography (SPECT), and magnetic resonance spectroscopy (MRS). Due to data limitations, only a qualitative description of the results of these studies was possible.

Differences in brain structures between HT and euthyroid controls assessed using MRI were described in seven studies.^36^, ^46^, ^47^, ^50^, ^103^, ^104^, ^105^ Their results indicate a number of CNS abnormalities in HT patients, including:

1. Cerebellum (decreased cerebellar volume^105^ (VI–VIIIa vermis)^103^ and increased gray matter volume (GM)bilateral cerebellar Crus I,^36^
2. Basal ganglia (decreased pallidum volumes^103^),
3. Limbic system (decreased volume within the right hippocampus^104^ and decreased GM in cingulate gyrus^50^),
4. Parietal lobe (decreased GM—left postcentral gyrus^105^ and decreased GM—precuneus^50^),
5. Frontal lobe (decreased white matter volume (WM)—right precentral gyrus^105^ and right inferior and middle frontal gyrus,^105^ decreased GM—the left middle frontal gyrus,^36^ left dorsolateral superior frontal gyrus,^36^ left supplementary motor area,^36^ orbital part of the right superior frontal gyrus,^36^ the bilateral prefrontal cortex,^50^ increased GM—left precentral gyrus^36^),
6. Occipital lobe (decreased WM—right inferior occipital gyrus^105^),
7. Temporal lobe (decreased WM—right inferior temporal gyrus,^105^ decreased GM—right superior temporal gyrus,^36^ and left middle temporal gyrus^50^),
8. Insula (decreased GM—insula^50^).

**FIGURE 7:**
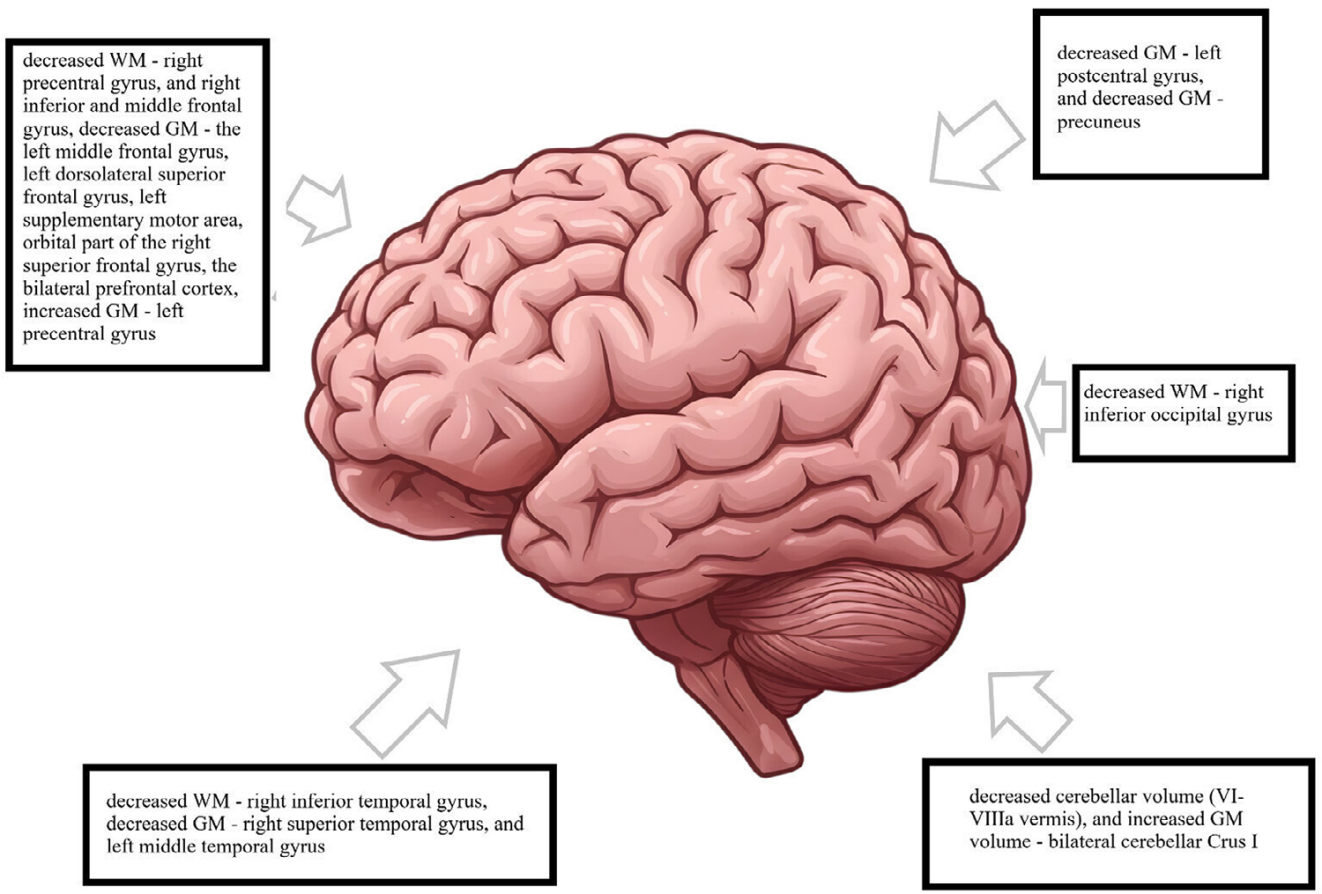
Brain regions altered in people with hypothyroidism. Image download from: https://pngtree.com/freepng/realistic‐human‐brain‐illustration_20753430.html.

Some of the structures are marked in Figure 7; details are presented in Table S8. However, it should be noted that some studies, where patients were on stable LT4 treatment, indicated no differences between groups.^46^, ^47^

Studies on functional CNS differences between individuals diagnosed with HT and euthyroid controls were performed mostly on drug‐naïve samples. Analyses performed using DTI revealed a number of differences in fractional anisotropy as well as axial diffusivity of the WM, suggesting a demyelination process.^57^, ^80^, ^95^ Studies using PET also observed a number of statistically significant differences in activity in structures such as the amygdala, hippocampus, and regions of the anterior cingulate cortex.^96^, ^97^ In turn, analyses performed using SPECT indicated, among other things, decreases in regional cerebral blood flow, particularly in the parietal and occipital areas.^44^, ^98^, ^99^ Studies using MRS showed a decrease in the *N*‐acetylaspartate/total creatine ratios, as well as an increase in the concentrations of glutamate and choline in the posterior cingulate and parietal cortex in HT patients.^66^, ^100^, ^102^ In addition, reduced glutamate/creatine and myo‐inositol/creatine ratios in the hippocampus were found.^101^ In the case of fMRI, various assessment paradigms were used—the studies analyzed both resting‐state networks and CNS activation during specific neurocognitive tests. Most studies involving resting‐state networks were conducted on drug‐naive samples and showed statistically significant differences between HT patients and controls, including reduced connectivity strength between the left amygdala and right middle temporal gyrus, increased functional connectivity between the right cerebellar Crus I and the left precentral gyrus, right frontoparietal attention network, as well as alterations in the default mode network.^36^, ^45^ Quinque et al.^47^ conducted a study on individuals treated with LT4 and found no differences between the groups. In the case of analyses involving CNS activation during neuropsychological testing, various research methods were used: 4‐digit backward and forward recall task,^62^ memory encoding task,^47^ n‐back task,^50^, ^83^ and Stroop task.^82^ In the case of the n‐back task, both studies observed lower activation intensities in the frontal cortex.^50^, ^83^ He et al.^62^ observed between‐group differences in task‐induced deactivation in the bilateral medial prefrontal cortex, posterior cingulate cortex, and left inferior partial lobule during the 4‐digit backward and forward recall trials. Yin et al.^50^ observed lower activation of the prefrontal cortex, anterior and posterior cingulate cortex, precuneus, insula, and caudate nucleus.

The review also identified a number of correlations between CNS function and hormone levels. TSH was positively associated with choline levels (posterior parietal cortex region)^100^ and negatively associated with GM density in the left anterior cingulate cortex,^47^ and regional volumes and percentage of blood oxygen level–dependent (BOLD) signal changes in the prefrontal cortex, anterior cingulate cortex, and precuneus.^50^ T4 was negatively associated with choline levels in the posterior parietal cortex region and dorsolateral prefrontal cortex.^66^ In turn, fT4 was associated with reduced connectivity strength between the left amygdala and right middle temporal gyrus as well as between the subcallosal cortex and right frontal pole.^47^ fT3 levels were positively associated with *N*‐acetylaspartate/total creatine ratios in the posterior cingulate gyrus region and in the parietal WM area.^100^ Quinque also observed a positive association between anti‐thyroid peroxidase antibodies (anti‐TPO) levels and connectivity between the subcallosal cortex and left parahippocampal gyrus.^47^ In addition, a number of correlations were also observed between the severity of depressive symptoms and activity in the bilateral middle frontal gyrus, right subgenual and dorsal anterior cingulate gyrus,^96^ medial frontal gyrus γ‐aminobutyric acid (GABA+) concentration,^69^ GM density in the right postcentral gyrus, left superior frontal gyrus, left cuneus, GM density in the left middle temporal gyrus,^47^ and fractional anisotropy of the right and left cingulum.^95^ Details are presented in Table S8.

## 4 DISCUSSION

The first part of the systematic review showed, that depending on the methods used and the populations studied, the prevalence of NCI ranged from 0% (Planning^23^) to 95.2% (DST^24^). The results of the meta‐analysis of MMSE prevalence rates turned out to be homogeneous: regardless of the differences in the structure of the analyzed samples, the prevalence of NCI was about one in three HT patients. This result is similar to the rates presented by Manly et al.,^106^ who analyzed a representative sample of 3496 people 65 years of age and older. Their results indicated that dementia was present in 10% and mild cognitive impairment in 22%. It should be noted that in some studies included in the current meta‐analyses, the mean age was <65 years,^23^, ^29^, ^32^ whereas in Maugeri et al.,^30^ it was not reported. In turn, analyses conducted in a sample of patients with type 2 diabetes indicated that NCI assessed using the Montreal Cognitive Assessment (MoCA) is present in up to 46.6% of the sample, with the results being heterogeneous.^107^ In Cannon et al.,^108^ the NCI index in the sample of patients with heart failure was 0.43, although the authors collectively analyzed the results obtained using different tools, and NCI prevalence ranged from 10% to 79%. In Pankowski et al.,^109^ the prevalence of NCI assessed using the MoCA in the group of people with rheumatoid arthritis was 0.75, but this result was also characterized by heterogeneity. This finding may hold significant clinical relevance, as it indicates the need for neuropsychological assessment of patients both in the diagnostic process and in long‐term treatment with LT4. According to the results of studies conducted on other clinical groups, NCI may contribute to poorer functioning^8^ or may worsen adherence to medical recommendations.^9^

Previous reviews indicate an important role of hormones for NCI, including, for example, estrogen,^110^ or thyroid hormones—the results of the analysis conducted by Dolatshahi et al.^111^ showed that serum total and fT3 levels are significantly lower in patients with Alzheimer's disease (AD) compared to controls. Qualitative analysis of the data included in our review indicated, regardless of the methods used, a negative relationship between the TSH level and neuropsychological tests results. In addition, some studies indicated relationships between fT4 and NCI, as well as a lack of statistically significant relationships.^42^ Unfortunately, many studies did not provide the size of the correlation coefficients, focusing only on the statistical significance of the relationship, which is largely dependent on sample size. Meta‐analysis assessing the strength of the relationship between the mean TSH levels and MMSE results indicated a moderate, negative association characterized by statistically significant heterogeneity. It can be tentatively assumed that the heterogeneity may be partly due to the characteristics of the MMSE, which is a screening tool used to assess general NCI, while more homogeneous results may be obtained in analyses including tools limited to assessing select neurocognitive functions. It should be noted that the analyses conducted concern only patients with HT, the pooled analysis for the general population may also indicate a different strength of the relationship. More research is certainly needed to allow for a more precise assessment of the relationships between thyroid hormone levels and neuropsychological tests scores (see also Eslami‐Amirabadi & Sajjadi^112^).

The next step was a test‐by‐test meta‐analysis of results of studies comparing NCI in HT patients and healthy/euthyroid individuals. Our results indicated that, depending on the tool used, the effect sizes ranged from small (e.g., TMT B) to large (e.g., MMSE). Large effect size in MMSE is in line with other analyses focusing on chronic health problems, where statistically significant between‐group differences in MMSE results are observed, when compared to healthy individuals. Such decline was observed in patients with diabetes^113^ or rheumatoid arthritis.^114^ In addition, a statistically significant difference was observed between HT patients and the healthy/euthyroid control group, with a large effect size in the overall WMS score, and Mental Control subscale (measuring attention and working memory). It should be noted that the described difficulties occurred in patients diagnosed with goiter,^52^ and SCH,^83^ or receiving LT4 treatment.^82^ Memory impairments are present in many chronic health conditions,^115^ in some cases showing reversibility with appropriate treatment.^116^ To better describe the dynamics of NCI changes in HT, it seems necessary to analyze interventional studies as well as assess trajectories that would allow determining the percentage of patients in whom difficulties persist despite the treatment used. In the case of TMT A and B, DST forward and backward, as well as fluency, the CI values intersected 0, which regardless of the effect size indicated no differences between HT patients and euthyroid/healthy controls.

Test‐by‐test meta‐analyses describing the severity of NCI in the HT group were also characterized by statistically significant heterogeneity. For MMSE, meta‐regressions showed that only mean fT3 was a statistically significant moderator, and after taking it into account, the results were homogenous. Further analysis performed for TMT A showed that the mean sample age and TSH levels were statistically significant moderators. The positive association with TSH levels may indicate that this hormone is significant for the functions assessed using the TMT A, namely primarily psychomotor speed and visual scanning. For TMT B, which measures working memory, inhibition control, or set‐switching, meta‐regression indicated that the female ratio was a statistically significant moderator. After taking it into account, the results became homogeneous, but these values were reported in only five studies. The role of sociodemographic factors in explaining variability between outcomes is consistent with normative data (e.g., Specka et al.^117^), which indicate an increase in the average time of task completion with increasing age. Further analyses showed that fT4 values were a statistically significant moderator in the case of the DST backwards, but after taking it into account, the level of heterogeneity was still statistically significant. This may indicate that free thyroxine may be important for the neurocognitive processes assessed by this test, considering that forward and backward variants are characterized by differences in the neurocognitive processes involved (e.g., Gerton et al.^118^). For the remaining tests, no potential sources of heterogeneity could be identified.

The obtained results were also subjected to jackknife analyses. In the case of severity of NCI assessed with MMSE, removing studies with very large samples significantly affected SMD and heterogeneity. The differences in effect sizes were quite large, reaching even 0.3, while still remaining within the cutoff point for a large effect size. For TMT A, sensitivity analyzes showed that data from one of the studies^33^ significantly distorted the results: after its removal, the results indicated a statistically significant between‐group difference, and were homogeneous. Jackknife analyzes conducted for TMT B, showed that two studies^28^, ^71^ contributed to the lack of differences between groups. After their removal, the CIs did not cross 0, while the effect size remained small. In the case of the DST forward, removing studies did not significantly affect the level of heterogeneity or the appearance of statistically significant between‐group differences. The studies of Park et al.^76^ and Menicucci et al.^39^ had the greatest influence on the results of fluency. After removing it, the difference between HT patients and healthy/euthyroid controls became statistically significant to the detriment of HT patients, and the effect size was moderate. For WMS overall score, jackknife analyzes showed that when subsequent studies were removed, the result remained heterogeneous. Regardless of the fluctuations in the effect size, it was still large, and the difference between the groups was still statistically significant. In turn, in the case of subscale Mental Control, more than half of the studies could influence the heterogeneity. After removing these studies,^52^, ^82^, ^83^ SCH subgroup the result was homogeneous. Regardless of the committed study, the effect size remained large, and the CIs did not cross 0.

Next, the focus was on the results of studies using neuroimaging, in which the analysis of case–control studies showed a number of differences between HT patients and euthyroid controls in both structure and functioning of the CNS. Due to their small number and differentiated methodology, it was not possible to collectively analyze the EMG, EEG, and visual evoked potential results. More detailed analyses were performed for the auditory evoked potentials. An increase in the N200 wave latency was observed; however, this result was sensitive to the removal of subsequent studies—the effect size ranged from SMD = 0.635 to 1.05. In addition, in the case of this result, special caution should be exercised given that the results came from three studies (seven comparisons). Prolongation of the N200 wave latency is also observed, among other conditions, in schizophrenia,^119^ and has also been indicated together with the prolongation of the P300 latency as a potential indicator differentiating AD and mild cognitive impairment from healthy controls.^120^ In the case of P300 latency, a statistically significant prolongation was found in HT patients compared to controls. However, the obtained results were heterogeneous, and none of the controlled variables were statistically significant moderators. Further analyses showed a decrease in the P300 amplitude in HT patients, and this result was homogeneous. Similar results were obtained, among others, in patients with tinnitus,^121^ but the results of only four studies (11 comparisons) were used in the current meta‐analysis. The last part of the current review focused on the results of MRI, fMRI, DTI, PET, MRS, and SPECT studies. The studies included in the review were characterized by very diverse methodology, which made their further quantitative analysis impossible. Most of them presented both structural (including the right hippocampus or cerebellum) and functional (WM, parietal, and occipital lobes) differences; however, some studies indicated no differences between patients with HT and the control group, especially in drug‐naïve HT individuals. Due to such large differences, there is a need for further replications.

Our results confirm the occurrence of NCI in the group of patients with HT, but the profile of these dysfunctions seems to be very diverse and heterogeneous. In the case where the results of meta‐analyses showed statistically significant between‐group differences, these results were stable. However, the lack of between‐group differences was very sensitive to the presence of individual studies. Perhaps some explanation for this phenomenon could be the internal differentiation of impaired neurocognitive functions in the group of people with HT, which is dependent on both sociodemographic factors and clinical characteristics. In this case, it would be worth considering the use of person‐centered perspective methods in the calculations, such as Latent Profile Analysis, which would allow for the identification of specific subgroups in the studied populations. NCI seems to be conditioned not only by hormone levels, but also by other factors. In the case of HT patients, such predictors could be the severity of HT symptoms (especially fatigue) as well as psychosocial factors (such as the severity of depressive symptoms), which, however, were not controlled to a sufficient degree in the studies included in the current systematic review and meta‐analysis. This may be indicated by the difficulties that persist in some patients despite LT4 supplementation. Previous NCI models in this clinical group have pointed, among other things, to the role of thyroid hormones in CNS development throughout life, which influence, among other things, myelinating processes, cell migration and differentiation; promote or limit neurotransmitter release^122^; participate in free radical production,^123^ as well as, for example, the relationship between HT and brain serotonin reduction,^124^ although it should be noted that much of these data come from animal studies. The review observed a number of correlations between CNS functions and hormone levels, but causality cannot be inferred in this case. In addition to the biological mechanisms associated with thyroid hormones, a number of other factors should also be taken into account, such as comorbidity, for example, metabolic syndrome,^125^ dyslipidemia,^126^ and hypertension.^127^ In studies conducted on older populations, it can be very difficult to isolate the effect of HT from the potential impact of other comorbidities on NCI. In the case of AD, previous collective analyses do not clearly indicate a significant role for HT. The results of a meta‐analysis presented by Salehipour et al.^128^ showed that HT was significantly more prevalent in AD compared with controls, whereas Dolatshahi et al.^111^ noted that serum total and fT3 and CSF total T3 levels are significantly lower in AD patients compared to controls—however, in the case of TSH, total T4, and fT4, there were no differences between the groups. A study conducted by Brenowitz et al.^129^ indicated that HT was associated with severe atherosclerosis but not AD pathologies. Treatment also remains an open question. Although it was not possible to analyze treatment duration and LT4 doses as potential moderators in the review, the data indirectly indicate that it may play an important role in NCI. Some comparative studies have shown no differences in selected neuropsychological tests between individuals with HT and controls, and no structural differences in the CNS were observed between controls and individuals with HT on stable treatment.^46^, ^47^ However, such findings should not be treated as confirmation of a beneficial effect, but should be a basis for more in‐depth analysis. The potential impact of treatment on neurocognitive functioning should be assessed in a collective analysis of randomized trials and/or high‐quality prospective intervention studies. The role of selected psychological factors, such as the severity of depressive symptoms, also requires further analysis. The analyses conducted did not allow for determining whether they are moderators, and the results of the identified studies are ambiguous in this regard. It can be assumed that mood disorders, which are often observed in patients with HT, may be both a predictor and an effect of NCI. Therefore, in further analyses, it is also worth paying attention to the effects of cognitive decline in this clinical group, collectively analyzing their significance for, among others, quality of life, mood, adherence to recommended treatment, and social functioning of people with HT.

The quality of the vast majority of studies included in the systematic review was satisfactory. Potential limitations also did not appear to have a significant impact on the findings—including study quality as a moderator did not have a statistically significant effect on the meta‐analysis results. On the other hand, the reliability of our results may be reduced by publication bias, which we observed in some of our findings. Visual inspection of the funnel plots included in the Supplementary Files indicates this type of bias in the case of, among others, the prevalence of NCI assessed with MMSE, as well as correlations between TSH and MMSE. For this reason, some of the results should be interpreted with great caution. In our calculations, we also decided to perform meta‐regression instead of subgroup analyses. In the case of continuous variables such as age, this approach seems more precise than division into subgroups.

The results of the systematic review are not consistent with the results of other systematic reviews,^16^, ^17^ and individual participant data analysis,^18^ which indicated a lack of association between HT and SCH and NCI. These studies mostly analyzed odds/risk ratios in studies conducted on large populations of people 60+. Some cohort studies (e.g., Park et al.^76^) used the lack of a documented diagnosis of thyroid disease as an inclusion criterion. The results presented in our systematic review, covering case–control designs and analyses mostly conducted on continuous data are completely different and emphasize how serious a problem NCI is in the HT group.

The current review is not without limitations. A potential limitation may be the heterogeneity of the causes of HT (such as Hashimoto's and goiters), as well as its severity, including combined analyses of HT and SCH. However, in our work we decided to take a combined approach to these health problems, and to assess its association with NCI independently of its etiopathogenesis. This approach also allowed for the analysis of hormone levels as potential moderators, but they were treated as continuous rather than dichotomous variables. In most analyses, the number of included studies was fewer than 10, so the meta‐regression results should be interpreted with caution. Differences in hormone reference ranges were also found—despite controlling for this variable as a moderator, it may have contributed to sampling bias. It should be noted that the very large variability in the way the study results were reported, including the omission of reporting means and SDs, contributes to the limited possibility of further meta‐analyses. In addition, the omission of information on important characteristics, such as apolipoprotein E (*APOE*) genes, treatment time/time since diagnosis, levothyroxine doses, and others, reduces the possibility of analyzing potential factors determining the differences between studies. So, the data presented should also not be interpreted as a potential effect of treatment. Meta‐regressions using treatment indicators do not suggest that this variable is a statistically significant moderator, but due to numerous missing data it was impossible to correctly determine the role of this factor. Such conclusions can only be drawn on the basis of meta‐analyses of randomized controlled trials, in which special attention is paid to the risk of bias. Regarding NCI severity analyses, not all studies were comparable in terms of gender, age, and education (e.g., Djurovic et al.^42^), and that in some studies the inclusion criterion was the stated presence of NCI in the HT group (e.g., Miller et al.^71^). Some studies also included participants who did not have any known thyroid diseases, but laboratory tests results indicated SCH (e.g., Park et al.^76^), or excluded subjects with a previous diagnosis of thyroid dysfunction (Resta et al.^48^). Some studies also excluded patients with NCI, for example, Osterweil et al.^33^ or Yuan et al.^51^ Despite the relatively large number of studies identified in the systematic review, it was possible to analyze only a few of the neuropsychological tools in more detail. Caution should also be exercised with the results, as HT treatment, as well as high levels of thyroid hormones in the blood, can lead to low detected TSH. In the case of neuroimaging studies, the logic grid did not use terms related to neuroimaging, including, for example, MRI or P300, so there is a high probability that not all articles on the above topics were identified in the search. We also decided to conduct a meta‐analysis of the N200 peak latency and P300 amplitude despite the fact that they were reported in only three and four studies, respectively. These results should be treated with special caution, in particular the results of the N100 peak meta‐regression, which may falsely indicate potential moderators. Furthermore, due to the limited number of sources and the use of, among others, different neuropsychological tasks during neuroimaging, only a qualitative description was possible. In our review, we also omitted a review of study registries, as we focused on results that could then be subjected to collective analysis. In addition, in the case of many cross‐sectional studies, which were the source of the vast majority of the results presented in the article, pre‐registration was often not performed, which means that the potential results of such a review could be source of bias.

With regard to pre‐registration, a change has been made in the current version of the systematic review that should be noted: the quality of the studies included in the review was assessed using tools developed by the Joanna Briggs Institute (JBI). This change was based on the assessment that this set of tools would better allow for the determination of study quality and was agreed upon by consensus among the authors of the paper. Further works could also include non‐English publications and gray literature (pre‐prints, dissertations, and so forth) in order to reduce publication bias.

## 5 CONCLUSIONS

Pooled analyses of the results obtained using the MMSE indicate that NCI is a fairly common clinical problem in the population of HT patients, affecting about 30% of this clinical group, whereas TSH level was negatively associated with results obtained with this tool. Meta‐analyses indicate the occurrence of a statistically significant deterioration in neurocognitive functioning in HT patients, assessed using the MMSE and the WMS. In the case of TMT, DST, and fluency, the results were ambiguous and require more data. Meta‐regression showed that statistically significant moderators were female ratio (TMT B), mean sample age (TMT A), TSH levels (TMT A), fT4 values (DST backward), and fT3 (MMSE). In the case of auditory potentials, statistically significant differences between HT patients and controls were observed in the latencies of the N200 and P300 waves, as well as in the P300 amplitude. In the case of the remaining results obtained with neuroimaging methods, only qualitative analysis was possible, which did not allow for drawing clear conclusions.

## AUTHOR CONTRIBUTIONS

**Daniel Pankowski**: Conceptualization; methodology; formal analysis; investigation (lead); resources, data curation; writing—original draft; writing—review & editing (lead); visualization; supervision, project administration. **Kinga Wytrychiewicz‐Pankowska**: Investigation (supporting); writing—review & editing (supporting).

## CONFLICT OF INTEREST STATEMENT

Daniel Pankowski has no competing interests to disclose. Kinga Wytrychiewicz‐Pankowska has no competing interests to disclose. Any author disclosures are available in the Supporting Information.

## Supporting information



Supporting Information

Supporting Information

Supporting Information

Supporting Information

Supporting Information

Supporting Information

Supporting Information

Supporting Information

Supporting Information

Supporting Information

Supporting Information

## Data Availability

Data used for purposes of this work will be available on the registration page: PROSPERO, CRD42023451054.
