# Supplementary material for: Prevalence, hormonal correlates, severity, and neural basis of neurocognitive impairment in patients with hypothyroidism: Systematic review and meta‐analyses
Source: Alzheimers Dement. 2025 Nov 26;21(11):e70924. doi: 10.1002/alz.70924 (PMC12657124; doi:10.1002/alz.70924)
Supplement: Supplementary file 2 — Supporting Information [file ALZ-21-e70924-s007.docx]

Supplementary Table 1. Quality appraisal

| Prevalence | | | | | | | | | | |
| --- | --- | --- | --- | --- | --- | --- | --- | --- | --- | --- |
| Studies | 1. Were the criteria for inclusion in the sample clearly defined | 2. Were the study subjects and the setting described in detail? | 3. Was the exposure measured in a valid and reliable way | 4. Were objective, standard criteria used for measurement of the condition | 5. Were confounding factors identified * | 6. Were strategies to deal with confounding factors stated * | 7. Were the outcomes measured in a valid and reliable way | 8. Was appropriate statistical analysis used |  |  |
| Bajaj et al., 2014 | Yes | Yes | Yes | Yes | NA | NA | Yes | Yes |  |  |
| Ganguli et al., 1996 | Yes | Yes | Yes | Yes | NA | NA | Yes | Yes |  |  |
| Jaiswal et al., 2016 | Yes | Yes | Yes | Yes | NA | NA | Yes | Yes |  |  |
| Jessy et al., 2024 | Yes | Yes | Yes | Yes | NA | NA | Yes | Yes |  |  |
| Kalra et al., 2020 | Yes | Yes | Yes | Yes | NA | NA | Yes | Yes |  |  |
| Kamyshna et al., 2022 | Yes | Yes | Yes | Yes | NA | NA | Yes | Yes |  |  |
| Kaur et al., 2021 | Yes | Yes | Yes | Yes | NA | NA | Yes | Yes |  |  |
| Kramer et al., 2009 | Yes | Yes | Yes | Yes | NA | NA | Yes | Yes |  |  |
| Lesiv, 2020 | Unclear | Yes | Yes | Yes | NA | NA | Yes | Yes |  |  |
| Maugeri et al., 1998 | No | Yes | Yes | Yes | NA | NA | Yes | Yes |  |  |
| Miulescu et al., 2018 | No | Yes | Yes | Yes | NA | NA | Yes | Yes |  |  |
| Mulat et al., 2021 | Yes | Yes | Yes | Yes | NA | NA | Yes | Yes |  |  |
| Osterweil et al., 1992. | Yes | Yes | Yes | Yes | NA | NA | Yes | Yes |  |  |
| Parsaik et al., 2014 | Yes | Yes | Yes | Yes | NA | NA | Yes | Yes |  |  |
| Poojary et al., 2023 | Yes | No | Yes | Yes | NA | NA | Yes | Yes |  |  |
| Su et al., 2023 | Yes | Yes | Yes | Yes | NA | NA | Yes | Yes |  |  |
| Wekking et al., 2005 | Yes | Yes | Yes | Yes | NA | NA | Yes | Yes |  |  |
| Xu et al., 2018 | Yes | Yes | Yes | Yes | NA | NA | Yes | Yes |  |  |
| Correlations | | | | | | | | | | |
| Aghili et al., 2012 | Yes | Yes | Yes | Yes | NA | NA | Yes | Yes |  |  |
| Correia et al., 2009 | Yes | Yes | Yes | Yes | NA | NA | Yes | Yes |  |  |
| Djurovic et al., 2018 | Yes | Yes | Yes | Yes | NA | NA | Yes | Yes |  |  |
| Goyal et al., 2020 | Yes | No | Yes | Yes | NA | NA | Yes | Yes |  |  |
| Jaiswal et al., 2016 | Yes | Yes | Yes | Yes | NA | NA | Yes | Yes |  |  |
| Kamyshna et al., 2022 | Yes | Yes | Yes | Yes | NA | NA | Yes | Yes |  |  |
| Krausz et al., 2004 | Yes | Yes | Yes | Yes | NA | NA | Yes | Yes |  |  |
| Kumar et al., 2018 | Yes | Yes | Yes | Yes | NA | NA | Yes | Yes |  |  |
| Leyhe et al., 2013 | Yes | Yes | Yes | Yes | NA | NA | Yes | Yes |  |  |
| Menicucci et al., 2013 | Yes | Yes | Yes | Yes | NA | NA | Yes | Yes |  |  |
| Miulescu et al., 2018 | No | Yes | Yes | Yes | NA | NA | Yes | Yes |  |  |
| Osterweil et al., 1992 | Yes | Yes | Yes | Yes | NA | NA | Yes | Yes |  |  |
| Quinque et al., 2014 | No | Yes | Yes | Yes | NA | NA | Yes | Yes |  |  |
| Resta et al., 2012 | Yes | No | Yes | Yes | NA | NA | Yes | Yes |  |  |
| Samuels et al., 2016 | Unclear | Yes | Yes | Yes | NA | NA | Yes | Yes |  |  |
| Yin et al., 2021 | Yes | Yes | Yes | Yes | NA | NA | Yes | Yes |  |  |
| Yuan et al., 2020 | Yes | Yes | Yes | Yes | NA | NA | Yes | Yes |  |  |
| Severity | | | | | | | | | | |
|  | 1. Were the groups comparable other than presence of disease in cases or absence of disease in controls? ** | 2. Were cases and controls matched appropriately | 3. Were the same criteria used for identification of cases and controls | 4. Was exposure measured in a standard, valid and reliable way | 5. Was exposure measured in the same way for cases and controls | 6. Were confounding factors identified ** | 7. Were strategies to deal with confounding factors stated ** | 8. Were outcomes assessed in a standard, valid and reliable way for cases and controls | 9. Was the exposure period of interest long enough to be meaningful | 10. Was appropriate statistical analysis used |
| Almeida et al., 2007 | Yes | Yes | Yes | Yes | Yes | NA | NA | Yes | NA | Unclear |
| Bajaj et al., 2014 | Yes | Yes | Yes | Yes | Yes | NA | NA | Yes | NA | Yes |
| Baldini et al., 1997 | Unclear | Yes | Yes | Yes | Yes | NA | NA | Yes | NA | Yes |
| Bocheva et al., 2022 | Unclear | Unclear | Yes | Yes | Yes | NA | NA | Unclear | NA | Yes |
| Cao et al., 2023 | No | Yes | No | Yes | Yes | NA | NA | Yes | NA | Yes |
| Ceresini et al., 2009 | Yes | Yes | Yes | Yes | Yes | NA | NA | Yes | NA | Yes |
| Constant et al., 2005 | No | Yes | Yes | Yes | Yes | NA | NA | Yes | NA | Yes |
| Correia et al., 2009 | Yes | Unclear | Yes | Yes | Yes | NA | NA | Yes | NA | Yes |
| Djurovic et al., 2018 | No | Yes | Yes | Yes | Yes | NA | NA | Yes | NA | Yes |
| Ettleson et al., 2024 | Yes | Yes | Yes | Yes | Yes | NA | NA | Yes | NA | Yes |
| Formiga et al., 2014 | Unclear | Yes | Yes | Yes | Yes | NA | NA | Yes | NA | Yes |
| Goyal et al., 2020 | Unclear | Unclear | Yes | Yes | Yes | NA | NA | Yes | NA | Yes |
| He et al., 2011 | Yes | Unclear | Yes | Yes | Yes | NA | NA | Yes | NA | Yes |
| Hu et al., 2016 | Yes | Yes | No | Yes | Yes | NA | NA | Yes | NA | Yes |
| Jaiswal et al., 2016 | Yes | Yes | Yes | Yes | Yes | NA | NA | Yes | NA | Yes |
| Jhandi et al., 2024 | Yes | Yes | Unclear | Unclear | Unclear | NA | NA | Yes | NA | Yes |
| Kalra et al., 2020 | Yes | Unclear | Yes | Yes | Yes | NA | NA | Yes | NA | Yes |
| Kamyshna et al., 2022 | Unclear | Unclear | Yes | Yes | Yes | NA | NA | Yes | NA | Yes |
| Kaur et al., 2021 | Yes | Yes | Yes | Yes | Yes | NA | NA | Yes | NA | Yes |
| Khorasani et al., 2019 | Yes | Yes | Yes | Yes | Yes | NA | NA | Yes | NA | Yes |
| Kramer et al., 2009 | No | Yes | Yes | Yes | Yes | NA | NA | Yes | NA | Yes |
| Kumar et al., 2018 | Unclear | Yes | Yes | Yes | Yes | NA | NA | Yes | NA | Yes |
| Kumar et al., 2025 | Yes | Yes | Yes | Yes | Yes | NA | NA | Yes | NA | Yes |
| Lesiv, 2020 | Unclear | Unclear | Yes | Yes | Yes | NA | NA | Yes | NA | Yes |
| Lesiv et al., 2021 | Unclear | Unclear | Yes | Yes | Yes | NA | NA | Yes | NA | Yes |
| Leyhe et al., 2008 | Yes | Yes | Yes | Yes | Yes | NA | NA | Yes | NA | Yes |
| Leyhe et al., 2013 | Yes | Yes | Yes | Yes | Yes | NA | NA | Yes | NA | Yes |
| Liu et al., 2020 | Yes | Yes | Yes | Yes | Yes | NA | NA | Yes | NA | Yes |
| Menicucci et al., 2013 | Unclear | Yes | Yes | Yes | Yes | NA | NA | Yes | NA | Yes |
| Miller et al., 2006 | Yes | Yes | Yes | Yes | Yes | NA | NA | Yes | NA | Yes |
| Miller et al., 2007 | Yes | Yes | Yes | Yes | Yes | NA | NA | Yes | NA | Yes |
| Mishra et al., 2016 | Yes | Unclear | Yes | Yes | Yes | NA | NA | Yes | NA | Yes |
| Mishra et al., 2018 | Yes | Unclear | Yes | Yes | Yes | NA | NA | Yes | NA | Yes |
| Monzanil et al., 1993 | Unclear | Unclear | Yes | Yes | Yes | NA | NA | Yes | NA | Yes |
| Oerbeck et al., 2005 | Unclear | Yes | Yes | Yes | Yes | NA | NA | Yes | NA | Yes |
| Osterweil et al., 1992 | Yes | Yes | Yes | Yes | Yes | NA | NA | Yes | NA | Yes |
| Pandey et al., 2017 | Yes | Unclear | Yes | Yes | Yes | NA | NA | Yes | NA | Yes |
| Park et al., 2010 | Unclear | Yes | Yes | Yes | Yes | NA | NA | Yes | NA | Yes |
| Quinque et al., 2014 | Unclear | Yes | Yes | Yes | Yes | NA | NA | Yes | NA | Yes |
| Resta et al., 2012 | Unclear | Yes | Yes | Yes | Yes | NA | NA | Yes | NA | Yes |
| Samuels et al., 2007 | Yes | Yes | Yes | Yes | Yes | NA | NA | Yes | NA | Yes |
| Schraml et al., 2011 | Yes | Unclear | Yes | Yes | Yes | NA | NA | Yes | NA | Yes |
| Sheng et al., 2024 | Yes | Unclear | Yes | Yes | Yes | NA | NA | Yes | NA | Yes |
| Singh et al., 2014 | Unclear | Yes | Yes | Yes | Yes | NA | NA | Yes | NA | Yes |
| Su et al., 2023 | Yes | Yes | Yes | Yes | Yes | NA | NA | Yes | NA | Yes |
| Wijsman et al., 2013 | No | Yes | Yes | Yes | Yes | NA | NA | Yes | NA | Yes |
| Yamamoto et al., 2012 | Unclear | Yes | Yes | Yes | Yes | NA | NA | Yes | NA | Yes |
| Yin et al., 2013 | Yes | Yes | Yes | Yes | Yes | NA | NA | Yes | NA | Yes |
| Yin et al., 2021 | Yes | Yes | Yes | Yes | Yes | NA | NA | Yes | NA | Yes |
| Yuan et al., 2020 | Unclear | Unclear | Yes | Yes | Yes | NA | NA | Yes | NA | Yes |
| Zhu et al., 2006 | Unclear | Unclear | Yes | Yes | Yes | NA | NA | Yes | NA | Yes |
| Electromyography, evoked potentials and electroencephalography | | | | | | | | | | |
| Anjana et al., 2006 | Unclear | Unclear | Yes | Yes | Yes | NA | NA | Yes | NA | Yes |
| Anjana et al., 2008 | Yes | Unclear | Yes | Yes | Yes | NA | NA | Yes | NA | Yes |
| Jaiswal et al., 2016 | Yes | Yes | Yes | Yes | Yes | NA | NA | Yes | NA | Yes |
| Jensovsky et al., 2002 | Unclear | Unclear | Unclear | Yes | Unclear | NA | NA | Yes | NA | Yes |
| Kakked et al., 2013 | Unclear | Unclear | Yes | Yes | Yes | NA | NA | Yes | NA | Yes |
| Menicucci et al., 2013 | Unclear | Yes | Yes | Yes | Yes | NA | NA | Yes | NA | Yes |
| Mishra et al., 2016 | Yes | Unclear | Yes | Yes | Yes | NA | NA | Yes | NA | Yes |
| Mishra et al., 2018 | Yes | Unclear | Yes | Yes | Yes | NA | NA | Yes | NA | Yes |
| Nazliel et al., 2002 | Unclear | Unclear | Yes | Yes | Yes | NA | NA | Yes | NA | Yes |
| Oerbeck et al., 2007 | Unclear | Yes | Yes | Yes | Yes | NA | NA | Yes | NA | Yes |
| Osterweil et al., 1992 | Yes | Yes | Yes | Yes | Yes | NA | NA | Yes | NA | Yes |
| Ozata et al., 1997 | Yes | Unclear | Yes | Yes | Yes | NA | NA | Yes | NA | Yes |
| Paladugu et al., 2015 | Unclear | Unclear | Yes | Yes | Yes | NA | NA | Yes | NA | Yes |
| Rizzo et al., 2008 | Unclear | Yes | Yes | Yes | Yes | NA | NA | Yes | NA | Yes |
| Sharma et al., 2014 | Unclear | Unclear | Unclear | Yes | Yes | NA | NA | Yes | NA | Yes |
| Waliszewska-Prosół et al., 2021 | Yes | Unclear | Yes | Yes | Yes | NA | NA | Yes | NA | Yes |
| MRI, fMRI, SPECT, MRS, PET, and DTI | | | | | | | | | | |
| Bauer et al., 2009 | Yes | Yes | Yes | Yes | Yes | NA | NA | Yes | NA | Yes |
| Bladowska et al., 2019 | Unclear | Unclear | Yes | Yes | Yes | NA | NA | Yes | NA | Yes |
| Cao et al., 2023 | No | Yes | No | Yes | Yes | NA | NA | Yes | NA | Yes |
| Chambers et al., 2021*** | NA | NA | NA | NA | NA | NA | NA | NA | NA | NA |
| Cooke et al., 2014 | Yes | Yes | Yes | Yes | Yes | NA | NA | Yes | NA | Yes |
| Gunbey et al., 2021 | Unclear | Unclear | Yes | Yes | Yes | NA | NA | Yes | NA | Yes |
| He et al., 2011 | Yes | Unclear | Yes | Yes | Yes | NA | NA | Yes | NA | Yes |
| Kaya et al., 2007 | Unclear | Unclear | Yes | Yes | Yes | NA | NA | Yes | NA | Yes |
| Krausz et al., 2004 | Unclear | Unclear | Yes | Yes | Yes | NA | NA | Yes | NA | Yes |
| Kumar et al., 2018 | Unclear | Yes | Yes | Yes | Yes | NA | NA | Yes | NA | Yes |
| Kumar et al., 2025 | Yes | Yes | Yes | Yes | Yes | NA | NA | Yes | NA | Yes |
| Leyhe et al., 2013 | Yes | Yes | Yes | Yes | Yes | NA | NA | Yes | NA | Yes |
| Liu et al., 2020 | Yes | Yes | Yes | Yes | Yes | NA | NA | Yes | NA | Yes |
| Nagamachi et al., 2004 | Unclear | Unclear | Yes | Yes | Yes | NA | NA | Yes | NA | Yes |
| Quinque et al., 2014 | Unclear | Yes | Yes | Yes | Yes | NA | NA | Yes | NA | Yes |
| Singh et al., 2013 | Unclear | Unclear | Yes | Yes | Yes | NA | NA | Yes | NA | Yes |
| Singh et al., 2014 | Unclear | Yes | Yes | Yes | Yes | NA | NA | Yes | NA | Yes |
| Singh et al., 2016 | Unclear | Yes | Yes | Yes | Yes | NA | NA | Yes | NA | Yes |
| Su et al., 2023 | Yes | Yes | Yes | Yes | Yes | NA | NA | Yes | NA | Yes |
| Waliszewska-Prosół et al., 2021 | Yes | Unclear | Yes | Yes | Yes | NA | NA | Yes | NA | Yes |
| Wu et al., 2021 | Yes | Yes | Yes | Yes | Yes | NA | NA | Yes | NA | Yes |
| Yin et al., 2013 | Yes | Yes | Yes | Yes | Yes | NA | NA | Yes | NA | Yes |
| Yin et al., 2021 | Yes | Yes | Yes | Yes | Yes | NA | NA | Yes | NA | Yes |
| Zhang et al., 2015 | Unclear | Unclear | Yes | Yes | Yes | NA | NA | Yes | NA | Yes |
| Zhu et al., 2006 | Unclear | Unclear | Yes | Yes | Yes | NA | NA | Yes | NA | Yes |

MRI - Magnetic resonance imaging, fMRI - Functional magnetic resonance imaging, SPECT - Single photon emission computed tomography, MRS - Magnetic resonance spectroscopy, PET - Positron emission tomography, DTI - Diffusion tensor imaging

* - In the quality assessment of prevalence and correlation studies, confounders were not assessed. The outcome of interest was the percentage of patients with NCI/correlation coefficients, regardless of other variables that might affect it. This strategy was adopted because such an outcome would have the greatest clinical utility. Potential confounders were intended to be assessed as moderators in meta-regression.

** - In pooled analyses, we wanted to use means and standard deviations, without using various correction methods and other statistical operations used to deal with confounding factors. In such a situation, it was crucial for us to match the study participants with respect to sex, age, and education as the most important sociodemographic factors relevant to group comparability.

*** - Analyses were performed based on data from Biobank - no purposive selection from the general/clinical population was used
