# Supplementary material for: Prevalence, hormonal correlates, severity, and neural basis of neurocognitive impairment in patients with hypothyroidism: Systematic review and meta‐analyses
Source: Alzheimers Dement. 2025 Nov 26;21(11):e70924. doi: 10.1002/alz.70924 (PMC12657124; doi:10.1002/alz.70924)
Supplement: Supplementary file 3 — Supporting Information [file ALZ-21-e70924-s004.docx]

Supplementary Table 2. Prevalence of neurocognitive impairment in hypothyroid patients

| Authors | Country | Sample size | Basic sociodemographic data | Clinical data | Ranges tsh | Neurocognitive tests | Definition of neurocognitive impairment | Main findings |
| --- | --- | --- | --- | --- | --- | --- | --- | --- |
| Bajaj et al., 2014 [25] | India | 103 patients diagnosed with SCH | 60.19% females; mean age: 75.74 ± 9.37; NI about mean time of education | NI | TSH concentration. >5.5 mIU/liter with serum‑free T4 and T3 concentration being within the reference range. | MMSE and CDT | MMSE ≤ 24 | NCI (by MMSE) was found in 32.03% |
| Ganguli et al., 1996 [22] | USA | 28 participants aged 65 and older with TSH above 5.5 | 67.9% females; mean age: 79.2(6.1); NI about mean education time | NI | TSH: 0.3-5.5 mU/L (n = 66); 0.5-10.0 mU/L (n =1); 0.3-6.3 mU/L (n = 56); 0.0-8.0 mU/L (n = 13); and 0.27-5.39 mU/L (n = 58) | CDR | Possibly demented (CDR = 0.5), and definitely demented (CDR higher or =1) | 68.4% had CDR scores 0.5 and higher |
| Jaiswal et al., 2016 [24] | India | 36 adult SCH patients | 86.1% females; mean age: 35.5±5.9; NI about mean education time | Mean TSH (μIU/ml) 7.2±2.5; mean FT3 (pg/ml) 2.7±0.7; mean FT4 (ng/dl) 1.1±1.0; mean BMI: 26.3±3.5 | TSH: 0.34–4.24 μIU/ml; FT3: 2.0–4.2 pg/ml; FT4: 0.6–1.7 ng/dl | ABCT and DST | 95 percentile standard score value of 124 was set as cutoff for memory difficulties | DST: 95,2% SCH patients had NCI |
| Jessy et al., 2024 [26] | India | 209 HT patients (13 overt HT and 196 SCH) | 55.5% females; mean age: 63; mean education time: 15 yeards | NI | TSH between 0.54 and 5.30 mIU/mL and T4 between 5.1 and 14.1 μg/dL | CDR; MMSE; ACE | CDR score of 0.5 was used to classify participants with mild cognitive impairment | CDR: 13.9% |
| Kamyshna et al., 2022 [23] | Ukraine | 16 patients with postoperative HT; 65 patients with HT AIT, and 72 patients with both AIT and elevated anti-Tg and anti-TPO. | Postoperative HT: 100% females; mean age: 47.30±12.27; AIT: 100% females; mean age: 46.72±15.49; AIT and elevated anti-Tg and anti-TPO: 100% females; mean age: 45.02±13.65; NI about mean education times | Postoperative HT: mean TSH (mIU/mL): 8.61±0.84; mean fT4 (pmol/L): 3.44±0.31; AIT: mean TSH (mIU/mL): 7.09±0.50; mean fT4 (pmol/L): 4.13±0.52; AIT and elevated anti-Tg and anti-TPO: mean TSH (mIU/mL): 2.38±0.62; mean fT4 (pmol/L): 8.51±0.82. NI about mean fT3 and BMI | TSH: 0.3–4.0 mIU/mL; fT4: 6.0–13.0 pmol/L for males and 7.0–13.5 pmol/L for females; anti-TPO:0–30 IU/mL; anti-TG: 0–65 IU/mL | MMSE | NI | 50% of patients with Postoperative HT had NCI according to the MMSE test, 50,8% patients with AIT with HT and 16.7% in the group of AIT patients |
| Kalra et al., 2020 [21] | India | 39 patients diagnosed with SCH | 92,3% females; mean age: 30.3 ± 7.7; NI about mean education time | TSH (mIU/L) 6.36 ± 1.31; NI about meean fT3, fT4, BMI | TSH below 4.5 mIU/L | MMSE; DSST, DVT, animal naming test, N-back test, ToL, ST, WCST, AVLT, ROCFT. | NI | The category fluency: 35.9%; visual memory delayed recall: 48.71%; Visual memory immediate recall: 38.5%; Auditory verbal learning and memory: 33.33%; Auditory verbal learning and memory (delayed recall): 38.5%; Auditory verbal learning and memory (immediate recall): 25.6%; Working memory: 25.6%; Planning: 0%; Response inhibition: 17.9%; Set shifting: 7.6%; Visuospatial construction: 5.1%; Attention: 5.1%; Word recognition: 20.5%; Mental speed: 5.1% |
| Kaur et al., 2021 [27] | India | 100 patients with SCH | 69% females, mean age: 68.23 ± 6.95; NI about mean education time | mean TSH (mIU/L): 6.9 ± 2.72; mean fT3 (pM/L): 4.69 ± 1.24; mean fT4 (pM/L): 16.52 ± 2.99; mean BMI: 25.47 ± 2.33 | TSH: 0.27 - 4.0 μIU/mL; fT3: 1.3 - 3.1 nmol/L; fT4: 66 - 181 nmol/L | MMSE, CDT | MMSE≤24; CDT: a score of ≥ 3 | MMSE: NCI was present in 27%; CDT: NCI was present in 34%. |
| Kramer et al., 2009 [28] | USA | 149 HT participants with a positive history of physician-diagnosed primary HT who were currently using LT4 | 81.6% females; mean age: 76.1(9.6); NI about mean education time | mean TSH (mIU/l): 1.54 (1.59); mean BMI: 27.4 (5.5); NI about mean fT3 and fT4 | TSH: 0.49–4.67 mIU/l) | 3MSE; TMT- B, and verbal fluency | MMSE score lower than 78 (3MSE) | NCI was present in 5.6% |
| Lesiv, 2020 [29] | Ukraine | 18 patients with HT who received LT4 at a dose of 100-150 mg | HT patients: NI about % of females; mean age: 49.24±0.83, NI about mean education time. | HT patients: mean TSH, mIU/L: 3.16±0.79. NI about mean fT3, fT4, BMI | TSH: <4.4 mIU/L | MMSE, ACE-R | NI | MMSE: 10.3% ( patients with HT); ACE-R: 9.6% (patients with HT) |
| Maugeri et al., 1998 [30] | Italy | 10 patients with HT | NI | T3 (nglml): 0.6 + 0.2; T4 (nglml): 36.0 + 12.0; TSH (IUlml): 9.2 + 3.0 | T3 (nglml): 0.8 - 2.2; T4 (nglml): 50.0 - 120; TSH (IUlml): 0.2 - 3.0 | MMSE | MMSE≤24 | HT: 20% |
| Miulescu et al., 2018 [31] | Hungary | 12 patients with overt HT and type 2 diabetes mellitus | 91,7% females; mean age: 64.92±5.84; NI about mean time of education | mean TSH (mIU/L) 11.76±4.43; mean FT4 (ng/dL) 0.53±0.08; NI about mean fT3 and BMI | TSH: 0.4-4.5 mIU/L; fT4: 0.7-1.8 ng/dL). | MMSE | MMSE≤24 | HT: 25.% |
| Mulat et al., 2021 [32] | Ethiopia | 216 adult HT patients | 73.1% females; mean age: 42.5 (± 11); NI about mean time of education | mean TSH: 6.98 ± 6.8 (mIU/L); NI about mean fT3, fT4, BMI | TSH level > 4.5 mIU/L, FT4 < 10.3 pmol/I or FT3 < 2.3 pmol/diagnosed as HT | MMSE | MMSE score below 22 (no or primary level education), less than 24 ( secondary level education), less than 25 (diploma and above educational status) | According to MMSE score the prevalence of NCI was 27.3% |
| Osterweil et al., 1992 [33] | USA | 54 non-demented HT patients | 46% females; mean age 68.6 (16.4); mean education time: 11.2 (3.6) | mean TSH 66.3 (55.4)pu/mL; mean T3: 68.5 (41.2)ng/dL; mean T4 2.3 (2.2.); NI about mean BMI | TSH: 0.3-5.7 pu/mL; T4 4.2-11.0 pg/dL; T3 70-160 ng/dL | MMSE; Copying the Cube; IPALT; Animal Naming; DST (WAIS); SDMT, TMT; The Language Disorder tests were adapted from BDAE | MMSE<24 | The prevalence of NCI was 28% |
| Parsaik et al., 2014 [34] | USA | 141 patients with SCH and 313 patients with HT | 49.65% females (SCH), 70,29 (HT); median age: 81.67 (SCH), 81.20 (HT); median education time: 13 (both SCH and HT) | NI about mean TSH, fT3, fT$; mean BMI 27.16 (both SCH and HT) | NI | LM–II [delayed recall]; VR–II [delayed recall] from the WMS-R, AVLT; TMT-B and DSST from WAIS–R; BNT and Category Fluency Test, Picture Completion and Block Design from the WAIS-R | Mean score was ≥ 1.0 SD below the mean, when compared to normative data derived from Olmsted County; consensus agreement between evaluating physician, nurse and neuropsychologist after taking into account the other important information like education, occupation, visual impairment and deafness | 17.73% (SCH); 17.25% (HT) |
| Poojary et al., 2023 [35] | India | 163 diagnosed and under-treatment HT patients | NI about sample structure | NI | NI | Mini ACE | Normal cognition was operationalized as Mini ACE score > 25 | 26.38% |
| Su et al., 2023 [36] | China | 44 newly diagnosed patients with HT | 77.3% female; mean age 39.30 (9.80); mean education 13.57 (3.45) | median (IQR) TSH: 44.81 (55.51) mIU/L; mean fT3, pmol/L, 3.30 (1.18); mean fT4, pmol/L: 6.81 (2.22); NI about mean BMI | TSH: 0.35–4.94 mIU/L; fT4: 9.01–19.05 pmol/L | MMSE; MoCA | MoCA<26 | 36.4% HT patients |
| Wekking et al., 2005 [37] | Netherlands | 141 patients with primary hypothyroidism | 85.8% females; mean age: 47.8^9.8; NI about mean time of education | meadian TSH (mU/l): 1.4; mean free T4 (pmol/l): 14.8^2.9; mean T3 (nmol/l): 1.7^0.33; NI about mean BMI | NI | DSST; MCT; PASAT, DST (WAIS-III), CVLT; the Rivermead Behavioral Memory Test, Story Recall (Rivermead, stories) | For each method - scores in the reference groups | MCT paper and pencil tasks (%) 26.4%; MCT paper and pencil tasks (1 letter): 16.4%; PASAT: 14.9%; CVLT scores for immediate recall: 10.6%; the Rivermead stories immediate and delayed recall: 25%; the Rivermead stories relative delayed recall: 8.7% |
| Xu et al., 2018 [38] | China | 194 patients with Hashimoto thyroiditis | 18,6% females; mean age: 49.4 (9.8); median education time: 11 | mean TSH (IU/mL): 2.2 ± 0.9; mean FT3 (pmol/L): 4.8 ± 1.1; mean FT4 (pmol/L): 18.1 ± 4.4; mean BMI: 24.2 ± 3.2 | NI | MoCA | MoCA scores below 26 | 28.4%. |

ABCT - AB clock test; ACE-R - the Addenbrooke’s Cognitive Examination Scale; AIT - autoimmune thyroiditis; AVLT - Auditory Verbal Learning Test; BDAE - Boston Diagnostic Aphasia Exam; BMI – Body Mass Index; BNT - Boston Naming Test; CDR - Clinical Dementia Rating; CDT - Clock Drawing Test; CVLT - California Verbal Learning Test; DSST - Digit Symbol Substitution Test; DST - Digit Span Test; DVT - Digit Vigilance Test; FT3 – free triiodothyronine; FT4 – free thyroxine; HT – hypothyroidism; IPALT - Inglis Paired Associate Learning Test; LM - Logical Memory; LT4 – levothyroxine; MCT - The Memory Comparison Task; MMSE - Mini Mental State Examination; MoCA - Montreal Cognitive Assessment; NCI – neurocognitive impairment; NI – no information; ROCFT - Rey–Osterrieth Complex Figure Test; PASAT - Paced Auditory Serial Addition Task; SCH – subclinical hypothyroidism; SDMT - Symbol Digit Modalities Test; ST - Stroop Test; T3 – triiodothyronine; T4 – thyroxine; TSH - thyroid-stimulating hormone; TMT - Trail Making Test; ToL - Tower of London; VR - Visual Reproduction; WAIS - Wechsler Adult Intelligence Scale; WCST - Wisconsin card sorting test; WMS-R - Wechsler Memory Scale - Revised
