# Supplementary material for: Prevalence, hormonal correlates, severity, and neural basis of neurocognitive impairment in patients with hypothyroidism: Systematic review and meta‐analyses
Source: Alzheimers Dement. 2025 Nov 26;21(11):e70924. doi: 10.1002/alz.70924 (PMC12657124; doi:10.1002/alz.70924)
Supplement: Supplementary file 4 — Supporting Information [file ALZ-21-e70924-s008.docx]

Supplementary Table 3. Jackknife sensitivity analysis: PRs of neurocognitive impairment in people diagnosed with hypothyroidism

| Studies omitted | Proportion | 95% CI | *I^2^* | *Tau*^2^ | *Q* |
| --- | --- | --- | --- | --- | --- |
| All studies included | 0,291 | 0.258; 0.327 | 0 | 0 | 6.26 |
| Bajaj et al. (2014) | 0.286 | 0.250; 0.325 | 0 | 0 | 5.85 |
| Kamyshna, et al. (2022) | 0.275 | 0.238; 0.315 | 0 | 0 | 3.57 |
| Kaur et al. (2021) | 0.295 | 0.259; 0.334 | 0 | 0 | 5.93 |
| Lesiv (2020) | 0,296 | 0.262; 0.333 | 0 | 0 | 3.65 |
| Maugeri et al (1998) | 0,293 | 0.259; 0.329 | 0 | 0 | 5.84 |
| Miulescu et al (2018) | 0,292 | 0.259; 0.328 | 2,4% | 0 | 6.15 |
| Mulat et al (2021) | 0,30 | 0.259; 0.344 | 0 | 0 | 5.59 |
| Osterweil et al (1992) | 0.293 | 0.258; 0.330 | 3% | 0 | 6.18 |

*-p<0.05; **-p<0.01; ***-p<0.001
