# Supplementary material for: Prevalence, hormonal correlates, severity, and neural basis of neurocognitive impairment in patients with hypothyroidism: Systematic review and meta‐analyses
Source: Alzheimers Dement. 2025 Nov 26;21(11):e70924. doi: 10.1002/alz.70924 (PMC12657124; doi:10.1002/alz.70924)
Supplement: Supplementary file 5 — Supporting Information [file ALZ-21-e70924-s001.docx]

Supplementary Table 4. Correlations between cognitive tests scores and thyroid hormones

| Authors | Country | Sample size | Basic sociodemographic data | Clinical data | Hormone ranges | Cognitive tests | Main findings |
| --- | --- | --- | --- | --- | --- | --- | --- |
| Aghili et al., 2012 [40] | Iran | 60 patients with SCH | 85% females; mean age: 34 ±10; NI about mean education time | Baseline: mean TSH [ml/l] 8.29 ±4.9; mean FT4 [ng/dl] 1.38 ±0.26 | SCH was defined as serum TSH level between 4.5 mU/l and 10 mU/l in the presence of normal fT4 (0.8-2 ng/dl) | WMS | Significant negative corelation between TSH level and memory quotient |
| Correia et al., 2009 [41] | Ireland | 21 HT and 17 SCH | SCH: 94.1% females; mean age: 50.0±9.2; mean education time: 12.8±2.2; HT: 95,2% females; mean age: 44.0±10.9; mean education time: 12.5±3.1 | SCH: median TSH (mU/liter) 6.1; median FT4 (pmol/liter) 12.8; HT: median TSH (mU/liter) 38.9; median FT4 (pmol/liter): 7.6; NI about mean fT3 and BMI | NI | ROCFT; RBMT: Story Recall Subset; Face-Name Learning and Recall; Focused attention task; n-Back Task; ST | TSH levels correlated negatively with visuospatial construction ability, and visuospatial, verbal, associative, and working memory. |
| Djurovic et al., 2018 [42] | Serbia | 130 patients with HT on long-term LT4 replacement | Age group 20–49 (N=59): NI about % of females; mean age: 35.82±7.80; mean education time: 13.49±2.45; Age group 50 + (N=71): NI about % of females; mean age: 61.23±6.55; mean education time: 11.61±3.92 | Age group 20–49 (N=59): mean TSH (mIU/L) 3.64±2.74; mean FT3(pmol/L) 2.45±0.46; mean FT4 (pmol/L) 12.66±2.87; mean BMI: 24.91±3.67. Age group 50 + (N=71): mean TSH (mIU/L) 3.93±2.84; mean FT3(pmol/L) 2.33±0.49; mean FT4 (pmol/L) 12.11±3.15; mean BMI: 27.20±3.67 | TSH: 0.27–4.2 mIU/L; FT3: 2–4.25 ng/L; FT4: 7–18 ng/L; TPOAbs >130 U/mL, TgAbs >70 U/ml | MMSE, Verbal Span from WAIS and Visual Span from WMS-R, TMT, Phonemic fluency test | No statistically significant correlations between TSH, TgAb, TPOAb, FT4/FT3 and cognitive tests |
| Goyal et al., 2020 [43] | India | 30 drug‑naïve newly diagnosed HT patients | NI about females %; mean age: 31.6±8.4; NI about mean education time | Before treatment: TSH levels (μIu/mL): 26.43±10.24; fT3 (pg/ml): 0.30±0.09; fT4 (ng/dl): 0.27±0.10. | NI | ST | Positive correlation between reaction time of ST and serum TSH levels |
| Jaiswal et al., 2016 [24] | India | 36 adult SCH | 86.1% females; mean age: 35.5±5.9; NI about mean education time | Mean TSH (μIU/ml) 7.2±2.5; mean FT3 (pg/ml) 2.7±0.7; mean FT4 (ng/dl) 1.1±1.0; mean BMI: 26.3±3.5 | TSH: 0.34–4.24 μIU/ml; FT3: 2.0–4.2 pg/ml; FT4: 0.6–1.7 ng/dl | ABCT and DST | Serum TSH had significant negative correlation with DST score |
| Kamyshna et al., 2022 [23] | Ukraine | 16 with postoperative HT; 65 with HT (AIT), and 72 with both AIT and elevated anti-Tg and anti-TPO. | Postoperative HT: 100% females; mean age: 47.30±12.27; HT (AIT): 100% females; mean age: 46.72±15.49; AIT and elevated anti-Tg and anti-TPO: 100% females; mean age: 45.02±13.65; NI about mean education times | Postoperative hypothyroidism: mean TSH (mIU/mL): 8.61±0.84; mean fT4 (pmol/L): 3.44±0.31; HT (AIT): mean TSH (mIU/mL): 7.09±0.50; mean fT4 (pmol/L): 4.13±0.52; AIT and elevated anti-Tg and anti-TPO: mean TSH (mIU/mL): 2.38±0.62; mean fT4 (pmol/L): 8.51±0.82. NI about mean fT3 and BMI | TSH: 0.3–4.0 mIU/mL; fT4: 6.0–13.0 pmol/L for males and 7.0–13.5 pmol/L for females; anti-TPO:0–30 IU/mL; anti-TG: 0–65 IU/mL | MMSE | Positive correlation between NCI and fT4; negative correlation between the MMSE test and TSH in the blood |
| Krausz et al., 2004 [44] | Israel | 10 with newly diagnosed adult-onset HT due to atrophic or Hashimoto’s thyroiditis | 100% female; mean age, 45.9±15.1; NI about education | (Pretreatment) mean TSH: 15.1±2.9 mU/L,; mean free T4: 9.3±1.6 pmol/L, mean total T3: 2.4±1.0 nmol/L | NI | MMSE | Nonsignificant correlation between TSH and MMSE scores in HT patients before treatment |
| Kumar et al., 2018 [45] | India | 28 drug naive SCH | NI about participant's sex; mean age 31.53 ± 8.10; mean education 12.96 ± 3.08 | mean TSH 9.61 ± 1.39 μIU mL-1, NI about mean fT3 and fT4; mean BMI 24.71 ± 4.83 | NI | MMSE, PGIBBD | Partial correlations revealed that TSH was negatively correlated with immediate recall and MMSE |
| Leyhe et al., 2013 [46] | Germany | 18 Hashimoto thyroiditis patients on stable LT4 treatment, and a TSH concentration of 0.4–2.5 mU/l. | 84,6% females; mean age 43 ± 12; mean education 13 ± 4 | mean TSH (mU/l) 1.5 ± 1.0; mean fT4 (pmol/l) 15.5 ± 2.9; mean fT3 (pmol/l) 4.8 ± 0.6; mean BMI 25.8 ± 5.1 | TSH, 0.4–2.5 mU/l; free T4, 12–23 pmol/l; free T3, 3.5–6.5 pmol/l; TPOAbs,<100 IU/l; Tg-Abs,<100 IU/l. | d2 test | Statistically significant correlation between d2 test scores and TPO-Abs |
| Menicucci et al., 2013 [39] | Italy | 17 patients with SCH | 100% females; mean age, 28 ± 4; NI about mean education time | median TSH: 5.3; median fT3: 2.7; median fT4: 10.9; NI about mean BMI | TSH: 0.3-3.8 mIU/ml; fT3: 2.1-4.2 pg/ml; fT4: 7.1-18.5 pg/ml | WMS, CVSS, RPM, the Kohs’ Cubes, the Category Verbal Fluency | Memory Quotient and wmc- MQ were correlated with fT4 |
| Miulescu et al., 2018 [31] | Hungary | 12 patients with HT and type 2 diabetes mellitus | 91,7% females; mean age: 64.92±5.84; NI about mean time of education | mean TSH (mIU/L) 11.76±4.43; mean FT4 (ng/dL) 0.53±0.08; NI about mean fT3 and BMI | TSH: 0.4-4.5 mIU/L; fT4: 0.7-1.8 ng/dL). | MMSE | Negative correlation between TSH levels and MMSE |
| Osterweil et al., 1992 [33] | USA | 54 non-demented HT patients | 46% females; mean age 68.6 (16.4); mean education time: 11.2 (3.6) | mean TSH 66.3 (55.4)pu/mL; mean T3: 68.5 (41.2)ng/dL; mean T4 2.3 (2.2.); NI about mean BMI | TSH: 0.3-5.7 pu/mL; T4 4.2-11.0 pg/dL; T3 70-160 ng/dL | MMSE; Copying the Cube; IPALT; Animal Naming; DST (WAIS); SDMT, TMT; The Language Disorder tests were adapted from the BDAE | T4 was correlated with Inglis medium Association Test items and Animal Naming. Regression analysis for serum T3 did show trends for Inglis low Association, TMT- A, SDMT, and Digits Forward |
| Quinque et al., 2014 [47] | Germany | 18 patients with LT4 treated HT | 88,9% females; mean age 32 (9.6); NI about education | mean TSH (mU/l) 2.0 (1.1); mean fT3 (pmol/l) 4.3 (0.6); mean fT4 (pmol/l) 18 (1.6). NI about mean BMI | NI | WST, CVLT, WMS, TAP, TMT, PASAT | Significant correlation between fT3 and psychomotor speed (not survive correction for multiple comparisons) |
| Resta et al., 2012 [48] | Italy | 1 patient affected by HT, 42 with SCH | NI | NI | TSH: 0.3-3.6 mUI/L; FT4: 8.0-17.0 pg/mL; FT3: 2.2-4.2 pg/mL; TG-Abs: < 100 UI/mL; TPO Abs: <16 UI/ml | MMSE, PMT, and the Matrix Test | TSH negatively correlated with MMSE score (maintained at multiple linear regression) |
| Samuels et al., 2016 [49] | USA | 132 subjects receiving L-T4 therapy for HT, with TSH levels within the laboratory reference range (low-normal vs high-normal TSH) | 90,9% females; mean age in low-normal TSH: 48.9 ± 1.2 and 49.6 ± 1.9 in high normal TSH; mean years of education was 16.2 ± 0.3 in low-noemal TSH and 15.6 ± 0.5 in high-normal TSH | mean: TSH (mU/L) 1.35 ± 0.07 (low-normal); 3.60 ± 0.11 (high-normal); mean fT4 (ng/dL) 1.67± 0.04 (low-normal) 1.59 ± 0.05 (high-normal); mean fT3 (pg/dL) 215 ± 5 (low-normal) 208 ± 6 (high-normal) | TSH: 0.34–5.60 mU/L; fT4: 0.8–2.7 ng/dL; fT3: 210-440 pg/dL | Paragraph Recall; The Letter Cancellation Test.; TMT; IGT; N-back test; SOP; Pursuit Rotor; Motor sequence learning test | the IGT Net-1 (baseline) score was positively correlated with TSH levels; the 3-Back number incorrect nontarget was negatively correlated with fT4 levels; time to complete the TMT was positively correlated with fT3 levels |
| Yin et al., 2021 [50] | China | 18 patients with SCH elevated serum TSH levels | NI about % of females; mean age: 31 ±6; mean education: 11 ±2 | mean TSH (mIU/L): 9.32 ±2.43; mean FT3 (pmol/L): 4.46 ±0.63; mean FT4 (pmol/L): 9.72 ±1.25; NI about BMI | FT3 = 3.6–5.7 pmol/L; FT4 = 9.1–15.4 pmol/L; TSH = 0.51–4.85 mIU/L. | MoCA; ST | In the SCH group, the ST performance was negatively correlated with the TSH level; TSH levels were negatively correlated with the MoCA scores |
| Yuan et al., 2020 [51] | China | 31 patients with HT and 32 with SCH never treated with medicine (> 26 ponits in MMSE) | % females: 41,9 (HT), 37.5 (SCH); mean age: 35.52 ± 9.68 (HT), 32.06 ± 10.11 (SCH); mean time of education: 11.10 ± 3.24 (HT), 11.47 ± 3.25 (SCH) | mean TSH (μIU/mL): 101.97 ± 49.81 (HT), 12.72 ± 17.27 (SH); mean T3 (nmol/L): 0.60 ± 0.28 (HT), 1.48 ± 0.24 (SCH); mean T4 (nmol/L): 29.53 ± 14.70 (HT), 77.99 ± 11.48 (SCH); NI about mean BMI | TSH: 0.550–4.780 μIU/mL; T3: 0.92–2.79 nmol/L; T4: 58.10–140.60 nmol/L | MMSE, DST, ANT | In HT a negative association between T4 level and efficiencies of executive control network; significant association between T4 levels and efficiencies of the executive control network |

ABCT - AB clock test; AIT - autoimmune thyroiditis; ANT - attention network test; BDAE - Boston Diagnostic Aphasia Exam; CVLT - California Verbal Learning Test; CVSS - Corsi Visuo-Spatial Span; DST - digit span test; EUT – euthyroid; FT3 – free triiodothyronine; FT4 – free thyroxine; HT – hypothyroidism; IGT - The Iowa Gambling Task; IPALT - The Inglis Paired Associate Learning Test; LT4 – levothyroxine; MMSE - Mini Mental State Examination; MoCA - Montreal Cognitive Assessment; NI – no information; PASAT - Paced Auditory Serial Addition Test; PGIBBD - Postgraduate Institute Battery of Brain Dysfunction; PMT - the Prose Memory Test; SCH - subclinical hypothyroidism; ST - Stroop Task; RBMT - the Rivermead Behavioral Memory Test; ROCFT - the Rey-Osterrieth Complex Figure Test; RPM - the Raven’s Progressive Matrices; SDMT - Symbol Digit Modalities Test; SOP - Subject Ordered Pointing; T3 – triiodothyronine; T4 – thyroxine; TAP - Testbatterie zur Aufmerksamkeitspru¨fung; TMT - Trail Making Test; TSH - thyroid-stimulating hormone; WAIS - Wechsler Adult Intelligence Scale; WST – Wortschatztest; WMS - Wechsler Memory Scale
