# Supplementary material for: Prevalence, hormonal correlates, severity, and neural basis of neurocognitive impairment in patients with hypothyroidism: Systematic review and meta‐analyses
Source: Alzheimers Dement. 2025 Nov 26;21(11):e70924. doi: 10.1002/alz.70924 (PMC12657124; doi:10.1002/alz.70924)
Supplement: Supplementary file 6 — Supporting Information [file ALZ-21-e70924-s003.docx]

Supplementary Table 5. Jackknife Sensitivity Analysis of Studies Concerning Correlation Between MMSE and TSH Levels

| Studies omitted | Correlation | 95% CI | *I^2^* | *Tau*^2^ | *Q* |
| --- | --- | --- | --- | --- | --- |
| Djurovic, et al. (2018) | -0.53 | -0.75- -0.21 | 73% | 0.14 | 14.76** |
| Kamyshna, et al. (2022) | -0.47 | -0.75- -0.06 | 82% | 0.21 | 21.78** |
| Krausz et al. (2004) | -0.47 | -0.72- -0.10 | 85% | 0.19 | 26.72*** |
| Kumar et al. (2018) | -0.43 | -0.70- -0.04 | 83% | 0.18 | 23.24*** |
| Miulescu, et al. (2018) | -0.34 | -0.54- -0.10 | 75% | 0.05 | 16.28** |
| Resta et al. (2012) | -0.53 | -0.75- -0.19 | 84% | 0.16 | 24.37*** |

*-p<0.05; **-p<0.01; ***-p<0.001
