# Supplementary material for: Prevalence, hormonal correlates, severity, and neural basis of neurocognitive impairment in patients with hypothyroidism: Systematic review and meta‐analyses
Source: Alzheimers Dement. 2025 Nov 26;21(11):e70924. doi: 10.1002/alz.70924 (PMC12657124; doi:10.1002/alz.70924)
Supplement: Supplementary file 7 — Supporting Information [file ALZ-21-e70924-s005.docx]

Supplementary Table 6. Severity of neurocognitive impairment in patients with HT

| Authors  [country] | Sample size | Controls | Basic sociodemographic data | Clinical data | Hormone ranges | Cognitive tests | Depressive symptoms | Main findings |
| --- | --- | --- | --- | --- | --- | --- | --- | --- |
| Almeida, et al.  (2007) [55]  [Brazil] | 65 patients with SCH | 31 EUT matched for sex, age and educational level | 93,9% females; mean age: 48.1 [10.1]; NI about mean time of education | mean TSH: 8.1 ± 3.2 μU/mL; mean fT4: 1.1 ± 0.2 ng/dL; NI about mean fT3 and BMI | TSH: 0.4–4.0 μU/mL; fT4 level: 0.8–1.8 ng/dL | Vocabulary subtest (WAIS-R); BSRP; ROCFT; TCRMWF | NI | No statistically significant differences |
| Bajaj, et al.  (2014) [25]  [India] | 103 patients diagnosed with SCH | 103 age, sex and education‑matched healthy controls | 60.19% females; mean age: 75.74 ± 9.37; NI about mean time of education | NI | TSH concentration. >5.5 mIU/liter with serum‑free T4 and T3 concentration being within the reference range. | MMSE and CDT | NI | NCI was more common in SCH group than in controls. |
| Baldini, et al.  (1997) [52]  [Italy] | 19 goiter patients with SCH | 17 EUT goiter patients | 100% female; mean age: 55.2 (8.8); NI about mean education time | Pretreatment: TSH 12.0 ± 7.2; nIU/ml; FT3 3.6 ± 0.9 pg/ml; FT4 6.8 ± 0.7 | TSH: 0.5 and 4.6 yIU/ml; fT3: 2.6 and 5.6 pg/ml; fT4: 6.3 and 15.3 pg/ml | WMS, Scribble Test | 21 item HAM-D; no statistically significant differences between groups. | Logical memory scores (WMS) was lower in SCH patients. |
| Bocheva, et al. (2022) [56]  [Bulgaria] | 26 newly diagnosed HT patients | 26 euthyroid controls | 100% female; mean age 37.2± 9.8, NI about mean education time | Mean TSH (mlU/l) 16.9 ± 19.6; mean fT42 (pmol/l): 12.6 ± 3.19; Mean FT3 (pmol/l) 4.8 ± 0.99; NI about mean BMI | TSH, 0.3–4 mU/l; fT3: 3.5–7 pmol/l; fT4: 9–23 pmol/l | Participants had to determine whether the virtual center of radial GPs was shifted to the left or right of the screen center, or to discriminate between radial and concentric GPs with varying coherence. Three different conditions were applied: static and two dynamic flicker conditions with a limited lifetime of the dot pairs. | NI | The HT group had higher thresholds in discriminating the radial from the concentric patterns in the dynamic flicker conditions. The proportion of correct responses was lower in the HT group for patterns with the centers shifted to the right and for concentric compared to the radial patterns |
| Cao et al. (2023) [57]  China | 31 drug-naïve HT | 28 healthy controls (MoCA > 26 points) | 71% female; mean age 41.32±9.14, mean education time: 13.55± 3.70 | Mean TSH (mlU/l) 61.48± 31.85; mean fT4 (pmol/l): 6.51± 2.22; Mean fT3 (pmol/l) 3.95± 3.21; NI about mean BMI | TSH: 0.35 – 4.94 mIU/L; fT3: 2.43–6.01pmol/L, 9.01–19.05; fT4: pmol/L | MoCA | HAM-D  HT had significantly  higher scores | HT had lower scores than controls in MoCA+ |
| Ceresini et al. (2009) [58] [Italy] | 5 HT and 25 SCH aged >65 years | 800 EUT participants | HT: 60% females; mean age, 76.2 (8.0); mean time of education: 6.0 (3.5)  SCH: 72% females; mean age, 77.0 (7.4); mean time of education 5.6 (4.3) | HT: mean TSH 52 (34.63) lmIU/L; fT4 0.51 (0.21) ng/dL, fT3 2.91 (0.73) pg/mL.  SCH: TSH 7.99 (4.74), fT4 1.28 (0.31), fT3 4.28 (0.5) | TSH: 0.46–4.68 lmIU/L; fT4 0.77–2.19 ng/dL; fT3 2.77–5.27 pg/mL | MMSE | NI | No statistically significant differences |
| Constant et al. (2005) [59]  [Belgium] | 23 participants in remission after they had undergone total thyroidectomy; third measurement while HT | 26 control participants matched for age and sociocultural level | 65,2% females; mean age, 50.13; NI about mean time of education | TSH (mIU/l): 129.11 ± 60.56; fT4 (pmol/l): 4.12 ± 2.83. NI about mean fT3 and BMI | TSH: 0.2–3.5 mIU/l; fT4: 10.30–25.74 pmol/l | phasic alertness task from the TAP; Stroop test; | BDI; HT participants had higher BDI scores than the controls; no significant correlation between BDI and the performances on the tasks. | In Phasic alertness task, HT participants had slower RTs than did the controls. Stroop task revealed that, slower reaction times for neutral and incongruent items; a higher interference index |
| Correia et al. (2009) [41]  [Ireland] | 21 HT and 17 SCH patients | 19 healthy control matched by age and education | SCH: 94.1% females; mean age: 50.0 (9.2); mean education time: 12.8 (2.2); HT: 95,2% females; mean age: 44.0 (10.9); mean education time: 12.5 (3.1) | SCH: median TSH (mU/liter) 6.1; median FT4 (pmol/liter) 12.8; HT: median TSH (mU/liter) 38.9; median FT4 (pmol/liter): 7.6; NI about mean fT3 and BMI | NI | ROCFT; RBMT: Story Recall Subset; Face-Name Learning and Recall; Focused attention task; n-Back Task; the Stroop Task | HADS; correlation was observed between depression levels and verbal recall (Rivermead short stories, immediate level) | Significant deficit in HT at the copy level in ROCFT, and total face-name pairs successfully encoded. SCH and HT scored lower in memory accuracy score, recalled fewer units in Immediate Recall and Delayed Recall in the RBMT: Short-Stories Subset. No significant group differences at the n-Back Task, Focused attention task, and the Stroop Task. |
| Djurovic, et al. (2018) [42]  [Serbia] | 130 patients with HT on long-term LT4 treatment | 111 euthyroid control subjects | Age group 20–49 (N=59): NI about % of females; mean age: 35.82 ± 7.80; mean education time: 13.49 ± 2.45; Age group 50 + (N=71): NI about % of females; mean age: 61.23 ± 6.55; mean education time: 11.61 ± 3.92 | Age group 20–49: mean TSH (mIU/L) 3.64 ± 2.74; mean fT3(pmol/L) 2.45 ± 0.46; mean fT4 (pmol/L) 12.66 ± 2.87; mean BMI: 24.91 ± 3.67. Age group 50 +: mean TSH (mIU/L) 3.93 ± 2.84; mean fT3(pmol/L) 2.33 ± 0.49; mean fT4 (pmol/L) 12.11 ± 3.15; mean BMI: 27.20 ± 3.67 | TSH: 0.27–4.2 mIU/L; FT3: 2–4.25 ng/L; FT4: 7–18 ng/L | MMSE, Visual- and Digit Span, visual and verbal attention span from WAIS and Visual span from WMS-R, TMT, Phonemic fluency test | HADS; Patients reported significantly more depressive symptoms; depressive symptoms were correlated positively with TSH and negatively with the levels of TPOAb | MMSE scores and categorical fluency were higher in controls than in HT. TMT scores were higher in HT when compared to controls except for TMT-B in younger group of patients. Phonemic fluency test score was higher in controls only in younger group. |
| Ettleson et al. (2024) [60]  [USA] | 227 treated HT women | 1806 women without thyroid disease | 100% females; mean age 49.8 ± 2.6; NI about mean education time | TSH median (IQR) (mIU/L): 2.9 (1.2, 6.0); NI about fT3 and fT4 | TSH level (<0.4 or >4.5 mIU/L) | SDMT, DST backward, East Boston memory test | NI | Higher SMDT and DST backwards scores in HT pratients |
| Formiga, et al. (2014) [61]  [Spain] | 20 participants with SCH | 282 euthyroid subjects | 55% females, NI about mean age and education time | NI | 0.25–5 mU/l for TSH and 10–26 pmol/l for FT4; overt HT (TSH O5 mU/l, FT4 !10 pmol/l), SCH (TSH O5 mU/l, FT4 10–26 pmol/l) | MMSE | NI | No differences between the control group and SCH |
| Goyal et al. (2020) [43]  [India] | 30 drug‑naïve newly diagnosed HT patients before initiating treatment | 30 euthyroid age‑ and sex‑matched volunteers | NI about females %; mean age = 31.6 ± 8.4; NI about mean education time | TSH levels (μIu/mL): 26.43 ± 10.24; fT3 (pg/ml): 0.30 ± 0.09; fT4 (ng/dl): 0.27 ± 0.10. | NI | Stroop task | NI | No differences in RTs and correct responses |
| He, et al. (2011) [62]  [China] | 13 female patients before having been treated with LT4 | 12 euthyroid controls matched by age and education | 100% female, mean age 29.4 (6.3); Education 12.0(3.2) | Pre-treatment: mean TSH (mIU/l) 102.6 ± 57.8; mean fT3 (pmol/l) 2.3 ± 0.9; fT4 (pmol/l) 7.2 ± 1.9 | 3.5–6.5 pmol/l for fT3, 11.5–22.7 pmol/l for fT4, and 0.35–5.5 mIU/l for TSH. | WMS–CR; FMRI four-digit BR and FR task | NI | Patients showed significantly lower MQ than controls |
| Hu et al. (2016) [63]  China | 14 HT and 9 SCH patients recruited from the Memory Clinic≥ 50 years old | 260 controls | HT: 50% females, mean age 65.9 (10.9); mean education time: 11.6 (4.8)  SCH: 66.7% females, mean age 62.9 (11.7); mean education time: 7.4 (3.6) | Mean BMI: 23.0 (3.2); NI about mean TSH, fT3 and fT4 | HT: TSH >4.78 mIU/l; fT4 <11.50 and fT3 <3.50 pmol/l | MMSE, MES | NI | No differences in MMSE and MES between HT, SCH, controls and hyperthyroid participants |
| Jaiswal, et al. (2016) [24]  [India] | 36 adult SCH patients | 36 age‑ and sex‑matched euthyroid controls | 86.1% females; mean age: 35.5±5.9; NI about mean education time | Mean TSH (μIU/ml) 7.2 ± 2.5; mean FT3 (pg/ml) 2.7 ± 0.7; mean FT4 (ng/dl) 1.1 ± 1.0; mean BMI: 26.3 ± 3.5 | TSH: 0.34–4.24 μIU/ml; FT3: 2.0–4.2 pg/ml; FT4: 0.6–1.7 ng/dl | ABCT and DST | NI | SCH scored lower in standard DST than controls; no significant differences were observed in the ABCT |
| Jhandi et al. (2024) [64]  [India] | 99 SCH (33 newly diagnosed, 32 symptomatic on LT4, 34 EUT on LT4) with MMSE > cut - off point | 35 healthy controls | Newly diagnosed: 87.9% females; mean age: 37.24±8.68; NI about mean education time. Symptomatic on LT4: 96.9% females; mean age: 38.65±10.81; EUT on LT4: 94.1% females; mean age: 39.70±9.62 | Newly diagnosed: Mean TSH (mIU/L), 4.04±1.40; NI about mean fT3 and fT4. Symptomatic on LT4: TSH: 13.02±21.05; EUT on LT4: TSH: 2.54±0.91 | TSH  ≥2.5 mIU/L, TSH levels ≥4.0mIU/L - elevated TSH levels | Computerized  neuropsychological test for paired associative learning | HAM-D | Statistically significant main effect for Paired associate learning |
| Kalra, et al. (2020) [21]  [India] | 39 patients diagnosed with SCH | 23 euthyroid age, gender, and education matched controls | 92,3% females; mean age: 30.3 ± 7.7; NI about mean education time | TSH (mIU/L) 6.36 ± 1.31; NI about meean fT3, fT4, BMI | TSH below 4.5 mIU/L | MMSE; DSST, digit vigilance test, animal naming test, N-back test, tower of London, Stroop test, WCST, AVLT, ROCFT | NI | In SCH group more observations were below cut-off scores in fluency test and visual memory delayed recall. |
| Kamyshna, et al. (2022) [23]  [Ukraine] | 16 patients with postoperative HT; 65 patients with HT (autoimmune thyroiditis), and 72 patients with both autoimmune thyroiditis and elevated anti-Tg and anti-TPO. | 25 healthy controls without adjusting for age or gender | Postoperative HT: 100% females; mean age: 47.30±12.27; HT (autoimmune thyroiditis): 100% females; mean age: 46.72±15.49; autoimmune thyroiditis and elevated anti-Tg and anti-TPO: 100% females; mean age: 45.02±13.65; NI about mmean education times | Postoperative HT: mean TSH (mIU/mL): 8.61 ± 0.84; mean fT4 (pmol/L): 3.44 ± 0.31; HT (AIT): mean TSH (mIU/mL): 7.09 ± 0.50; mean fT4 (pmol/L): 4.13 ± 0.52; AIT and elevated anti-Tg and anti-TPO: mean TSH (mIU/mL): 2.38 ± 0.62; mean fT4 (pmol/L): 8.51 ± 0.82. NI about mean fT3 and BMI | TSH: 0.3–4.0 mIU/mL; fT4: 6.0–13.0 pmol/L for males and 7.0–13.5 pmol/L for females; anti-TPO:0–30 IU/mL; anti-TG: 0–65 IU/mL | MMSE | HAM-D; negative relationship between depression and anti-TPO and anti-TG | NCI was more prevalent in HT group than in controls |
| Kaur, et al. (2021) [27]  [India] | 100 patients with SCH | 100 age and sex matched controls | 69% females, mean age: 68.23 ± 6.95; NI about mean education time | mean TSH (mIU/L): 6.9 ± 2.72; mean fT3 (pM/L): 4.69 ± 1.24; mean fT4 (pM/L): 16.52 ± 2.99; mean BMI: 25.47 ± 2.33 | TSH: 0.27 - 4.0 μIU/mL; fT3: 1.3 - 3.1 nmol/L; fT4: 66 - 181 nmol/L | MMSE, and CDT. | NI | No statistically significant differences between the groups |
| Khorasani et al. (2019) [65]  [Iran] | 15 SCH (5˂TSH≤10 μIU/mL) and 15 SCH (10˂TSH≤15) | 15 euthyroid controls | SCH (5˂TSH≤10 μIU/mL) group: 60% female; mean age: 32.20±6.15. SCH (10˂TSH≤15) group: 66.7% females; mean age: 32.66±4.46. NI about mean education times | SCH (5˂TSH≤10 μIU/mL) group: mean TSH (μIU/mL): 6.87 ± 0.94 mean T3 (nmol/L): 1.18 ± 0.18; mean T4 (μg/dL): 7.52 ± 2.09. SCH (10˂TSH≤15) group: mean TSH (μIU/mL): 11.71 ± 1.30; mean T3 (nmol/L): 1.30 ± 0.35; mean T4 (μg/dL): 6.79 ± 1.16. NI about mean BMI | 0.27≤TSH≤5 | Bergen dichotic listening test | NI | Significant difference between the SCH (5˂TSH≤10 group) but not 10˂TSH≤15 group and controls in the scores of the right and left ear |
| Kramer, et al. (2009) [28]  [USA] | 149 HT participants with a positive history of physician-diagnosed primary HT who were currently using LT4 monotherapy | 885 euthyroid controls | 81.6% females; mean age: 76.1(9.6); NI about mean education time | mean TSH (mIU/l): 1.54 ± 1.59; mean BMI: 27.4 ± 5.5; NI about mean fT3 and fT4 | TSH: 0.49–4.67 mIU/l) | 3MSE; TMT B, and verbal fluency | BDI; no statistically significant differences between groups | No statistically significant differences between groups before and after adjusting for age and other covariates |
| Kumar, et al. (2018) [45]  [India] | 28 drug naive SCH patients | 28 healthy controls matched by age and education | NI about participant's sex; mean age 31.53 ± 8.10; mean education 12.96 ± 3.08 | mean TSH 9.61 ± 1.39 μIU mL-1, NI about mean fT3 and fT4; mean BMI 24.71 ± 4.83 | NI | MMSE and PGIBBD | NI | SCH showed a significant difference in attention and concentration, delayed recall, visual retention, recognition, bender visual motor gestalt (BGT) and MMSE |
| Kumar et al. (2025) [66]  [India] | 25 drug naïve HT patients | 30 age-matched controls | 84% females; mean age: 31.4 (10.54); mean education time: 12.37 (3.54) | mean TSH 158.37 ± 137.42 μIU mL-1, NI about mean fT3 and fT4; mean BMI 24.75 ± 4.85 | NI | MMSE; PGIBBD; BSR-R; NBT; BGT | NI | HT scored lower on the MMSE, Long term episodic memory, Mental balance, Delayed Recall, Visual Retention, Recognition of objects, digit span, and BGT |
| Lesiv, (2020) [29]  [Ukraine] | 18 patients with HT who received LT4 at a dose of 100-150 mg; | 18 practically healthy persons | NI about % of females and education time; mean age: 49.24 ± 0.83. | mean TSH, mIU/L: 3.16 ± 0.79. | TSH: <4.4 mIU/L | MMSE, ACE-R | NI | HT scored lower on MMSE and ACE-R than healthy controls. |
| Lesiv, et al. (2021) [67]  [Ukraine] | 18 patients with HT who received L-thyroxine at a dose of 100-150 mg | 18 people representative of age and sex ratio without a history of hypertension, and HT | NI | NI | NI | Schulte Tables, Learning 10 words (Luria), DST from Mattisse scale, Munsterberg Technique, CDT | NI | Patients with HT scored lower than controls on Selectivity of attention and Schulte Tables. |
| Leyhe, et al. (2008) [68]  [Germany] | 26 euthyroid patients with Hashimoto’s thyroiditis | 25 euthyroid patients undergoing hormonal treatment for goitre or after thyroid surgery | 88,5% females; mean age: 46.0 ± 1.9; NI about mean education time | mean TSH (mU/l) 1.2 ± 0.1; mean fT4 (pmol/l) 19.0 ± 0.6; mean fT3 (pmol/l) 4.8 ± 0.1; mean BMI 26.8 ± 1.1 | NI | TMT, DSST of the WAIS, d2, ROCFT, AVLT, DST of WAIS, vocabulary test (MWT-B) | NI | No statistically significant differences between the groups |
| Leyhe, et al. (2013) [46]  [Germany] | 18 Hashimoto thyroiditis patients on stable LT4 treatment, and TSH concentration of 0.4–2.5 mU/l. Diagnosis of HT was based on high levels of TPOAbs and/or Tg-Abs, associated with a hypoechogenic pattern as assessed with thyroid ultrasound. | 12 controls; on L-thyroxine treatment for goitre or after thyroid surgery; matched by gender, age, education, and task performance in the d2 test (as assessed 6–10 weeks before magnetic resonance imaging) | 84,6% females; mean age 43 ± 12; mean education 13 ± 4 | mean TSH (mU/l) 1.5 ± 1.0; mean Free T4 (pmol/l) 15.5 ± 2.9; mean Free T3 (pmol/l) 4.8 ± 0.6; mean BMI 25.8 ± 5.1 | TSH, 0.4–2.5 mU/l; free T4, 12–23 pmol/l; free T3, 3.5–6.5 pmol/l; TPOAbs,<100 IU/l; Tg-Abs,<100 IU/l. | d2 test | NI | No significant differences were detected between the groups |
| Liu, et al. (2020) [69]  [China] | 18 newly diagnosed HT patients with elevated TSH and lowered fT4 and fT3 | 18 age-, weight- and sex-matched healthy controls | 72,2% female; mean age: 43.06 ± 7.04; mean education: 13.22 ± 1.52 | mean TSH (mIU/l): 95.3 ± 34.51; mean fT3 (pmol/l): 1.98 ± 0.63; mean fT4 (pmol/l): 5.96 ± 1.59. | TSH: 0.55–4.78 mIU/L; fT3: 3.5–6.5 pmol/L; fT4: 11.5–22.7 pmol/L | MoCA; WMS | BDI-II | Patients with HT showed worse MoCA and MQ scores than healthy controls. |
| Menicucci, et al. (2013) [39]  [Italy] | 17 patients with SCH | 17 euthyroid control individuals | 100% females; mean age, 28 ± 4; NI about mean education time | median TSH: 5.3; median fT3: 2.7; median fT4: 10.9; NI about mean BMI | TSH: 0.3-3.8 mIU/ml; fT3: 2.1-4.2 pg/ml; fT4: 7.1-18.5 pg/ml | WMS, the Corsi Visuo-Spatial Span, the Raven’s Progressive Matrices, the Kohs’ Cubes, CVFT | HAM-D | SCH showed significantly higher than controls in Mental Control |
| Miller, et al. (2006) [70]  [USA] | 14 patients with HT confirmed by elevated basal TSH levels | 10 controls matched by age, sex, education, ethnicity, and handedness | 78,6% females; mean age: 42.8 ± 12.4; mean years of education: 15.8 ± 2.7 | mean TSH (mcIU/ml): 31.5 ± 56.2; NI about mean fT3, fT4 and BMI | TSH (mcIU/ml): 4.9–1.4 | Logical Memory test (WMS—III), CVLT, ROCFT, TMT, COWAT, Animal Naming | HAM-D | Significant differences between groups in CVLT: Delay Free Recall, Long Delay Free Recall, and Long Delay Cued Recall |
| Miller et al. (2007) [71]  [USA] | 14 patients with HT confirmed by elevated basal TSH levels | 10 controls matched by age, sex, education, ethnicity, and handedness | 78,6% females; mean age: 42.8 ± 12.4; mean years of education: 15.8 ± 2.7 | mean TSH (mcIU/ml): 31.5 ± 56.2; NI about mean fT3, fT4 and BMI | NI | Logical Memory test (WMS—III), CVLT, ROCFT, TMT, COWAT, Animal Naming | HAM-D | Significant differences in CVLT: Delay Free Recall, Long Delay Free Recall, and Long Delay Cued Recall |
| Mishra, et al. (2016) [72]  [India] | 29 newly diagnosed HT patients | 29 age & education matched normal euthyroid controls | 100% females; mean age: 29.9 ± 6.74; NI about mean education time | NI about units; mean TSH: 33.23 ± 35.01; mean T3: 0.51 ± 0.48 | NI | Digit vigilance test; CTT; Triads test | NI | The scores were significantly higher in HT group |
| Mishra, et al. (2018) [73]  [India] | 29 newly diagnosed HT patients | 29 age- and education-matched normal euthyroid controls | 100% females; mean age: 24.12± 3.07; NI about mean education time | NI about units; mean TSH: 33.23 ± 35.01; mean T3: 0.51 ± 0.48 | NI | Digit vigilance test; CTT; Triads test | NI | The scores were significantly higher in HT group |
| Monzani et al. (1993) [74]  [Italy] | 14 patients with a diagnosis of SCH | 50 age- and sex- matched healthy euthyroid subjects | 92,9% females; mean age: 38.7_+ 9.0 years; NI about mean time of education | TT4 (I.tg/dl): 7.1 ± 0.3; TTs (ng/dl): 125.4 ± 5.2; fT4 (pg/ml): 7.0 ± 0.4; fT3 (pg/ml): 3.0 ±.0.1; TSH (mlU/1): 8.8 ± 1.5; NI about mean BMI | TSH: 0.4-5 mIU/1; TT4: 4.5-12.5 pg/dl; TT3: 80-200 ng/dl; fT4: 5.3-15.6 pg/ml; fT3: 2.8-5.6pg/ml | WMS | CCEI; before treatment patients had higher depressive symptoms severity than controls | Before treatment significant difference was found for all scales except for mental control |
| Oerbeck, et al. (2005) [53]  [Norway] | 12 young adults with an early treated severe form of congenital HT | 12 siblings matched by age at assessment, gender or education. | 50% females; mean age 20.3 ± 0.9; mean time of education: 11.8 ± 1.0 | mean TSH: 35.3 ± 38.8 mU/L; mean fT3: 13.8 ± 5.6 pmol/L; NI about mean fT4 and BMI | NI | NI | NI | The memory deficits were on all verbal measures; significant deficit in distractibility |
| Osterweil, et al. (1992) [33]  [USA] | 54 non-demented HT patients | 30 euthyroid controls matched by age, sex and education | 46% females; mean age 68.6 ± 16.4; mean education time: 11.2 ± 3.6 | mean TSH 66.3 ± 55.4 pu/mL; mean T3: 68.5 ± 41.2 ng/dL; mean T4 2.3 ± 2.2; NI about mean BMI | TSH: 0.3-5.7 pu/mL; T4 4.2-11.0 pg/dL; T3 70-160 ng/dL | MMSE; Copying the Cube; The Inglis Paired Associate Learning Test; Animal Naming; DST (WAIS); SDMT, TMT; The Language Disorder tests from BDAE | NI | HT patients scored significantly lower than controls on MMSE, Copying the Cube, Inglis low and medium association pairs, Animal Naming, and TMT A, DST Backward and SDMT |
| Pandey, et al. (2017) [75]  [India] | 11 hypothyroid patients | 8 healthy controls matched on age and education | 90% females; mean age: 31.91 ± 8.39; mean time of education 14.72 ± 1.95 | NI | NI | AVLT | NI | No statistically significant differences |
| Park, et al. (2010) [76]  [Republic of Korea] | 164 SCH aged at least 65 years, who did not have any thyroid diseases | 754 euthyroid controls | 55,5% females; mean age: 76.3 ± 8.9; mean education time: 5.1 ± 4.8 | mean TSH (mIU/l): 6.79 ± 3.77; mean fT4 (ng/dl): 1.17 ± 0.29; mean BMI: 24.0 ± 3.4; NI about mean fT3 | TSH: 0.4–4.1 mIU/l; fT4: 0.7–1.8 ng/dl | DST, FAB, CERAD; MMSE; CVFT; MBNT, CPT, WLMT, WLRT, WLRcT; CRT. | GDS; GDS scores did not differ by the presence of SCH | MMSE, FAB, WLMT and WLRT scores of the SCH group were slightly better than those of the euthyroid group. |
| Quinque, et al. (2014) [47]  [Germany] | 18 patients with levothyroxine treated hypothyroidism | 18 healthy controls matched for age, sex, intelligence and TSH | 88,9% females; mean age 32 ± 9.6; NI about education | mean TSH (mU/l) 2.0 ± 1.1; mean fT3 (pmol/l) 4.3 ± 0.6; mean fT4 (pmol/l) 18 ± 1.6; NI about mean BMI | NI | WST; CVLT; WMS; Testbatterie zur Aufmerksamkeitsprufung, TMT, PASAT | BDI | Significant group difference showed better learning performance in the patient than the control group, but would not survive multiple comparison correction. |
| Resta, et al. (2012) [48]  [Italy] | 42 subjects with SCH; subjects without a previous diagnosis of thyroid dysfunction and/or treated with drugs known to affect thyroid function | 283 euthyroid subjects | NI | NI | TSH: 0.3-3.6 mUI/L; FT4: 8.0-17.0 pg/mL; FT3: 2.2-4.2 pg/mL; TG-Abs: < 100 UI/mL; TPO Abs: <16 UI/ml | MMSE, PMT, Matrix Test | Geriatric Depression Scale (GDS) | MMSE, PMT of subjects with SCH were lower than euthyroid; SCH, had a probability about 2 times greater of developing NCI, regardless of sex, smoking, GDS, hypertension, diabetes mellitus, chronic heart failure, stroke and Parkinson's disease |
| Samuels et al. (2007) [77]  [USA] | 34 women treated with L-T4 | 20 women with no history of thyroid disease | 100% females; mean age: 35.3 ± 1.3; mean time of education: 15.8 ± .04 | mean TSH (mU/L): 2.550.19; mean fT4 (pmol/L): 17.00.4; mean fT3 (pmol/dL): 4.10.1; mean BMI: 28.80. | TSH: 0.28–5.00mU=L.; Free T4: 9.01–23.17pmol=L; Free T3: 3.23–6.76 pmol=L. | Paragraph Recall subtest of the WMS-R, The Medical College of Georgia (MCG) Complex Figure Test, N-Back Test, SOP, DST Backwards, Pursuit Rotor Motor Learning Test | SCL-90-R, POMS; depression measured with SCL-90-R subscale, was significantly worse in the HT group compared to the controls; no group differences in mood (POMS subscales) | Treated HT subjects performed worse in copying the complex figure (this effect was no longer significant after adjusting for the potential confounders); N-Back number correct in the HT group was significantly worse after adjustment for confounding factors. Pursuit Rotor trials 1, 2, and 4 were worse in HT |
| Schraml, et al. (2011) [78]  [USA] | 11 subjects who had undergone a thyroidectomy for treatment of thyroid carcinoma | 11 controls matched in terms of age, education, and handedness | 54,6% females; mean age: 33.0 ± 8.8; mean time of education: 16.5 ± 2.4 | HT: TSH (uIU/mL) 119.8 ± 77.7; fT4 (ng/dL) <0.4; fT3 (pg/dL) <160.0; NI about mean BMI | TSH: 0.49 to 4.67 uIU/mL; free T4: 0.71 to 1.85 ng/dL; free T3: 230 to 420 pg/dL | Grooved Pegboard Test (both hands), TMT, TWT, ROCFT, ACT, BSRT; WMS-III Logical Memory, WMS-III Visual Reproduction, Working Memory Index, PASAT, WCST | BDI-2; significant difference between the subjects and controls during HT state | When HT, there was a statistically significant difference in The Working Memory Index |
| Sheng et al. (2024) [79]  [USA] | 17 subjects with TSH > 4.5 mIU/L | 380 controls with TSH between 0.45 and 4.5 | NI | NI | HT: TSH > 4.5 mIU/L and FT4  < 0.6 ng/dL. | CERAD, animal fluency, DSST | NI | Statistically significant main effect for CERAD1 and CERAD delay recall (TSH: <0.45 vs 0.45–4.5 vs >4.5) |
| Singh, et al. (2014) [80]  [India] | 8 newly diagnosed HT patients with elevated TSH and low free T4 (FT4) levels | 8 healthy controls | 62.5% females; mean age 32.8 ± 9.22; mean education time 11.3 ± 5.55 | Mean fT4 6.68±2.20 pmol/l; mean TSH 51.28 ± 36.05 μIU/ml | NI | PGIMS | NI | HT group had a significantly worse scores in PGIMS |
| Su, et al. (2023) [36]  [China] | 44 newly diagnosed patients with HT with MMSE score equal or higher than 24 points | 54 matched by age, sex, and education level | 77.3% female; mean age 39.30 ± 9.80; mean education 13.57 ± 3.45 | median TSH: 44.81 mIU/L; mean fT3, pmol/L, 3.30 ± 1.18; mean fT4, pmol/L: 6.81 ± 2.22; NI about mean BMI | TSH: 0.35–4.94 mIU/L; fT4: 9.01–19.05 pmol/L | MMSE; MoCA | HAM-D | Patients with HT had a lower results in MMSE, and MoCA |
| Wijsman, et al.. (2013) [81]  [Netherlands, Scotland, Switzerland] | 161 participants with SCH aged 70–82 years with pre-existing vascular disease or more than one risk factor to develop this condition. Patients taking antithyroid medications, thyroid hormone supplementation and/or amiodarone were excluded | 4,928 euthyroid controls matched by age and education | 64.0% females; mean age: 75.63 ± 0.25; mean education time: 15.17 ± 0.17 | Mean BMI: 26.88 ± 0.33; NI about means TSH, fT3, fT4 | TSH: 0.45 mU/L to 4.50 mU/L; FT4: 12 pmol/L to 18 pmol/L | MMSE, SCWT, Letter-Digit Coding Test, The Picture-Word Learning Test | NI | No statistically significant differences between the groups |
| Yamamoto et al. (2012) [54]  [Japan] | 15 SCH free of diagnosed dementia receiving no treatment for thyroid-related disease and did not take thyroid or anti-thyroid drugs | 224 controls | 53.3% females; mean age: 80.1 ± 4.3; NI about mean time of education | mean TSH (mIU/ml): 6.35 ± 3.44; mean fT4 (ng/dL): 0.99 ± 0.13; mean BMI: 23.4 ± 4.3; NI about mean fT3 | TSH > 4.0 i/mL | MMSE and Revised Hasegawa Dementia Scale (HDSR) | NI | The MMSE and HDSR results did not differ between groups |
| Yin, et al. (2013) [82]  [China] | 16 patients with SCH treated with LT 4 | 16 healthy volunteers matched for sex, age and years of education | 100% females; mean age: 34 ± 88; mean education time 11 ± 2 | mean TSH (mIU/ml): 19.43 ± 9.25; mean fT3 (pmol/l): 4.01 ± 0.27; mean fT4 (pmol/l): 8.57 ± 0.91; NI about BMI | TSH: 0.34–5.6 mIU/ml; fT3: 3.8–6.0 pmol/l; fT4: 7.86–14.41 pmol/l | WMS; n-back task | NI | Significant differences in figural memory, visual regeneration, visual recognition, associative learning, touch test, understanding memory numeric span, and in the subtest of mental memory and memory quotient. The accuracy between 1-back and 2-back was lower in HT group; RTs was lower in HT group in the 2-back |
| Yin, et al. (2021) [50]  [China] | 18 patients with SCH elevated serum TSH levels | 18 healthy controls matched for age and education | NI about % of females; mean age: 31 ± 6; mean education: 11 ± 2 | mean TSH (mIU/L): 9.32 ± 2.43; mean fT3 (pmol/L): 4.46 ± 0.63; mean fT4 (pmol/L): 9.72 ± 1.25; NI about BMI | FT3 = 3.6–5.7 pmol/L; FT4 = 9.1–15.4 pmol/L; TSH = 0.51–4.85 mIU/L. | MoCA; the Stroop task | NI | Patients with SCH had statistically significant lower scores in MoCA |
| Yuan, et al. (2020) [51]  [China] | 31 patients with HT and 32 with SCH never treated with medicine (> 26 ponits in MMSE) | 37 healthy controls | % females: 41,9 (HT), 37.5 (SCH); mean age: 35.52 ± 9.68 (HT), 32.06 ± 10.11 (SCH); mean time of education: 11.10 ± 3.24 (HT), 11.47 ± 3.25 (SCH) | mean TSH (μIU/mL): 101.97 ± 49.81 (HT), 12.72 ± 17.27 (SH); mean T3 (nmol/L): 0.60 ± 0.28 (HT), 1.48 ± 0.24 (SCH); mean T4 (nmol/L): 29.53 ± 14.70 (HT), 77.99 ± 11.48 (SCH); NI about mean BMI | TSH: 0.550–4.780 μIU/mL; T3: 0.92–2.79 nmol/L; T4: 58.10–140.60 nmol/L | MMSE, DST,ANT | HAMD (version: 17-item); HAMD scores in patients with hypothyroidism were significantly higher than in the other three kinds of thyroid dysfunction | The alerting network efficiency of healthy controls was significantly higher than that of the HT. |
| Zhu, et al. (2006) [83]  [China] | 9 HT and 11 SCH patients first diagnosed not treated with medicine or surgery | 12 euthyroid control subjects matched for age and level of education | HT: 100% females; mean age: 31.44 ± 9.29; mean education 5.67 ± 3.24; SCH - baseline: 90,9% females; mean age: 30.55 ± 9.68; mean education 9.09 ± 4.61 | HT: mean TSH mIU/ml: 44.68±19.60; mean fT3 pmol/l: 1.98 ± 0.53; mean fT4 pmol/l: 5.91 ± 2.02; SCH: mean TSH mIU/ml: 14.67 ± 7.13; mean fT3 pmol/l: 3.05 ± 0.45; mean fT4 pmol/l: 13.01 ± 3.27; NI about mean BMI | TSH (mIU/l): 0.4–4.0; FT3 (pmol/l): 2.3–6.3; FT4 (pmol/l): 8.4–29.6 | WMS; n-back task | NI | In the 2-back task, the lower accuracy of HT patients; the SCH subject performed worse in the 2-back task than euthyroid and HT subjects. In the 1-back task, lower accuracy of the HT patients than the other subject groups. |

ABCT - AB clock test; ACE-R - the Addenbrooke’s cognitive examination scale; ACT - Auditory Consonant Trigrams; AIT - autoimmune thyroiditis ANT - attention network test; Anti-Tg - Antithyroglobulin antibodies; anti-TPO - Thyroid Peroxidase Antibodies; ART-90 - Act-React Testsystem; AVLT - auditory verbal learning test; BDAE - the Boston Diagnostic Aphasia Exam; BDI - Beck Depression Inventory; BGT - Bender Gestalt Test; BMI – Body Mass Index; BSR-R - Short Battery of Performance Tests of Intelligence; BSRP - Buschke’s Selective Reminding Procedure; BSRT - Buschke Selective Reminding Test; CCEI - Crown and Crisp Experiential Index; CDT - clock drawing test; CERAD - Consortium to Establish a Registry for Alzheimer’s Disease; COWAT - Controlled Oral Word Association Test; CPT - the constructional praxis test; CRT - the constructional recall test; CTT - Colour trails test; CVFT - the categorical verbal fluency test; CVLT - the California Verbal Learning Test; DR2 - Simple choice reaction test; DSST - Digit Symbol Substitution Test; DST - Digit span test; EUT – euthyroid; FAB - the frontal assessment battery; FT3 – free triiodothyronine; FT4 – free thyroxine; GDS - geriatric depression scale; GPs - Glass patterns; HADS - Hospital Anxiety and Depression Scale; HAM-D - Hamilton Rating Scale for Depression; HT – hypothyroidism; LL5 - Line Labyrinth Test; LT4 – L-thyroxine; MBNT - the 15-item modified Boston naming test; MLS - Motor Performance Series; MMSE - Mini Mental State Examination; MoCA - Montreal Cognitive Assessment; MQ – Memory Quotient; NBT - Nahar–Benson Test; NCI – neurocognitive impairment ; NI – no information; PASAT - Paced Auditory Serial Addition Test; PGIBBD - Postgraduate Institute Battery of Brain Dysfunction; PGIMS - Postgraduate Institute memory scale test battery; PMT - the Prose Memory Test; RBMT - the Rivermead Behavioral Memory Test; RCFT - Rey Complex Figure Test; ROCFT - the Rey-Osterrieth Complex Figure Test; RST3 - Multiple-choice reaction test; RTs - reaction times; SCH – subclinical hypothyroidism; SCWT - The Stroop-Colour-Word- Test; SDMT - Symbol Digit Modalities Test; SOP - Subject Ordered Pointing; T3 – triiodothyronine; T4 – thyroxine; TAP - Test Battery for Attentional Performance; TCRMWF - Two-Choice Recognition Memory for Words and Faces; TMT - Trail Making Test; TSH - thyroid-stimulating hormone; TT15 - Tachistoscope test; TWT - Thurstone Word Fluency; WAIS – III - Wechsler Adult Intelligence Scale-III; WAIS-R - revised version of the Wechsler Adult Intelligence Scale; WCST - Wisconsin card sorting test; WLMT - the word list memory test; WLRcT - the word list recognition test; WLRT - the word list recall test; WMS - Wechsler Memory Scale; WMS-R - Wechsler Memory Scale-Revised; WST – Wortschatztest
