# Supplementary material for: Prevalence, hormonal correlates, severity, and neural basis of neurocognitive impairment in patients with hypothyroidism: Systematic review and meta‐analyses
Source: Alzheimers Dement. 2025 Nov 26;21(11):e70924. doi: 10.1002/alz.70924 (PMC12657124; doi:10.1002/alz.70924)
Supplement: Supplementary file 8 — Supporting Information [file ALZ-21-e70924-s010.docx]

Supplementary Table 7. Severity of neurocognitive impairment: Jackknife analyses

| Tool | Studies omitted | SMDs | 95%-CI | *I^2^* | *Tau*^2^ | *Q* |
| --- | --- | --- | --- | --- | --- | --- |
| MMSE | All studies included | -1.1339 | [-2.0198; -0.2480] | 99.2% | 3.1853 | 1844.14*** |
|  | Ceresini et al., 2009: HT | -1.1951 | [-2.1331; -0.2572] | 99.2% | 3.3590 | 1836.10*** |
|  | Ceresini et al., 2009 SCH | -1.2130 | [-2.1479; -0.2781] | 99.2% | 3.3264 | 1787.47*** |
|  | Djurovic et al., 2018: age groups 20–49 yrs | -1.1659 | [-2.1132; -0.2186] | 99.2% | 3.4169 | 1823.20*** |
|  | Djurovic et al., 2018: age groups 50 + yrs | -1.1419 | [-2.0908; -0.1929] | 99.2% | 3.4303 | 1840.41*** |
|  | Formiga et al., 2014 | -1.1904 | [-2.1323; -0.2484] | 99.2% | 3.3788 | 1818.40*** |
|  | Kumar et al., 2018 | -1.1464 | [-2.0947; -0.1982] | 99.2% | 3.4270 | 1841.00*** |
|  | Kumar et al., 2025 | -1.1260 | [-2.0740; -0.1780] | 99.2% | 3.4260 | 1843.73*** |
|  | Lesiv et al., 2020 | -0.9966 | [-1.8944; -0.0988] | 99.2% | 3.0755 | 1831.56*** |
|  | Osterweil et al., 1992 | -0.8291 | [-1.5307; -0.1275] | 99.2% | 1.8513 | 1769.75*** |
|  | Park et al., 2010 | -1.2251 | [-2.1556; -0.2947] | 99.1% | 3.2919 | 1530.47*** |
|  | Resta et al., 2012 | -1.1886 | [-2.1315; -0.2458] | 99.2% | 3.3840 | 1794.70*** |
|  | Su et al., 2023 | -1.1841 | [-2.1278; -0.2404] | 99.2% | 3.3911 | 1816.32*** |
|  | Wijsman et al., 2013 | -0.8821 | [-1.6703; -0.0938] | 93.5% | 2.3383 | 216.93*** |
|  | Yamamoto et al., 2012 | -1.2347 | [-2.1594; -0.3099] | 99.2% | 3.2549 | 1795.92*** |
|  | Yuan et al., 2020: HT | -1.2146 | [-2.1485; -0.2807] | 99.2% | 3.3204 | 1803.71*** |
|  | Yuan et al., 2020: SCH | -1.2111 | [-2.1463; -0.2758] | 99.2% | 3.3304 | 1805.86*** |
| TMT A | All studies included | 1.1085 | [-0.2889; 2.5059] | 92.0% | 2.9128 | 62.11*** |
|  | Djurovic et al., 2018: age groups 20–49 yrs | 1.2001 | [-0.5258; 2.9260] | 93.5% | 3.7173 | 61.70*** |
|  | Djurovic et al., 2018: age groups 50 + yrs | 1.1583 | [-0.5747; 2.8914] | 93.5% | 3.7533 | 61.74*** |
|  | Miller et al., 2006 | 1.2815 | [-0.3944; 2.9573] | 93.4% | 3.5250 | 60.39*** |
|  | Miller et al., 2007 | 1.3480 | [-0.2774; 2.9734] | 93.0% | 3.3079 | 57.31*** |
|  | Osterweil et al., 1992 | 0.5315 | [0.2012; 0.8618] | 39.5% | 0.0574 | 6.61 |
|  | Quinque et al. 2014 | 1.2859 | [-0.3931; 2.9649] | 93.3% | 3.5263 | 59.27*** |
| TMT B | All studies included | 0.3453 | [-0.0294; 0.7200] | 78.2% | 0.1778 | 27.51*** |
|  | Djurovic et al., 2018: age groups 20–49 yrs | 0.3267 | [-0.1336; 0.7869] | 80.8% | 0.2400 | 26.11*** |
|  | Djurovic et al., 2018: age groups 50 + yrs | 0.2298 | [-0.1259; 0.5855] | 67.9% | 0.1189 | 15.59** |
|  | Kramer et al., 2009 | 0.4400 | [0.0326; 0.8475] | 64.3% | 0.1658 | 14.02* |
|  | Miller et al., 2006 | 0.3440 | [-0.0842; 0.7722] | 81.8% | 0.2180 | 27.45*** |
|  | Miller et al., 2007 | 0.4445 | [0.0870; 0.8020] | 79.0% | 0.1353 | 23.85*** |
|  | Osterweil et al., 1992 | 0.2535 | [-0.1337; 0.6407] | 76.7% | 0.1589 | 21.48*** |
|  | Quinque et al. 2014 | 0.3689 | [-0.0651; 0.8029] | 81.8% | 0.2190 | 27.48*** |
| DST forward | All studies included | -1.3308 | [-3.0961; 0.4345] | 96.4% | 5.553 | 164.97*** |
|  | Menicucci et al., 2013 | -1.6055 | [-3.6126; 0.4016] | 96.9% | 6.165 | 163.35*** |
|  | Monzani et al., 1993 | -0.4834 | [-1.1578; 0.1910] | 92.8% | 0.639 | 69.28*** |
|  | Osterweil et al., 1992 | -1.2276 | [-3.3152; 0.8600] | 85.8% | 6.674 | 119.03*** |
|  | Park et al., 2010 | -1.6003 | [-3.6178; 0.4172] | 95.5% | 6.212 | 110.32*** |
|  | Quinque et al., 2014 | -1.5178 | [-3.5813; 0.5456] | 97% | 6.522 | 164.85*** |
|  | Yuan et al., 2020: SCH | -1.5074 | [-3.5787; 0.5639] | 97% | 6.564 | 164.32*** |
|  | Yuan et al., 2020: HT | -1.4244 | [-3.5235; 0.6747] | 96.8% | 6.746 | 157.06*** |
| DST Backwards | All studies included | -1.2438 | [-2.5795; 0.0918] | 96.6% | 4.0764 | 233.29*** |
|  | Ettleson et al., 2024 | -1.4279 | [-2.8964; 0.0406] | 96.8% | 4.3751 | 219.97*** |
|  | Menicucci et al., 2013 | -1.3930 | [-2.8767; 0.0906] | 97.0% | 4.4823 | 233.25*** |
|  | Monzani et al., 1993 | -0.8099 | [-1.9673; 0.3475] | 95.3% | 2.7079 | 150.03*** |
|  | Osterweil et al., 1992 | -0.7711 | [-1.8592; 0.3171] | 94.0% | 2.3770 | 116.07*** |
|  | Park et al., 2010 | -1.4355 | [-2.8989; 0.0279] | 96.8% | 4.3444 | 221.32*** |
|  | Quinque et al., 2014 | -1.4071 | [-2.8835; 0.0693] | 97.0% | 4.4372 | 233.26*** |
|  | Samuels et al., 2007 | -1.2881 | [-2.8054; 0.2292] | 96.9% | 4.6889 | 223.35*** |
|  | Yuan et al., 2020: SCH | -1.3346 | [-2.8435; 0.1743] | 96.9% | 4.6329 | 228.07*** |
|  | Yuan et al., 2020: HT | -1.3307 | [-2.8407; 0.1792] | 96.9% | 4.6395 | 227.59*** |
| Fluency | All studies included | -0.4478 | [-0.9353; 0.0396] | 95.9% | 0.362 | 146.41*** |
|  | Djurovic et al., 2018: age groups 20–49 yrs | -0.4517 | [-1.0295; 0.1261] | 96.5% | 0.441 | 144.56*** |
|  | Djurovic et al., 2018: age groups 50 + yrs | -0.3748 | [-0.9303; 0.1808] | 96.6% | 0.406 | 145.33*** |
|  | Kramer et al., 2009 | -0.2473 | [-0.6428; 0.1482] | 81.2% | 0.168 | 26.64*** |
|  | Menicucci et al., 2013 | -0.5684 | [-1.0666; -0.0701] | 96.4% | 0.325 | 137.97*** |
|  | Miller et al., 2006 | -0.4468 | [-1.0016; 0.1080] | 96.6% | 0.422 | 146.19*** |
|  | Miller et al., 2007 | -0.4433 | [-0.9982; 0.1115] | 96.6% | 0.422 | 146.24*** |
|  | Park et al., 2010 | -0.5867 | [-1.0871; -0.0864] | 90.1% | 0.309 | 50.66*** |
| WMS MQ | All studies included | -1.2857 | [-2.1099; -0.4615] | 87.6% | 1.078 | 48.54*** |
|  | Baldini et al., 1997 | -1.3249 | [-2.2995; -0.3504] | 89.7% | 1.318 | 48.38*** |
|  | He et al., 2011 | -1.3057 | [-2.2762; -0.3352] | 89.7% | 1.316 | 48.54*** |
|  | Liu et al., 2020 | -1.1621 | [-2.0905; -0.2336] | 88.6% | 1.189 | 43.79*** |
|  | Menicucci et al. 2013 | -1.5600 | [-2.3031; -0.8168] | 80.3% | 0.696 | 25.32*** |
|  | Monzanil et al., 1993 | -0.9612 | [-1.6031; -0.3193] | 77.2% | 0.487 | 21.91*** |
|  | Yin et al., 2013 | -1.2857 | [-2.2609; -0.3104] | 89.7% | 1.325 | 48.46*** |
|  | Zhu et al., 2006: SCH | -1.4023 | [-2.3381; -0.4664] | 89.2% | 1.212 | 46.17*** |
| WMS MC | All studies included | -1.0748 | [-1.6250; -0.5246] | 63.6% | 0.251 | 10.99* |
|  | Baldini et al., 1997 | -1.2331 | [-1.8311; -0.6351] | 58.7% | 0.222 | 7.27 |
|  | Menicucci et al. 2013 | -1.0281 | [-1.7334; -0.3229] | 71.9% | 0.374 | 10.66* |
|  | Monzanil et al., 1993 | -0.9693 | [-1.6431; -0.2954] | 67.1% | 0.32 | 9.12* |
|  | Yin et al., 2013 | -0.9068 | [-1.4548; -0.3588] | 57.7% | 0.179 | 7.09 |
|  | Zhu et al., 2006: SCH | -1.2411 | [-1.7960; -0.6862] | 58.3% | 0.186 | 7.2 |

For the MMSE, removing the results of the Wijsman et al. (2013) study significantly affected heterogeneity: the Q value decreased from 1844.14 to 216.93. This may be due to the large sample of the study - it included 161 people with HT and 4928 controls. A large decrease in Q was also observed when excluding the Park et al. (2010) study, which also had a large sample. Removing both studies also had the largest impact on the SMD. In the case of Wijsman et al., 2013, it decreased to -0.8821, ​​while after removing the Park et al. (2010) study, it increased to -1.2251. However, it should be noted that regardless of the studies excluded from the analysis, the CIs did not cross 0, which indicates statistically significant differences in MMSE test performance between HT patients and healthy/euthyroid controls.

Analyses of the TMT A results indicated that removing the Osterweil et al. (1992) study led to homogeneous results. Interestingly, when these results were omitted, the CIs did not cross 0, indicating a statistically significant difference in performance with SMD = 0.5312 [0.201; 0.862]. In the case of this study, the group differences were the largest, and the results were also presented as *M* and SEM.

For the TMT B, the Kramer et al. (2009) study had the greatest impact on heterogeneity: after removing this study, the Q value decreased from 27.51 to 14.02, while it was still statistically significant (*p* < .05). In addition, the effect size also increased from 0.345 to 0.44, and the CIs did not include 0. It should also be noted that the results of this study were presented in the form of *M* and SEM.

For the DST forward, the results of the Monzani et al. (1993) study had the greatest impact on heterogeneity: after excluding them from the analyses, Q values ​​dropped from 164.97 to 69.28, while still being statistically significant. The effect size also decreased from -1.331 to −0.483. It should be noted that regardless of the excluded study, the CIs always crossed 0, which indicates no statistically significant differences between groups.

For the backward DST, the Osterweil et al. (1992) study had the greatest impact on heterogeneity: after its removal, Q values ​​decreased from 233.29 to 116.07, and the effect size decreased from -1.2438 to -0.7711. In this study, *M* and SEM were reported. The results of the study by Monzani et al. (1993) also had a fairly large impact on heterogeneity. After their removal, Q values ​​decreased to 150.03.

The Jackknife analyses for fluency showed that removing the Kramer et al. (2009) study had the greatest impact on heterogeneity: Q values ​​decreased from 146.41 to 26.64, while still being statistically significant. After removing this study, the effect size also decreased from −0.448 to −0.247, and the CIs still crossed 0. In the case of the Menicucci et al. (2013) study, after its removal, the SMD increased to −0.568, and the CIs did not cross 0. It should be noted that in this study, medians and the IQR were reported. Also, after removing the Park et al. (2010) study, the SMD changed to −0.5867, and the CIs, similar to those in the Menicucci et al. (2013) study, did not cross 0.

For the WMS MQ, the greatest impact on heterogeneity was exerted by the results of the studies Menicucci et al. (2013) and Monzanil et al. (1993). After their removal, the Q values ​​decreased from 48.54 to 25.32 and 21.91, respectively. Both studies also had the greatest impact on SMD. After removing the Menicucci et al. (2013) study, the values ​​increased from −1.285 to −1.56, while after removing the Monzanil et al. (1993) study, they decreased to −0.961.

In the case of the WMS Mental Control subscale, it was noted that omitting the studies by Baldini et al. (1997), Monzanil et al. (1993), Yin et al. (2013) and Zhu et al. (2006) led to homogeneity of the results. For the SMD values, the most significant results were those of Zhu et al. (2006) and Yin et al. (2013). Removing them modified the effect size from −1.075 to −1.241 and −0.907, respectively.
