# Supplementary material for: Prevalence, hormonal correlates, severity, and neural basis of neurocognitive impairment in patients with hypothyroidism: Systematic review and meta‐analyses
Source: Alzheimers Dement. 2025 Nov 26;21(11):e70924. doi: 10.1002/alz.70924 (PMC12657124; doi:10.1002/alz.70924)
Supplement: Supplementary file 9 — Supporting Information [file ALZ-21-e70924-s006.docx]

Supplementary Table 8. Neuroimaging studies

| Authors | | Participants | | Basic sociodemographic data | | Clinical data | Hormonal ranges | | Main findings - neuroimaging | | |
| --- | --- | --- | --- | --- | --- | --- | --- | --- | --- | --- | --- |
| Anjana, et al., 2006 [84]  India | | 30 newly diagnosed HT patients and 30 healthy controls | | NI about % of females; mean age: 32.63 ± 8.59; NI about mean education time | | NI | NI | | | Auditory (ABR, MLR, SVR): significant decrease in amplitude of wave V in HT patients and increase in latency of wave Na of MLR in HT patients | |
| Anjana, et al., 2008 [85]  India | | 26 newly diagnosed primary HT patients and 26 age- and sex-matched controls | | 100% females; mean age 31.85 ± 8.57 years; NI about mean education time | | Baseline: TSH, μIU/ml: 14.56 ± 7.23; fT3, pg/ml: 2.16 ± 0.90; fT4, ng/dl: 0.99 ± 70.52; NI about mean BMI | NI | | | Auditory (oddball paradigm): a significant prolongation of latencies of N100, P200 and  N200 in HT patients.  The latencies of P300Fz and P300Cz were positively correlated with the TSH value, while P200Fz and P300Fz were negatively correlated with fT4. | |
| Jaiswal, et al., 2016 [24]  India | | 36 adult SCH patients and 36 age‑ and sex‑matched euthyroid controls | | 86.1% females; mean age: 35.5 ± 5.9; NI about mean education time | | Mean TSH (μIU/ml) 7.2±2.5; mean FT3 (pg/ml) 2.7±0.7; mean FT4 (ng/dl) 1.1±1.0; mean BMI: 26.3±3.5 | TSH: 0.34–4.24 μIU/ml; FT3: 2.0–4.2 pg/ml; FT4: 0.6–1.7 ng/dl | | | Pattern visual evoked potential: N135 latency period was higher in SCH cases as compared to controls in the right eye  TSH levels were not associated with N75, P100, and N135. | |
| Jensovsky, et al., 2002 [86]  Czech Republic | | 31 patients with SCH and 29 healthy controls | | 90,3% females; mean age: 52 ± 12.5; NI about mean education time | | mean TSH: 8.8±3.7 mU/l; mean fT4: 13.7±2.3 pmol/l; mean fT3 6.8±1.1 pmol/l ; NI about mean BMI | NI | | | Auditory (oddball paradigm): mean P300 latency in SCH patients was significantly higher | |
| Kakked, et al., 2013 [87]  India | | 48 patients with newly diagnosed primary HT (SCH, n=26; and overt HT, n=22) and 20 healthy controls | | SCH: NI about % of females; mean age: 38.05 ± 10.5; NI about mean education time; Overt HT: NI about % of females; mean age: 40.5 ± 11.2; NI about mean education | | SCH: mean TSH: 9.5 ± 3.7; mean fT3: 3.4 ± 0.7; mean fT4: 1.4 ± 0.2; Overt HT: mean TSH: 46.5 ± 24.2; mean FT3: 1.4 ± 0.7; mean fT4: 0.6 ± 0.2 | TSH: 0.4 –4.0 idU/mL; fT3: 1.50 –4.71 pg/mL; fT4: 0.80 –1.90 ng/dL | | | EMG evaluation of the blink reflex: prolonged second ipsilateral response and second contralateral response latencies in HT. | |
| Menicucci, et al., 2013 [39]  Italy | | 17 patients with SCH and 17 euthyroid controls | | 100% females; mean age, 28 ± 4; NI about mean education time | | median (IQR) TSH: 5.3 (1,.9); median (IQR) fT3: 2.7 (0.5); median (IQR) fT4: 10.9 (2.0); NI about mean BMI | TSH: 0.3-3.8 mIU/ml; fT3: 2.1-4.2 pg/ml; fT4: 7.1-18.5 pg/ml | | | The power distribution as a function of the  frequency and of the scalp locations; the power lateralization between hemispheres: during the resting state, SCH had a reduced alpha rhythm in the right hemisphere and bilaterally in the frontal areas. SCH had a general gamma lateralization reduction compared to controls, which were lateralized to the left.  During stimulations, dominant beta2 activity in the parietal areas was lateralized toward the right hemisphere in SCH, while toward the left hemisphere in Controls | |
| Mishra, et al., 2016 [72]  India | | 29 newly diagnosed HT patients and 29 age & education matched controls | | 100% females; mean age: 29.9 ± 6.74; NI about mean education time | | mean TSH (NI about units): 33.23 ± 35.01; mean T3 (NI about units): 0.51 ± 0.48 | NI | | | Auditory (oddball paradigm): the latency of P300 was higher in HT | |
| Mishra, et al., 2018 [73]  India | | 29 newly diagnosed HT patients and 29 age- and education-matched controls | | 100% females; mean age: 24.12 ± 3.07; NI about mean education time | | mean TSH (NI about units): 33.23 ± 35.01; mean T3 (NI about units): 0.51 ± 0.48 | NI | | | Auditory (oddball paradigm): the P300 latency in HT was higher than in controls. | |
| Nazliel, et al., 2002 [88]  Turkey | | 24 SCH and 24 overt HT (newly diagnosed) and 52 age and sex matched controls | | NI about % of females; SCH patients mean age 47 ± 10; overt HT mean age 43 ± 12; NI about mean education time | | SCH patients: mean TSH: 12.9 ± 5.3 μIU/ml; mean fT3 (pg/ml): 2.65 ± 0.40; mean fT4 (ng/dl): 1.08 ± 0.20 NI about mean BMI; Overt HT patients: mean TSH: 89.2 ± 70.0 μIU/ml, mean fT3 (pg/ml): 1.64 ± 0.70; mean fT4 (ng/dl): 0.53 ± 0.20, NI about mean BMI | TSH: 0.35-5.5 μIU/ml; FT3: 2.30-4.20; fT4: 0.80-1.90 ng/dl | | | Pattern-shift visual evoked potentials: no statistically significant differences between controls and HT groups in mean P100 latency and mean P100 amplitude;  No statistically significant relationships between latency and amplitudes of PVEP and fT3, fT4 and TSH values. | |
| Oerbeck, et al., 2007 [89]  Norway | | 12 young adults with an early treated severe form of congenital HT and 12 siblings matched by age at assessment, gender or education. | | 50% females; mean age 20.3 ± 0.9; mean time of education: 11.8 ± 1.0 | | mean TSH: 35.3 (38.8) mU/L; mean fT3: 13.8 ± 5.6 pmol/L; NI about mean fT4 and BMI | NI | | | Auditory (oddball paradigm): delayed latency on the early cognitive EP components P100 and N100, and significant amplitude differences in the form of higher P100 and smaller N100 values. Higher amplitude than controls at posterior sites represented by Pz, but lower amplitude at frontal sites represented by Fz. | |
| Osterweil, et al., 1992 [33]  USA. | | 54 non-demented HT patients and 30 euthyroid controls matched by age, sex and education | | 46% females; mean age 68.6 ± 16.4; mean education time: 11.2 ± 3.6 | | mean TSH 66.3 ± 55.4 pu/mL; mean T3: 68.5 ± 41.2 ng/dL; mean T4 2.3 ± 2.2; NI about mean BMI | TSH: 0.3-5.7 pu/mL; T4 4.2-11.0 pg/dL; T3 70-160 ng/dL | | | Auditory and visual EP: longer latencies than controls on the 20-minute check size (patterned visual-evoked potential).  No correlation between serum TSH, and electrophysiological test measures. | |
| Ozata, et al., 1997 [90]  Turkey | | 14 patients with newly diagnosed primary HT and 30 sex-, age-, and education-matched controls | | 64,3% females; mean age: 26.71 ± 2.39; mean education time: 7.9 ± 2.1 | | mean TSH: 58.1 ± 12.2 μIU/mL; mean fT3: 1.42 ± 0.30 pg/mL; mean fT4: 0.41 ± 0.19; NI about mean BMI | TSH < 6.5 μIU/mL; fT3: 2.2 to 4.7 pg/mL; fT4: 0.85 to 2.67 ng/dL | | | Auditory discrimination task paradigm: HT showed a significant lengthening of the P300 and N100 wave latencies.  No correlation was found between electrophysiologic variables and fT3, fT4, or thyrotropin levels. | |
| Paladugu, et al., 2015 [91]  India | | 45 new onset HT (24: overt HT; 21: SCH) and  33 age and sex matched controls | | 95,8% females (overt HT); 95,3% (SCH); mean age 26 ± 5.8 (overt HT), 25 ± 6.4 (SCH); NI about mean education time | | NI about the units; mean TSH: 94.1 ± 15.2 (overt HT) 12.3 ± 2.4 (SCH); mean T3: 0.71 ± 0.25 (overt HT), 0.99 ± 0.31 (SCH); mean T4: 3.36 ± 0.90 (overt HT), 7.03 ± 1.67 (SCH); mean BMI: 29.9 ± 4.1 (overt HT), 28.9 ± 4.4 (SCH) | NI | | | Auditory (oddball paradigm): mean P300 latencies were higher in HT and SCH than in controls; | |
| Rizzo, et al., 2008 [92]  Italy | | 10 patients with overt HT (Hashimoto's thyroiditis, n=9 and radioiodine therapy, n=1 for hyperthyroidism treatment) and 10 age-matched healthy caregivers | | 60% female; mean age: 53 ± 8 years; NI about education | | TSH (mU/L): 24.23 ± 4.98; fT3 (pmol/L): 2.9 ± 0.35; fT4 (pmol/L): 7.72 ± 1.71; NI about BMI | NI | | | EMG recorded from right first dorsal interosseus muscle (RMT, AMT, CSP, ICF, PSP, SICI): RMT and AMT were significantly higher in HT than in controls.  Motor evoked potential amplitudes obtained with higher stimulus intensities were significantly lower in HT. The duration of CSP was significantly longer in patients than in controls. SICI was reduced in patients compared to controls | |
| Sharma, et al., 2014 [93]  India | | 75 newly diagnosed patients with SCH and 75 newly diagnosed patients with clinical HT and 75 healthy age and sex matched controls | | 94,7% females (SCH), 97,3% females (HT); mean age: 36±11 (SCH), 36±9 (HT); NI about mean time of education | | TSH (mIU/L) 8.98 ± 2.55(SCH), 99.84 ± 57.1 (HT); mean T4 (nmol/L): 103.14 ± 13.1 (SCH), 29.43 ± 16.23 (HT); NI about mean fT3, fT4 and BMI | SCH: TSH level>5.1mIU/L and T4≥57.9 nmol/L. For HT: TSH level>5.1mIU/L and T4<57.9 nmol/L. | | | Auditory (oddball paradigm): HT patients showed a significant increase in P300 latency compared to control and SCH. .  Negative correlation between P300 latency and T4, positive correlation between P300 latency and TSH. | |
| Waliszewska-Prosół, et al., 2021 [94]  Poland | | 68 patients treated with LT4 in the euthyreosis phase and 45 age and sex-matched controls | | 86,8% females; mean age: 44.3; mean education 11.5 | | mean TSH (UIU/ml): 1.94 ± 1.08; mean fT3 (PG/ml): 2.94 ± 0.40; mean fT4 (NG/dl): 1.03 ± 0.16; mean BMI: 24.6 ± 3.1 | TSH: 0.35–5.6 UIU/ml; fT3: 2.5–3.9 pg/ml; fT4: 0.61–1.12 ng/dl | | | Auditory (oddball paradigm): the mean values for the latency of N200 and P300 potentials recorded from all electrodes were significantly longer in HT than in the control group, while the mean amplitude of P300 potentials - lower.  There were no statistically significant differences between EP parameters in patients with different initial diagnosis—HT and SCH.  There were no statistically significant correlations between the mean EP parameters, and thyroid hormones levels | |
| Diffusion tensor imaging | | | | | | | | | | | |
| Cao et al.  2023 [57]  China  [DTI] | 31 drug-naïve HT and 28 healthy controls | | 71% female; mean age 41.32±9.14, mean education time: 13.55± 3.70 | | Mean TSH (mlU/l) 61.48± 31.85; mean fT4 (pmol/l): 6.51± 2.22; Mean fT3 (pmol/l) 3.95± 3.21; NI about mean BMI | | | TSH: 0.35 – 4.94 mIU/L; fT3: 2.43–6.01pmol/L, 9.01–19.05; fT4: pmol/L | | | Mean fractional anisotropy values in the right anterior thalamic radiations, left cingulum cingulate, inferior longitudinal fasciculus, arcuate fasciculus, anterior forceps of the corpus callosum, and bilateral corticospinal tract were decreased in HT. |
| Gunbey, et al. 2021 [95]  Turkey  [DTI] | 18 newly diagnosed and not previously treated patients with HT and 14 healthy controls matched by age and sex | | 78% women; mean age 40 ± 8.4 | | Mean fT3: 0.28 ± 0.45 pg/ml; mean fT4: 0.76 ± 0.54 ng/dl; mean TSH: 72.3 ± 25.4 μIU/ml | | | NI | | | Extensive reductions of FA in the supratentorial white matter including right- corticospinal tract, right posterior limb of the internal capsule, left uncinate fasciculus, left inferior longitudinal fasciculus.  In the ROI analyses right superior longitudinal fasciculus showed significantly radial diffusivity increment in patients with HT. Significant axial diffusivity decrement revealed ROI analyses of left cingulum, right external capsules, left posterior limb of the internal capsule and body of corpus callosum in patients with HT. The ROI analyses showed significant difference in terms of R hippocampal fractional anisotropy, right cingulum, body of corpus callosum, and splenium of corpus callosum mean diffusivity, splenium of corpus callosum axial diffusivity, right cingulum, right superior longitudinal fasciculus, body of corpus callosum, and splenium of corpus callosum radial diffusivity.  BDI; Right and left cingulum fractional anisotropy was negatively associated with depressive symptoms |
| Singh et al. 2014 [80]  India  [DTI] | 8 newly diagnosed HT patients with elevated TSH and low fT4 levels and 8 healthy controls | | mean age 32.8 + 9.22; 62.5% females; Education M=11.3±5.55 | | Mean fT4: 6.68 ± 2.20 pmol/l; mean TSH 51.28 ± 36.05 μIU/ml; NI about mean fT3 and BMI | | | NI | | | Significantly decreased fractional anisotropy values were observed in HT patients in bilateral uncinate fasiculus, bilateral corticospinal tracts, fornix and left inferior longitudinal fasciculus compared with healthy controls. Significantly increased mean diffusivity values were observed in right inferior fronto-occipital fasciculus, bilateral superior longitudinal fasciculus, left inferior longitudinal fasciculus, right anterior thalamic radiations, right arcuate fasciculus, right superior thalamic radiation, left cingulum, bilateral uncinate fasciculus and right arcuate fasciculus fibres in HT patients compared with controls. Significantly increased mean diffusivity values were observed in right inferior fronto-occipital fasciculus, bilateral superior longitudinal fasciculus, left inferior longitudinal fasciculus, right anterior thalamic radiations, right arcuate fasciculus, right superior thalamic radiation, left cingulum, bilateral uncinate fasciculus and right arcuate fasciculus fibres in HT patients compared with controls. |
| Positron emission tomography | | | | | | | | | | | |
| Bauer et al. (2009) [96]  USA  [PET] | 13 previously untreated HT patients (4 clinical HT and 9  SCH) and 10 age- and gender-matched healthy controls | | NI about percentage of female, mean age nor mean education time | | Mean TSH: 16.9 μIU/ml; NI about mean fT3 and fT4 | | | NI | | | HT patients had lower relative regional activity in bilateral amygdala, hippocampus, perigenual anterior cingulate cortex, left subgenual anterior cingulate cortex, and right posterior cingulate cortex  BDI, HAM-D: negative covariation between depressive symptoms and activity in the bilateral middle frontal gyrus, right subgenual, and dorsal anterior cingulate cortex |
| Wu et al. (2021) [97]  China  [PET] | 23 LT4 withdrawal group and 15 LT4 replacement group papillary thyroid cancer patients and 34 healthy subjects | | 73.91% females; mean age: 44.1 (11.3); mean education 11.0 (4.7) | | mean TSH (UIU/ml): 123.0 ± 49.0; mean fT3 (pmol/L): 2.2 ± 1.1; mean fT4 (pmol/L): 1.8 ± 2.3; mean BMI: 24.0 ± 3.6 | | | NI | | | Withdrawal vs LT4: decreased 18F-FDG uptake in the right middle occipital gyrus and the left postcentral gyrus. The rCMRglu significantly increased in the left superior frontal gyrus, orbital part.  Withdrawal vs healthy: hyperactivity in the right hippocampus and the left inferior temporal gyrus; rCMRglu demonstrated decreased metabolism in the left postcentral gyrus and left precuneus. |
| Single photon emission computed tomography | | | | | | | | | | | |
| Kaya et al., 2007 [98]  Turkey  [SPECT] | 20 patients with HT and 12 controls | | 85% females; mean age: 41.1 ± 8.9; NI about mean education time | | Mean TSH (IU/ml): 64 ± 17; mean fT3 (pg/ml): 2.50 ± 1.00; mean fT4 (ng/dl): 0.70 ± 0.70 | | | NI | | | The areas in which significant reduced rCBF were 1) the right hemisphere: superior frontal, inferior frontal, anterior temporal, precentral gyrus, postcentral gyrus, and parietal cortex; 2) in the left hemisphere: superior frontal, inferior frontal, caudate nucleus, and parietal cortex. |
| Krausz et al. 2004 [44]  Israel  [SPECT] | 10 patients with newly diagnosed adult-onset HT due to atrophic or Hashimoto’s thyroiditis and 10 healthy controls | | 100% female; mean age, 45.9 ± 15.1; NI about education time | | mean TSH: 15.1 ± 2.9 mU/L; mean fT4: 9.3 ± 1.6 pmol/L, mean total T3: 2.4±1.0 nmol/L | | | NI | | | Significantly lower rCBF in the HT patients in posterior parts of the brain, including parts of the parietooccipital cortex and temporal lobes.  In the HT group, no regions of significant correlation were observed between rCBF and TSH, and HAM-D. |
| Nagamachi et al., 2004 [99] Japan [SPECT] | 24 patients who had undergone total thyroidectomy  due to thyroid cancer free of thyroid hormone replacement therapy at least 3  weeks and 15 controls | | 75% females; mean age: 55.4; NI about mean education time | | Mean TSH: 120.1 ± 86.2 (μlU/ml); mean T3: 0.33 ± 0.28 ng/ml; mean T4: 2.0 ± 2.9 μg/dl | | | NI | | | Decrease in rCBF in the parietal (inferior parietal gyrus and precuneus) and the occipital lobes (cuneus)  SDS; Within the HT subjects, those with severe and moderate depression, showed significantly lower rCBF of the global, bilateral parietal, and bilateral occipital lobes than did those in the control group. Significant rCBF reduction in the prefrontal area in the severely depressed patients |
| Magnetic resonance spectroscopy | | | | | | | | | | | |
| Bladowska et al., 2019 [100]  Poland  [MRS] | 55 HT patients and 30 age- and gender-matched healthy controls | | 90.91% females; mean age: 43.5; NI about mean education time | | Mean TSH: 1.87 ± 1.24 UIU/ml; mean fT3 2.92 ± 0.54 pg/ml; mean fT4 1.07 ± 0.27 ng/dl | | | TSH: 0.35–5.6 UIU/ml; fT3: 2.5–3.9 pg/ml; fT4: 0.61–1.12 ng/dl | | | Decrease of the N-acetylaspartate/ total creatine ratios in both posterior cingulate gyrus and parietal white matter regions.  N-acetylaspartate/ total creatine ratios in the posterior cingulate gyrus region as well as in the parietal white matter area showed significant positive correlations with fT3 concentrations |
| Kumar et al. 2025 [66]  India  [MRS] | 25 drug naïve HT patients and 30 age-matched controls | | 84% females; mean age: 31.4 (10.54); mean education time: 12.37 (3.54) | | mean TSH 158.37 ± 137.42 μIU mL-1, NI about mean fT3 and fT4; mean BMI 24.75 ± 4.85 | | | NI | | | Increased choline concentration in dorsolateral prefrontal cortex and posterior parietal cortex in HT patients. Choline levels were positively associated with TSH and negatively with T4 in the posterior parietal cortex region. Choline levels were also negatively associated with T4 in dorsolateral prefrontal cortex. Negative correlations were also observed between choline and delay recall memory, immediate recall of semantic word pairs memory, visual retention memory (posterior parietal cortex) and MMSE (dorsolateral prefrontal cortex). |
| Liu et al. 2020 [69]  China  [MRS] | 18 newly diagnosed HT patients with elevated serum TSH and lowered fT4 and fT3 and 18 age-, weight- and sex-matched healthy controls | | 72,2% female; mean age: 43.06 ± 7.04; mean education: 13.22 ± 1.52 | | Mean TSH (mIU/l): 95.3 ± 34.51; mean fT3 (pmol/l): 1.98 ± 0.63; mean fT4 (pmol/l): 5.96 ± 1.59; mean BMI: 23.72 ± 1.02 | | | TSH: 0.55–4.78 mIU/L; fT3: 3.5–6.5 pmol/L; fT4: 11.5–22.7 pmol/L | | | In the medial prefrontal cortex, patients with HT demonstrated significantly decreased GABA+ levels compared to healthy controls.  BDI-II; The median prefrontal cortex GABA+ concentrations in patients with HT were negatively correlated with depressive symptoms |
| Singh et al. 2016 [101]  India  [MRS] | 15 newly diagnosed HT patients with spontaneous HT with elevated TSH and low fT4 levels and 15 age-matched controls | | 50% females; mean age 27.9 ± 6.05; mean education 11.3 ± 3.68 | | mean TSH (lIU/ml): 46.55 ± 34.26; mean fT4 (pmol/l): 5.91 ± 4.09; NI about mean fT3 and BMI | | | TSH = 0.27–4.2 lIU/ml; fT4 = 12.0–22.0 pmol/l | | | Changes at metabolite level in the hippocampus of HT patients compared to healthy controls: significantly reduced glutamate/creatine and myo-inositol/creatine ratios in the hippocampus. |
| Waliszewska-Prosół et al. 2021 [94]  Poland  [MRS] | 68 HT patients treated with LT4 in the euthyreosis phase and 45 age and sex-matched healthy volunteers | | 86,8% females; mean age: 44.3; mean education 11.5 | | mean TSH (UIU/ml): 1.94 ± 1.08; mean fT3 (PG/ml): 2.94 ± 0.40; mean fT4 (NG/dl): 1.03 ± 0.16; mean BMI: 24.6 ± 3.1 | | | TSH: 0.35–5.6 UIU/ml; fT3: 2.5–3.9 pg/ml; fT4: 0.61–1.12 ng/dl | | | Positive correlation between the myo-inositol/creatine ratio in the posterior cingulate gyrus area and P300 latencies. N-acetylaspartate/creatine ratio in the posterior cingulate gyrus region showed significant negative correlations with all N200 latencies. |
| Zhang et al., 2015 [102]  China  [MRS] | 18 newly diagnosed and untreated HT patients and 18 controls | | 72.22% females; mean age: 34.3 ± 9.3; NI about mean education time | | mean TSH (mIU/ml): 86.03 ± 11.42; mean fT3 (pmol/l): 2.00 ± 0.78; mean fT4 (pmol/l): 5.39 ± 2.13; NI about mean BMI | | | NI | | | The increase in concentrations of glutamate and choline in the posterior cingulate cortex in patients with HT relative to controls. |
| Magnetic resonance imaging and functional magnetic resonance imaging | | | | | | | | | | | |
| Chambers et al. 2021 [103]  UK  [MRI] | 419 patients with diagnosis of HT (UK Biobank) and 18287 controls (UK Biobannk) | | 80.7% female; mean age 64.5 ± 7.32, n.i. about education | | Mean BMI 28.0 ± 4.92; NI about mean TSH, fT3 and fT4 | | | NI | | | Significant reductions in bilateral total cerebellar and pallidum volumes and across most cerebellar lobules, aside from superior posterior vermal regions (VI-VIIIa vermis). |
| Cooke et al. 2014 [104]  Ireland  [MRI] | 11 untreated patients with HT and 9 healthy control subjects matched by age, sex and education | | 82% female; mean age 42.3 ± 3.5 years; NI about education | | Mean TSH: 61.85 ± 8.54 mU/L ; mean fT4: 5.19 ± 0.70 pmol/L | | | fT4 levels below the lower limit of the normal range (11 pmol/L) and TSH levels above the upper limit of the normal range (10 mU/L). | | | Significant decrease in volume within the right hippocampus |
| Leyhe et al. 2013 [46]  Germany  [MRI] | 18 Hashimoto thyroiditis patients on stable LT4 treatment, and a serum TSH concentration of 0.4–2.5 mU/l. and 12 controls; on LT4 treatment for goitre or after thyroid surgery; matched by gender, age, education, and task performance in the d2 test (as assessed 6–10 weeks before magnetic resonance imaging) | | 84,6% females; mean age 43 ± 12; mean education 13 ± 4 | | mean TSH (mU/l) 1.5 ± 1.0; mean fT4 (pmol/l) 15.5 ± 2.9; mean fT3 (pmol/l) 4.8 ± 0.6; mean BMI 25.8 ± 5.1 | | | TSH, 0.4–2.5 mU/l; free T4, 12–23 pmol/l; free T3, 3.5–6.5 pmol/l; TPOAbs,<100 IU/l; Tg-Abs,<100 IU/l. | | | Overall grey matter density was similar for both Hashimoto’s thyroiditis patients and control group. |
| He et al. 2011 [62]  China  [MRI/fMRI] | 13 female patients before having been treated with LT4 and 12 euthyroid controls matched by age and education | | 100% female, mean age 29.4 ± 6.3; mean education time 12.0 ± 3.2 | | Mean TSH (mIU/l) 102.6 ± 57.8; mean fT3 (pmol/l) 2.3 ± 0.9; mean fT4 (pmol/l) 7.2± 1.9; NI about mean BMI | | | 3.5–6.5 pmol/l for fT3, 11.5–22.7 pmol/l for fT4, and 0.35–5.5 mIU/l for TSH. | | | Significant differences of the magnitude of BR–FR contrast of task induced deactivation between HT and controls were found in the bilateral medial prefrontal cortex, posterior cingulate cortex, and left inferior partial lobule. |
| Kumar et al. 2018 [45]  India  [MRI/fMRI] | 28 drug naive SCH patients and 28 healthy controls matched by age and education | | NI about participant's sex; mean age 31.53 ± 8.10; mean education 12.96 ± 3.08 | | mean TSH: 9.61 ± 1.39 μIU mL-1, NI about mean fT3 and fT4; mean BMI 24.71 ± 4.83 | | | NI | | | A significantly decreased intrinsic functional connectivity was obtained in the anterior cingulate gyrus, paracingulate gyrus and juxtapositional lobule cortex, precentral gyrus, anterior cingulate gyrus and posterior cingulate gyrus. Increased intrinsic functional connectivity was obtained in the frontal pole, superior fontal gyrus, inferior frontal gyrus, supramarginal gyrus, angular gyrus, frontal orbital cortex. A significant reduction in extrinsic network functional connectivity was obtained between default mode network and right frontoparietal attention network, somato-motor network and posterior default mode network. An increased extrinsic-network functional connectivity was also found between somato-motor network and anterior default mode network. |
| Quinque et al. 2014 [47]  Germany  [MRI/fMRI] | 18 patients with LT4 treated HT and 18 healthy controls matched for age, sex, intelligence and TSH | | 88,9% females; mean age 32 (9.6); NI about education | | mean TSH (mU/l) 2.0 ± 1.1; mean fT3 (pmol/l) 4.3 ± 0.6; mean fT4 (pmol/l) 18 ± 1.6; NI about mean BMI | | | NI | | | TSH was correlated with reduced grey matter density in left anterior cingulate cortex and TPO-ab with increased grey matter density in right amygdala. Correlation between fT4 and reduced connectivity strength between left amygdala and right middle temporal gyrus as well as between subcallosal cortex and right frontal pole. Anti-TPO levels were correlated with increased connectivity between subcallosal cortex and left parahippocampal gyrus.  BDI; Positive correlation between BDI results and grey matter density in right postcentral gyrus, left superior frontal gyrus, and left cuneus Negative correlation between depressive symptoms severity and grey matter density in left middle temporal gyrus. |
| Singh et al. 2013 [105]  India  [MRI] | 10 patients diagnosed with HT for the first time and not treated previously and 10 healthy controls matched by age | | 70% female; mean age 31.1 ± 6.90; NI about education | | mean TSH (lIU/ml) 123.66 ± 23.42; NI about mean fT3; mean FT4 (pM) 5.69 ± 2.63; mean BMI 22.28 ± 2.28 | | | fT3 = 2.8–7.1 pM, fT4 = 12.0–22.0 pM and TSH = 0.27–4.2 lIU/ml | | | Significant reduction in grey matter volume bilaterally in the cerebellum and left postcentral gyrus; significant reduction in white matter volume was found bilaterally in the cerebellum, right precentral gyrus, right inferior and middle frontal gyrus, right inferior occipital gyrus and right inferior temporal gyrus |
| Su et al. 2023 [36]  China  [MRI/fMRI] | 44 newly diagnosed patients with HT and 54 controls matched by age, sex, and education level | | 77.3% female; mean age 39.30 ± 9.80; mean education 13.57 ± 3.45 | | median (IQR) TSH: 44.81 ± 55.51 mIU/L; mean fT3, pmol/L, 3.30 ± 1.18; mean fT4, pmol/L: 6.81 ± 2.22; NI about mean BMI | | | TSH: 0.35–4.94 mIU/L; fT4: 9.01–19.05 pmol/L | | | Reduced gray matter volumes in the left middle frontal gyrus, left dorsolateral superior frontal gyrus, left supplementary motor area, orbital part of the right superior frontal gyrus, and right superior temporal gyrus. Increased gray matter volumes in the bilateral cerebellar Crus I and left precentral gyrus. Increased functional connectivity between the right cerebellar Crus I and left precentral gyrus, triangular part of the inferior frontal gyrus, and angular gyrus of the inferior parietal lobe. |
| Yin et al. 2013 [82]  China  [MRI/fMRI] | 16 patients with SCH treated with LT4 and 16 healthy volunteers matched for sex, age and years of education | | 100% females; mean age: 34 ± 88; mean education level 11 ± 2 | | mean TSH (mIU/ml): 19.43 ± 9.25; mean fT3 (pmol/l): 4.01±0.27; mean fT4 (pmol/l): 8.57±0.91; NI about BMI | | | TSH: 0.34–5.6 mIU/ml; fT3: 3.8–6.0 pmol/l; fT4: 7.86–14.41 pmol/l | | | In euthyroid subjects bilateral dorsolateral prefrontal cortex, bilateral premotor area, supplementary motor area/anterior cingulate cortex, bilateral parietal lobe and right caudate nucleus/thalamus showed load effect, while in patients before treatment only left dorsolateral prefrontal cortex, left parietal lobe, bilateral premotor area and right caudate nucleus/thalamus. |
| Yin et al. 2021 [50]  China  [MRI/fMRI] | 18 patients with SCH and elevated serum TSH levels and 18 healthy controls matched for age and education | | NI about % of females; mean age: 31 ± 6; mean education: 11 ± 2 | | mean TSH (mIU/L): 9.32 ± 2.43; mean fT3 (pmol/L): 4.46 ± 0.63; mean fT4 (pmol/L): 9.72 ± 1.25; NI about BMI | | | fT3 = 3.6–5.7 pmol/L; fT4 = 9.1–15.4 pmol/L; TSH = 0.51–4.85 mIU/L. | | | Normalized gray matter volumes of patients with SCH were significantly lower in the bilateral prefrontal cortex (including middle, medial, and inferior frontal gyri), cingulate gyrus, precuneus, left middle temporal gyrus, and insula. For euthyroid subjects, more brain areas were activated in the color-naming task, and their activated intensities were higher than those in performing the word-reading task: patents with SCH had lower activation in the prefrontal cortex (mainly dorsolateral prefrontal cortex and ventrolateral prefrontal cortex), parietal lobe (precuneus and inferior parietal lobe), cingulate cortex (anterior cingulate cortexand/or posterior cingulate cortex), thalamus, and superior/middle temporal cortex.  Regional volumes and percentage of BOLD signal changes in the prefrontal cortex, anterior cingulate cortex, and precuneus were negatively correlated with the TSH level. There was a significant negative correlation between the TSH levels and the activation intensity of prefrontal cortex, anterior cingulate cortex, and precuneus. |
| Zhu et al. 2006 [83]  China  [MRI/fMRI] | 9 HT patients and 11 SCH patients first diagnosed not treated with medicine or surgery and 12 euthyroid control subjects | | HT: 100% females; mean age: 31.44 ± 9.29; mean education 5.67 ± 3.24; SCH: 90,9% females; mean age: 30.55 ± 9.68; mean education 9.09±4.61 | | HT: mean TSH (mIU/ml): 44.68 ± 19.60; mean fT3 (pmol/l): 1.98 ± 0.53; mean fT4 (pmol/l): 5.91±2.02; SCH: mean TSH (mIU/ml): 14.67 ± 7.13; mean fT3 (pmol/l): 3.05 ± 0.45; mean fT4 (pmol/l): 13.01 ± 3.27. NI about mean BMI | | | TSH (mIU/l): 0.4–4.0; fT3 (pmol/l): 2.3–6.3; fT4 (pmol/l): 8.4–29.6 | | | In the group of SCH patients, the load effect of BOLD response was only found in the parietal areas and premotor areas |

ABR - Auditory Brainstem Response; AMT - Active motor threshold; anti-TPO - Thyroid Peroxidase Antibodies; BDI - Beck Depression Inventory; BMI – Body Mass Index; CSP - Cortical Silent Period; DTI - Diffusion tensor imaging; EMG – Electromyography; EP - evoked potentials; FA - fractional anisotropy; fMRI - Functional magnetic resonance imaging; fT3 – free triiodothyronine; fT4 - free thyroxine; HAM-D - Hamilton Rating Scale for Depression; HT – hypothyroidism; ICF - Intracortical facilitation; LT4 – L-thyroxine; MLR - Mid Latency Response; MRI - Magnetic resonance imaging; MRS - Magnetic resonance spectroscopy; NI – no information; PET - Positron emission tomography; PSP - Peripheral silent period; rCBF - Regional cerebral blood flow; RMT - Resting motor threshold; ROI - Region of interest; SCH – subclinical hypothyroidism; SICI - Short interval cortical inhibition; SPECT - Single photon emission computed tomography; SVR - Slow Vertex Response; T3 – triiodothyronine; T4 – thyroxine; TSH - thyroid-stimulating hormone
