# Supplementary material for: Prevalence, hormonal correlates, severity, and neural basis of neurocognitive impairment in patients with hypothyroidism: Systematic review and meta‐analyses
Source: Alzheimers Dement. 2025 Nov 26;21(11):e70924. doi: 10.1002/alz.70924 (PMC12657124; doi:10.1002/alz.70924)
Supplement: Supplementary file 10 — Supporting Information [file ALZ-21-e70924-s009.docx]

Supplementary Table 9. Auditory evoked potentials: jackknife analyses

| Tool | Studies omitted | SMDs | 95%-CI | *I^2^* | *Tau*^2^ | *Q* |
| --- | --- | --- | --- | --- | --- | --- |
| N200 latency | All studies included | 0.912 | [0.638; 1.186] | 52.7% | 0.071 | 12.68* |
|  | Anjana et al., 2008 | 0.9446 | [0.4584; 1.4307] | 71.7% | 0.19 | 10.62* |
|  | Ozata et al., 1997 | 1.0499 | [0.8590; 1.2409] | 0% | 0 | 4.01 |
|  | Waliszewska-Prosół, et al. 2021 | 0.6346 | [0.2725; 0.9967] | 37.4% | 0.048 | 4.79 |
| P 300 latency | All studies included | 1.1243 | [0.7464; 1.5023] | 87.1% | 0.518 | 116.27*** |
|  | Anjana et al., 2008 | 1.3115 | [0.9110; 1.7119] | 87.4$ | 0.466 | 95.42*** |
|  | Jensovsky et al., 2002 | 1.1395 | [0.7353; 1.5437] | 88% | 0.561 | 116.24*** |
|  | Mishra et al., 2016 | 1.0305 | [0.6793; 1.3817] | 85.3% | 0.409 | 95.15*** |
|  | Mishra et al., 2018 | 1.0305 | [0.6793; 1.3817] | 85.3% | 0.409 | 95.15*** |
|  | Ozata et al., 1997 | 1.1236 | [0.7205; 1.5266] | 87.9% | 0.56 | 115.96*** |
|  | Paladugu et al., 2015 Overt HT | 1.0081 | [0.6179; 1.3982] | 86.5% | 0.482 | 96.07*** |
|  | Paladugu et al., 2015 SCH | 1.1936 | [0.7702; 1.6170] | 88.5% | 0.577 | 113.45*** |
|  | Sharma et al., 2014 SCH | 1.1988 | [0.8256; 1.5720] | 82.8% | 0.464 | 81.53*** |
|  | Sharma et al., 2014 HT | 1.1322 | [0.7252; 1.5392] | 87.9% | 0.567 | 116.09*** |
|  | Waliszewska-Prosół, et al. 2021 | 1.0952 | [0.6210; 1.5693] | 88.9% | 0.676 | 108.1*** |
| P300 amplitude | All studies included | -0.3434 | [-0.5267; -0.1601] | 43% | 0.04 | 17.54 |
|  | Anjana et al., 2008 | -0.3366 | [-0.5817; -0.0915] | 59.7% | 0.072 | 17.36* |
|  | Ozata et al., 1997 | -0.4420 | [-0.6248; -0.2592] | 31.9% | 0.025 | 10.27 |
|  | Sharma et al., 2014 SCH | -0.3874 | [-0.5753; -0.1996] | 36.4% | 0.032 | 14.15 |
|  | Sharma et al., 2014 HT | -0.3314 | [-0.5423; -0.1204] | 48.6% | 0.054 | 17.52* |
|  | Waliszewska-Prosół, et al. 2021 | -0.1898 | [-0.3546; -0.0249] | 0% | 0 | 4.96 |

For N200 latency, two studies had the greatest impact on the obtained results: after removing the results of Ozata et al. (1997), an increase in the effect size was observed, while the results were homogeneous: I^2^ values ​​dropped to 0% and Q = 4.01. On the other hand, removing Waliszewska-Prosół, et al. (2021) significantly reduced the effect size, SMD = 0.635, and also led to homogeneity of the results, I^2^ = 37.4%; Q = 4.79.

Visual inspection of the results of studies assessing P300 latency showed that removing consecutive studies did not have a large impact on either the effect size or heterogeneity. The SMD value ranged from 1.008 (after removing Paladugu et al., 2015, the overt HT group) to 1.312 (after removing Anjana et al., 2008). In turn, Q values ​​ranged from 81.53 (after removing Sharma et al., 2014, the SCH group) to 116.24 (after removing Jensovsky et al., 2002).

The results of the meta-analysis of P300 amplitude were most influenced by Waliszewska-Prosół, et al. (2021): after its removal, the SMD decreased to -0.19, while the heterogeneity assessed by I^2^ decreased to 0% and Q to 4.96. On the other hand, removing Ozata et al. (1997) slightly increased the SMD to -0.4420, while slightly reducing the statistics determining heterogeneity (I^2^ = 31.9%; Q = 10.27).
