# Supplementary material for: Prevalence, hormonal correlates, severity, and neural basis of neurocognitive impairment in patients with hypothyroidism: Systematic review and meta‐analyses
Source: Alzheimers Dement. 2025 Nov 26;21(11):e70924. doi: 10.1002/alz.70924 (PMC12657124; doi:10.1002/alz.70924)

*Supplementary File 1.* *Severity of neurocognitive impairment: TMT A & B, DST and verbal fluency*

*S.F. 2.1. Trail-Making Test A*

In the case of the TMT A, it was possible to identify six comparisons in which the SMD was 1.11 [−0.289; 2.506] (Figure S.F. 2.1.1; see Figures S.F. 2.1.2 and 2.1.3 for Baujat and funnel plots). This result was heterogeneous (Q = 62.11, *p* < .0001) and included 0 in the range.

Figure S.F. 2.1.1 Severity of neurocognitive impairment assessed with TMT-A: forest plot


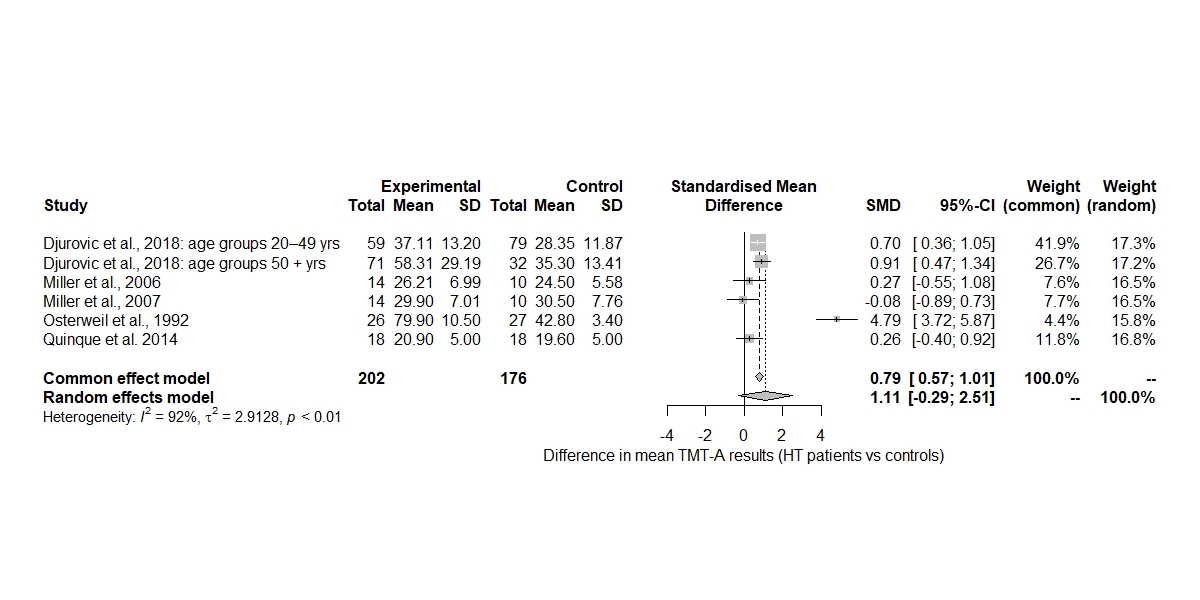


Figure S.F. 2.1.2 TMT A: Baujat plot


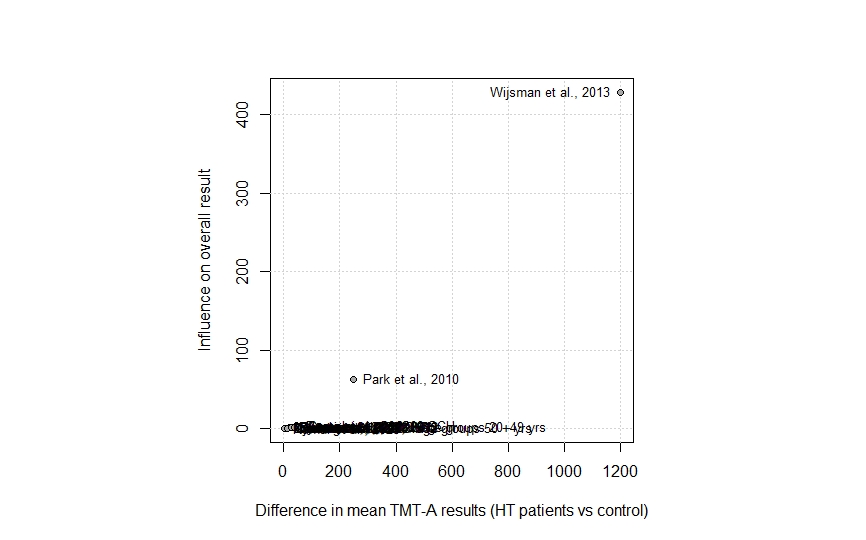


Figure S.F. 2.1.3. TMT A: funnel plot


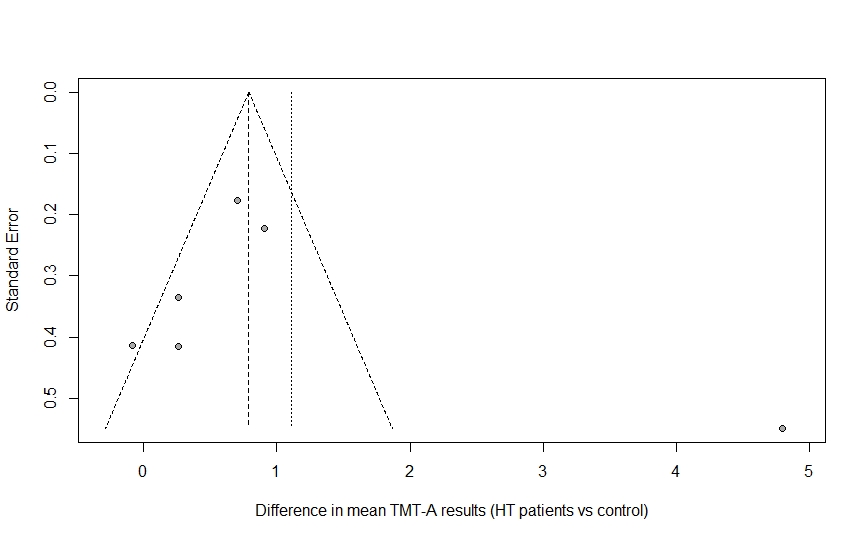


Due to the small number of reported values ​​of potential moderators, the female ratio, mean years of education, mean fT3 and fT4, levothyroxine doses and treatment duration, as well as BMI were not included in the calculations. The mean sample age, was statistically significant moderator, QM (1) = 5.442, *p* = 0.02. The higher the average age, the larger the difference in TMT A completion times (see Figure S.F. 2.4 for the bubble plot). However, after taking this moderator into account, the TMT A results were still heterogeneous, Q (4) = 43.925, *p* < .001.

Figure S.F. 2.1.4. TMT A and mean age: bubble plot


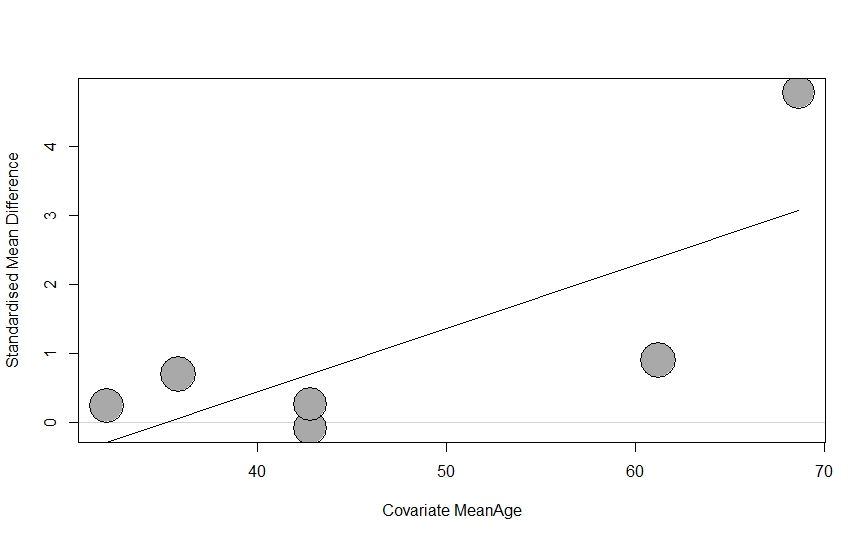


The mean TSH level, was also statistically significant moderator, QM (1) = 4.739, *p* = .03. The higher the mean TSH level, the larger the difference in TMT A completion times (see Figure S.F. 2.5 for the bubble plot). After taking this moderator into account, the TMT A results were still heterogeneous, Q (4) = 41.725, *p* < 0.001.

Figure S.F. 2.1.5. TMT A and mean TSH levels: bubble plot


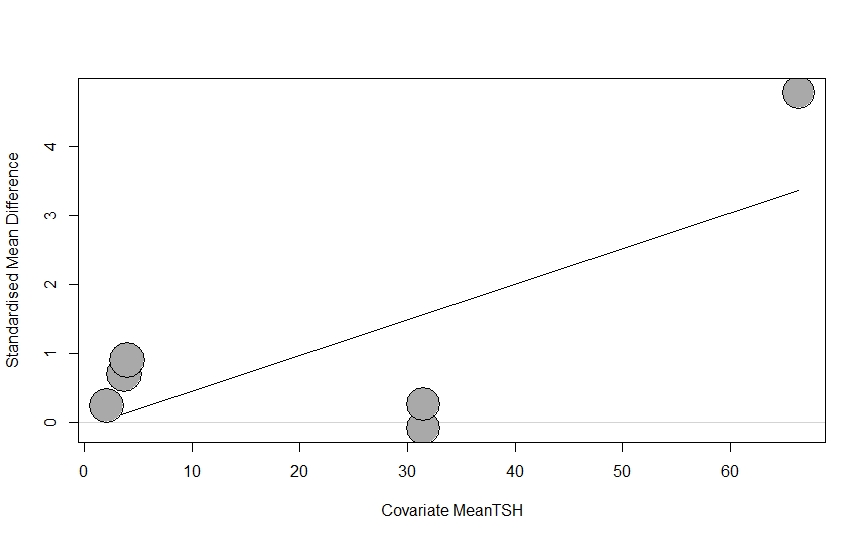


Quality of studies included in the meta-analysis was not statistically significant moderator, QM (1) = 0.423, *p* = .52.

*S.F. 2.2. Trail-Making Test B*

Next, we analyzed the results of the TMT B, for which seven comparisons were identified. The meta-analysis of all the reported results indicated an SMD of 0.345 [−0.029; 0.72] (see Figure S.F. 2.2.1 for forest plot; see Figure S.F. 2.2.2 and Figure S.F. 2.2.3 for the Baujat and funnel plots). This result was heterogeneous, Q = 27.51, *p* < .0001, and, the confidence intervals (CIs) included 0.

Figure S.F. 2.2.1 Severity of neurocognitive impairment assessed with TMT-B: forest plot


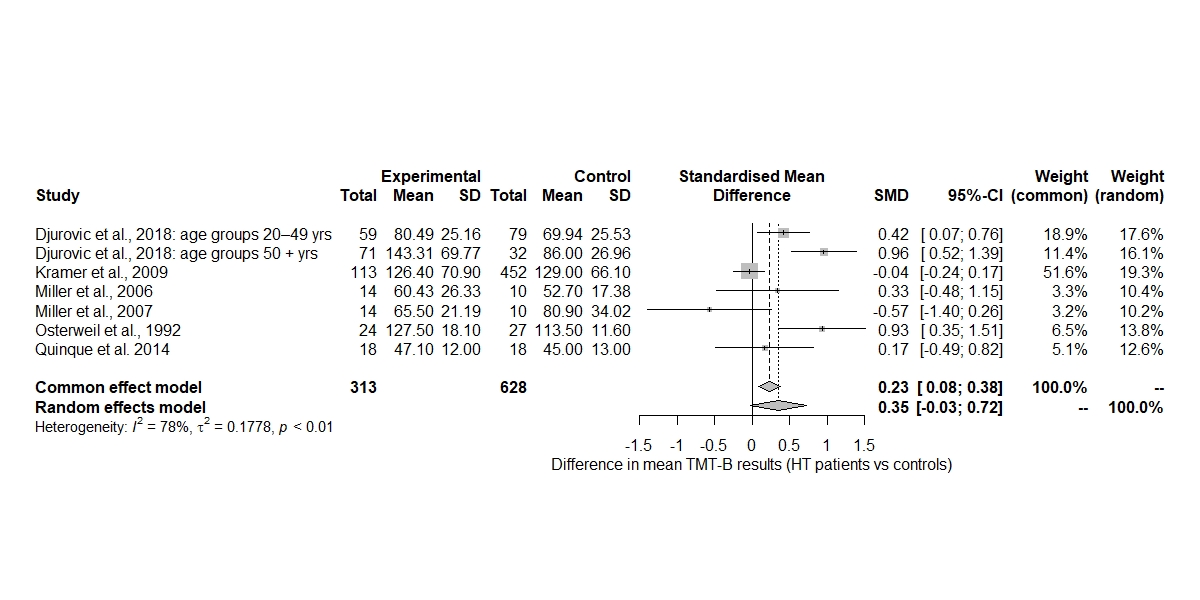


Figure S.F. 2.2.2 TMT B: Baujat plot


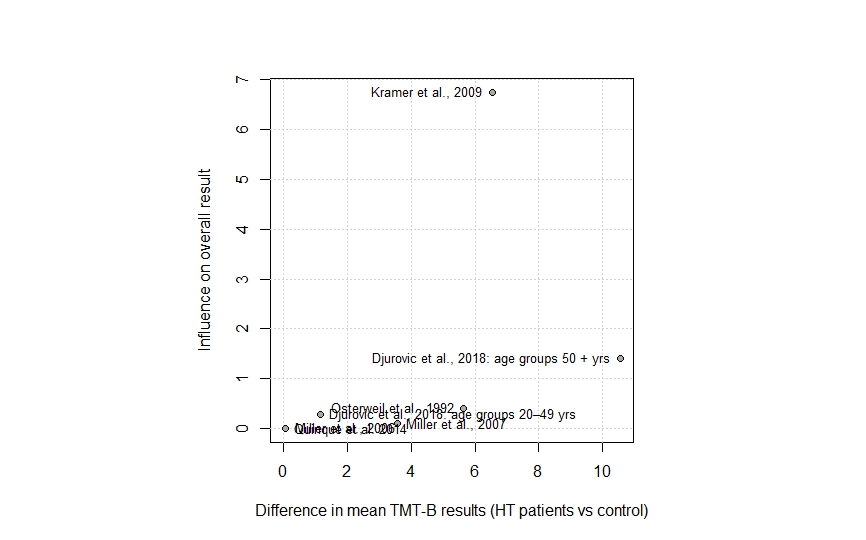


Figure S.F. 2.2.3. TMT B: funnel plot


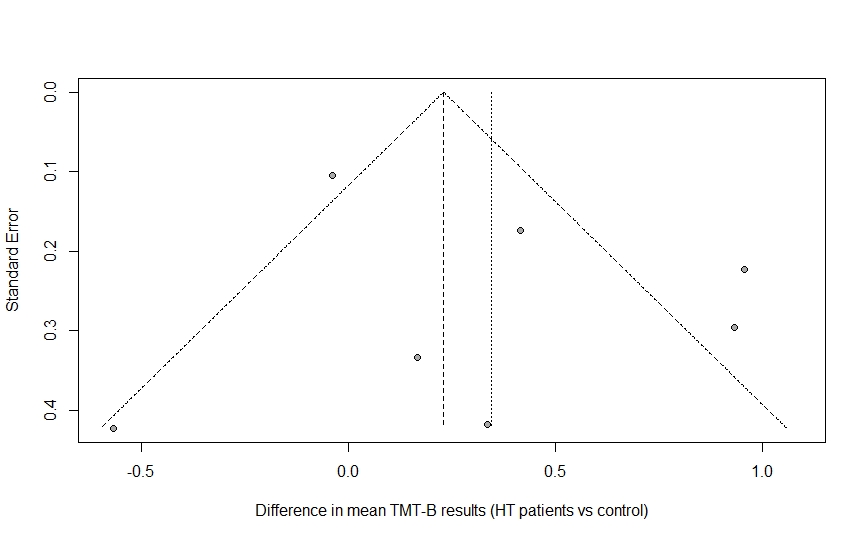


Female ratio (*k* = 5) was a statistically significant moderator, QM (1) = 8.398, *p* = .002 (see Figure S.F. 2.2.4 for the bubble plot). The more women there were in the sample, the lower the difference in reported TMT B completion times. After including this moderator, the TMT B results were homogenous, Q (3) = 3.913, *p* = .27).

Figure S.F. 2.2.4 TMT B and female ratio: bubble plot


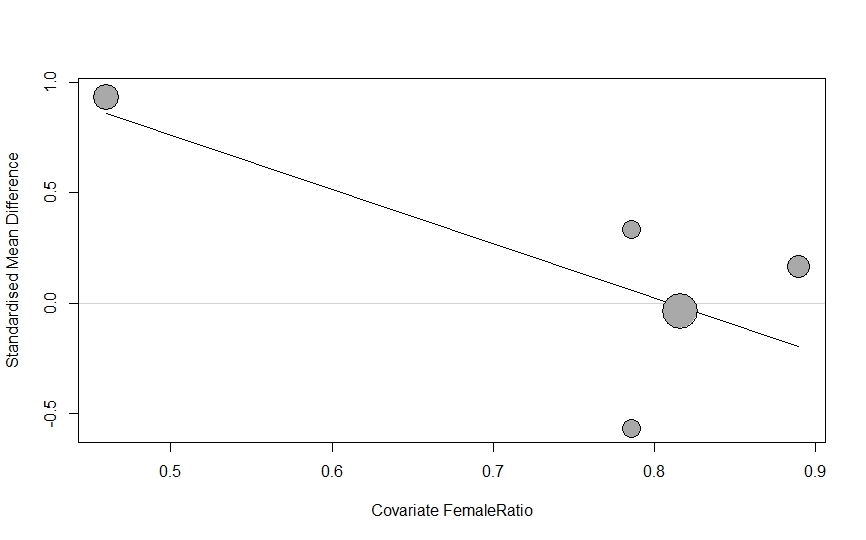


Neither the mean TSH level, QM (1) = 0.594, *p* = .576, quality of included studies, QM (1) = 0.028, *p* = .867, nor mean age, QM (1) = 0.44, *p* = 0.62, were a statistically significant moderators.

*S.F. 2.3. Digit Span Test*

We decided to perform a pooled analysis of the DSTs from both the WMS and the Wechsler Adult Intelligence Scale (WAIS) due to the significant similarities between these tools. For the DST forward results, seven studies were included in the meta-analysis yielded an effect size of −1.33 [−3.096; 0.435] (see Figure S.F. 2.3.1 for forest plot; see Figure S.F. 2.3.2 and Figure S.F. 2.3.3 for the Baujat and funnel plots), which was heterogeneous, Q = 164.97, *p* < .0001).

Figure S.F. 2.3.1 Severity of neurocognitive impairment assessed with DST forwards: forest plot

**
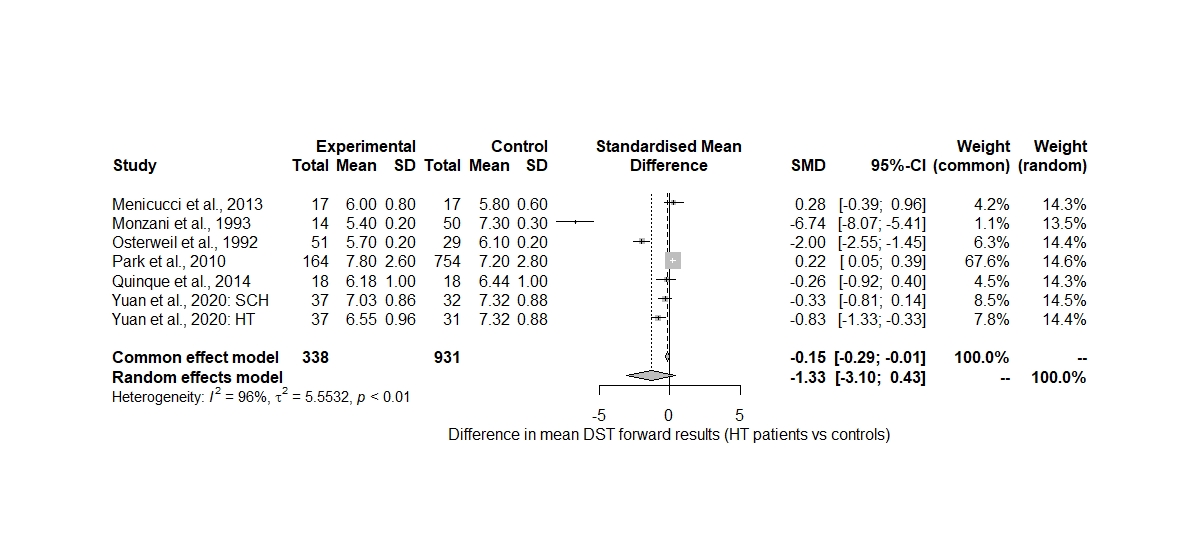
**

Figure S.F. 2.3.2 DST forward: Baujat plot


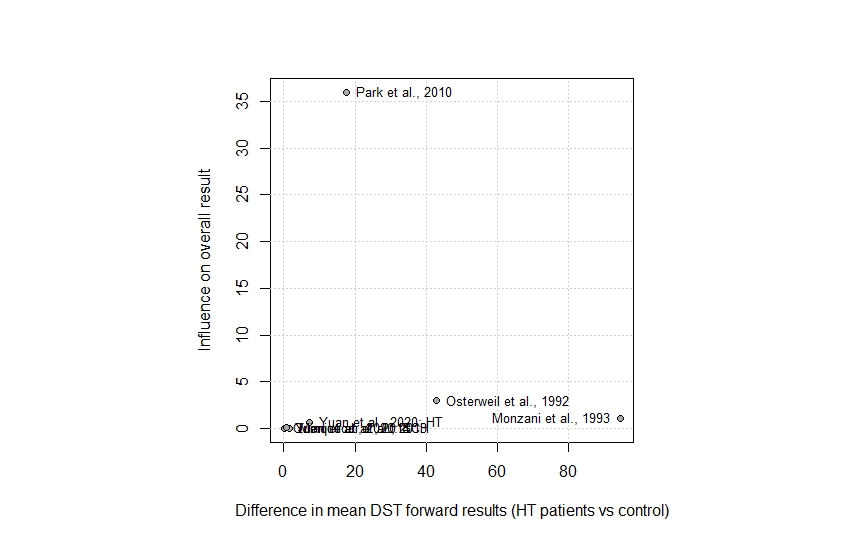


Figure S.F. 2.3.3. DST forward: funnel plot


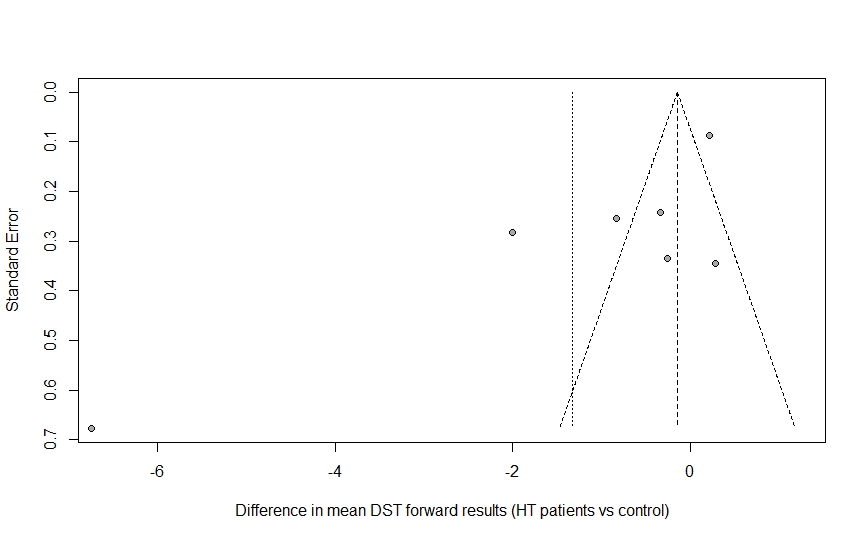


Neither the female ratio, QM (1) = 0.385, *p* = .54, nor mean sample age, QM (1) = 0.003, *p* = .96, were statistically significant moderators. There was insufficient data (*k* = 4) for mean years of education as well as mean fT3 and fT4 to perform calculations. Mean TSH was also not a statistically significant moderator, QM (1) = .0001, *p* = .99.

In a subsequent analysis, the effect sizes of DST backwards (*k* = 9) were calculated. The meta-analysis indicated an effect size of −1.244 [−2.580; 0.092] (see Figure S.F. 2.3.4 for forest plot; see Figure S.F. 2.3.5 and Figure S.F. 2.3.6 for the Baujat and funnel plots). The result was heterogeneous, Q = 233.29, *p* < .0001.

Figure S.F. 2.3.4. Severity of neurocognitive impairment assessed with DST backwards: forest plot


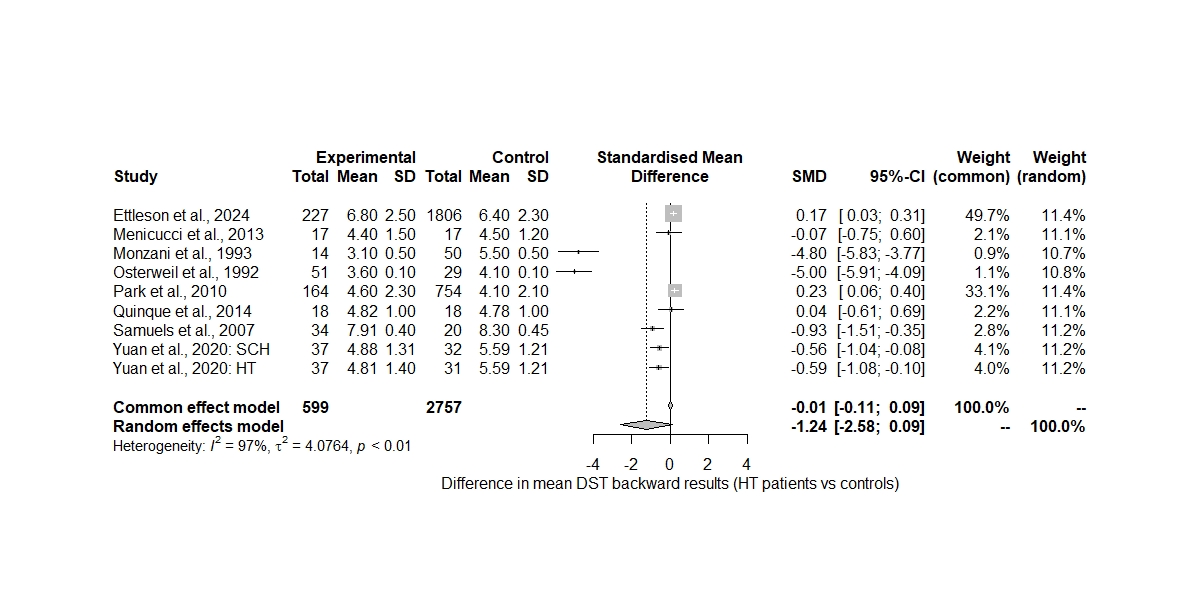


Figure S.F. 2.3.5. DST backward: Baujat plot


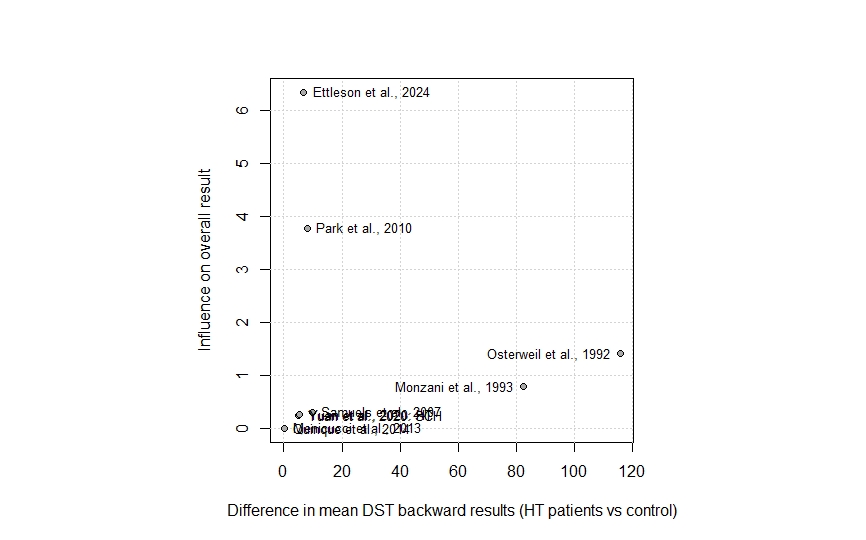


Figure S.F. 2.3.6. DST backward: funnel plot


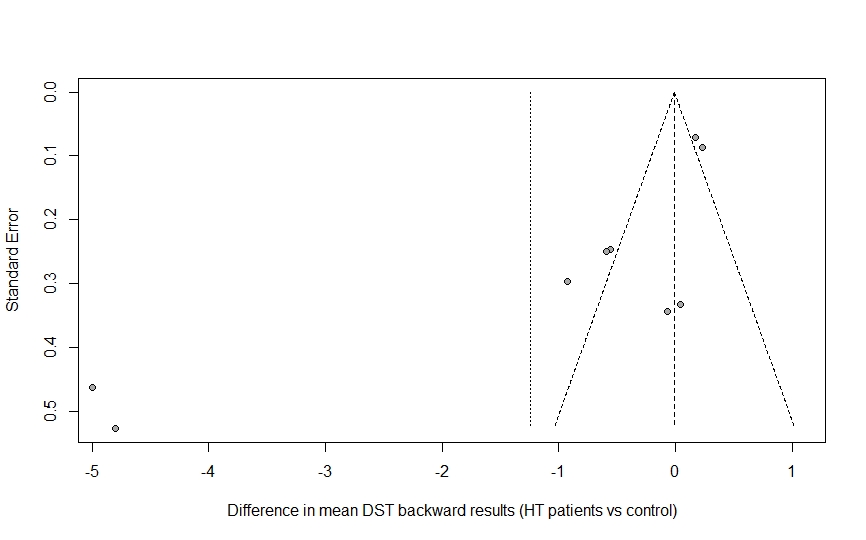


Mean fT4 values ​​(*k* = 5) turned out to be a statistically significant moderator, QM (1) = 6.803, *p* = .009 (see Figure S.F. 2.3.7 for the bubble plot). After taking this variable into account, the heterogeneity of the DST results was still statistically significant, Q(3) = 72.08, *p* < .001).

Figure S.F. 2.3.7. DST backward and mean fT4 levels: bubble plot


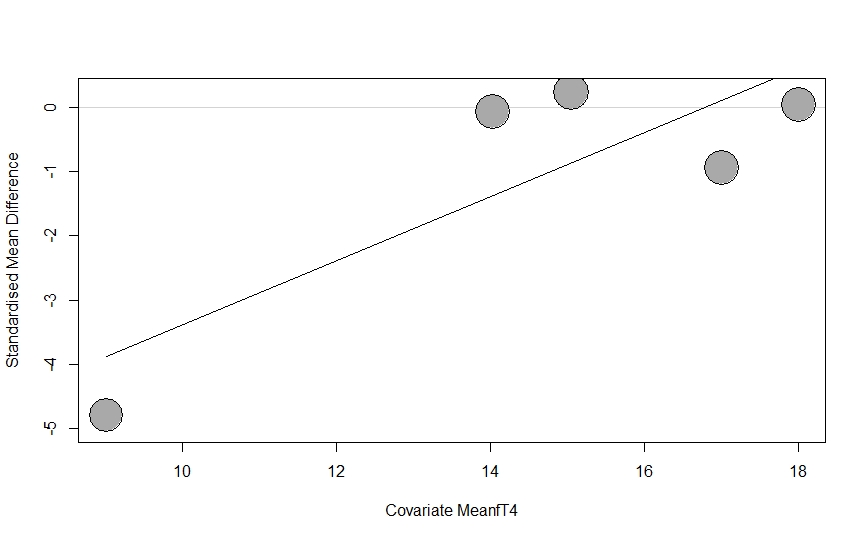


Female ratio, QM (1) = 0.12, *p* = .73, mean sample age, QM (1) = 0.39, *p* = .53, mean years of education, *k* = 5, QM (1) = 0.196, *p* = .66, mean TSH level, ​(QM (1) = 0.663, *p* = .42, quality of studies, QM (1) = 0.001, *p* = .97, were not statistically significant moderators.

*S.F. 2.4. Fluency*

In the case of verbal fluency, eight studies using this tool were identified. In some studies, the SEM or medians and the IQR were used instead of SDs. The six studies that were included in the analysis gave a total of seven comparisons, giving an SMD = −0.448 [−0.935; 0.040] (see Figure S.F. 2.4.1 for forest plot, Figure S.F. 2.4.2 and Figure S.F. 2.4.3 for the Baujat and funnel plots). The result was heterogeneous, Q = 146.41, *p* < 0.0001, and female ratio, *k* = 5, QM (1) = 0.017, *p* = 0.896, mean sample age, QM (1) = 0.892, *p* = .345, mean years of education, *k* = 5, QM (1) = 3.254, *p* = .071, quality of studies included, QM (1) = 0.0, *p* = .999, and mean TSH levels, QM (1) = 0.073, *p* = .787, were not statistically significant moderators.

Figure S.F. 2.4.1 Severity of neurocognitive impairment assessed with verbal fluency: forest plot


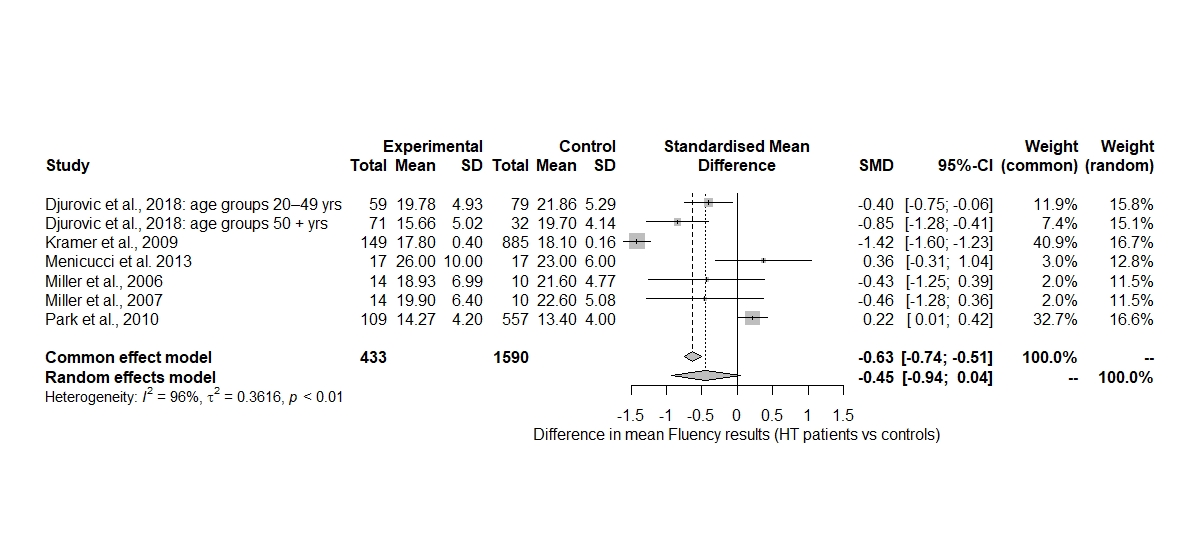


Figure S.F. 2.4.2. Fluency: Baujat plot


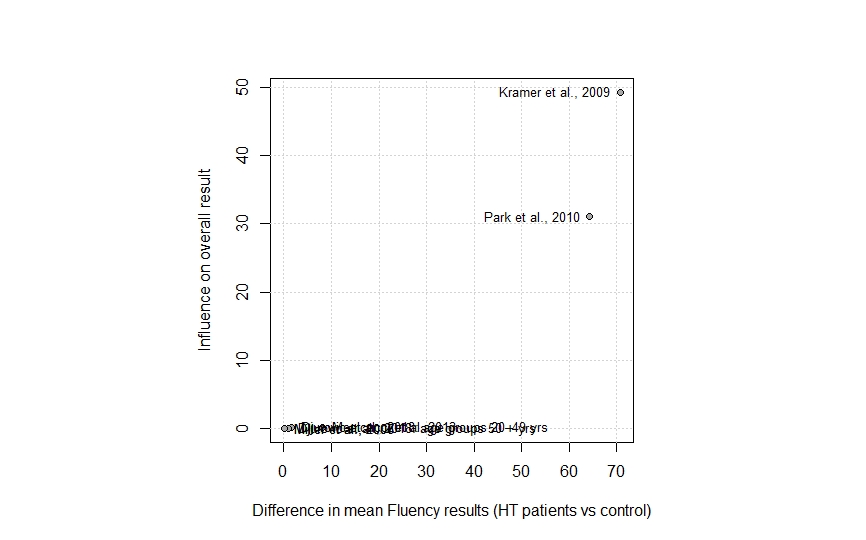


Figure S.F. 2.4.3. Fluency: funnel plot


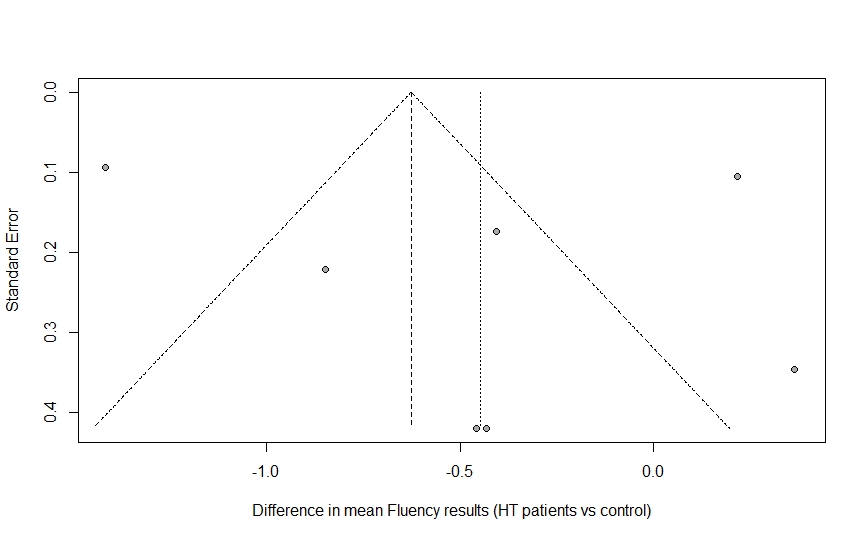


*Supplementary File 2. Meta-regressions results*

*6.1. The Mini-Mental State Examination*

- female ratio, QM (1) = 0.528, *p* = .47,
- mean sample ages (14 comparisons), QM (1) = 0.33, *p* = .57,
- mean years of education, (12 comparisons), QM (1) = 2.314, *p* = .13,
- mean TSH level (mIU/L; 13 comparisons), QM (1) = 0.286, *p* = .59,
- mean fT4 (pmol/L; 5 comparisons), QM (1) = 0.009, *p* = .93.
- mean BMI (9 comparisons), QM (1) = 0.045, *p* = .83
- quality of the study, QM (1) = 0.175, *p* = .68

*6.2. Wechsler Memory Scale*

Memory Quotient:

- female ratio, QM (1) = 1.022, *p* = .31,
- mean sample age, QM (1) = 0.768, *p* = .38,
- mean TSH levels, QM (1) = 0.20, *p* = .66,
- mean fT3 values, QM (1) = 0.367, *p* = .55,
- mean fT4 values, QM (1) = 1.806, *p* = 0.18,
- quality of the study, QM (1) = 0.035, *p* = .85

Mental Control subscale:

- female ratio, QM (1) = 0.313, *p* = .58,
- mean sample age, QM (1) = 0.427, *p* = .51,
- mean TSH levels, QM (1) = 0.012, *p* = .91,
- mean fT3 values, QM (1) = 0.021, *p* = .89,
- mean fT4 values, QM (1) = 1.242, *p* = .27,
- quality of the study, QM (1) = 0.844, *p* = .36

*Supplementary File 3. Qualitative descriptions of the studies presenting data obtained using EEG, EMG, visual and auditory evoked potentials*

EEG results were presented in only one study,^1^ for which data were collected in Italy. In this study, both the resting-state and stimulation were assessed. The results in the resting-state included reduced alpha rhythm in the right hemisphere and bilaterally in the frontal areas. For stimulation, activity in the parietal areas was lateralized toward the right hemisphere in subclinical hypothyroidism (SCH). However, these results should be interpreted with caution due to the lack of replication.

In the case of EMG, one study was conducted in India^2^ and one in Italy^3^. A total of 58 HT patients and 30 controls participated in both studies. The studies included in the review had different data collection methodologies: respectively, the first study assessed blink reflex whereas the second evaluated a number of measures including the cortical silent period, active motor threshold, and resting motor threshold. Also, the data was collected from the right first dorsal interosseus muscle. Both studies showed statistically significant differences between HT patients and healthy/euthyroid controls, including prolonged second ipsilateral response, reduced short interval cortical inhibition, and higher active and resting motor thresholds.

Visual evoked potentials were assessed in three studies.^4-6^ Data were collected in India, Turkey, and the USA, and the study group consisted of a total of 114 HT patients and 90 healthy/euthyroid controls. In two of the three studies,^4,6^ statistically significant differences were observed between HT patients and healthy/euthyroid controls consisting of a higher N135 latency period in the right eye,^4^ and longer latencies on the 20-minute check.^6^ In the study by Naziel et al.,^5^ no statistically significant differences were observed, but the researchers focused exclusively on the P100 peak.

Data on auditory evoked potentials were the subject of 11 studies, which included a total of 488 HT patients and 368 healthy/euthyroid controls. Most studies used the oddball paradigm, which found a statistically significant increase in P300 latency in the HT patients compared to healthy/euthyroid controls. Some studies also indicated differences in P100,^7^ P200 (Anjana et al., 2008),^8^ N100,^7-9^ and N200^8,10^ wave latencies. In the case of amplitudes, statistically significant differences were observed between HT patients and controls, specifically higher P100,^7^ and smaller N100,^7^ and higher P300^10^ in the HT group. Most of the studies identified in this review indicated no statistically significant associations between wave characteristics and laboratory test results. However, in Anjana et al.^8^ and Sharma et al.^11^ observed statistically significant positive associations between P300 and TSH, and negative associations between P200 and P300 and fT4 and T4. For details, see table 4 in main text.

References

1. Menicucci D, Sebastiani L, Comparini A, et al. Minimal changes of thyroid axis activity influence brain functions in young females affected by subclinical hypothyroidism. *Arch Ital Biol*. 2013;151(1):1-10, https://doi.org/10.4449/aib.v151i1.1474

2. Kakked G, Bhatt N, Lakhani J, Prakash S. Electromyographic evaluation of blink reflex as a tool for early diagnosis of neurological dysfunction in patients of hypothyroidism. *Ann Neurosci*. 2013;20(3):95-98, https://doi.org/10.5214/ans.0972.7531.200304

3. Rizzo V, Crupi D, Bagnato S, et al. Neural response to transcranial magnetic stimulation in adult hypothyroidism and effect of replacement treatment. *J Neurol Sci*. 2008;266(1-2):38-43, https://doi.org/10.1016/j.jns.2007.08.031

4. Jaiswal P, Saxena Y, Gupta R, Kaushik RM. Pattern Reversal Visual Evoked Potential and Cognitive Functions in Subclinical Hypothyroid Subjects. *J Neurosci Rural Pract*. 2016;7(Suppl 1):S46-S51, https://doi.org/10.4103/0976-3147.196470

5. Nazliel B, Akbay E, Irkeç C, Yetkin I, Ersoy R, Törüner F. Pattern visual evoked potential (PVEP) evaluation in hypothyroidism. *J Endocrinol Invest*. 2002;25(11):955-958. doi:10.1007/BF03344067

6. Osterweil D, Syndulko K, Cohen SN, et al. Cognitive function in non-demented older adults with hypothyroidism. *J Am Geriatr Soc*. 1992;40(4):325-335, https://doi.org/10.1111/j.1532-5415.1992.tb02130.x

7. Oerbeck B, Reinvang I, Sundet K, Heyerdahl S. Young adults with severe congenital hypothyroidism: cognitive event related potentials (ERPs) and the significance of an early start of thyroxine treatment. *Scand J Psychol*. 2007;48(1):61-67, https://doi.org/10.1111/j.1467-9450.2006.00545.x

8. Anjana Y, Tandon OP, Vaney N, Madhu SV. Cognitive status in hypothyroid female patients: event-related evoked potential study. *Neuroendocrinology*. 2008;88(1):59-66, https://doi.org/10.1159/000117713

9. Ozata M, Ozkardes A, Corakci A, Beyhan Z, Gundogan MA. Event-related brain potentials in patients with hypothyroidism. *Endocr Pract*. 1997;3(6):349-352, https://doi.org/10.4158/EP.3.6.349

10. Waliszewska-Prosół M, Bladowska J, Budrewicz S, Sąsiadek M, Dziadkowiak E, Ejma M. The evaluation of Hashimoto's thyroiditis with event-related potentials and magnetic resonance spectroscopy and its relation to cognitive function. *Sci Rep*. 2021;11(1):2480. Published 2021 Jan 28, https://doi.org/10.1038/s41598-021-82281-6

11. Sharma K, Behera JK, Sood S, Rajput R, Satpal, Praveen P. Study of cognitive functions in newly diagnosed cases of subclinical and clinical hypothyroidism. *J Nat Sci Biol Med*. 2014;5(1):63-66, https://doi.org/10.4103/0976-9668.127290

*Supplementary File 4. Electromyography, evoked potentials and electroencephalography: meta-analyses*

N100 latencies were reported in two studies,^1,2^ with Anjana et al.^1^ reporting means and SDs for Fz, Cz, and Pz electrodes. Unfortunately, even when combining results from consecutive electrodes, there were insufficient data to conduct a meta-analysis. N200 latencies were reported in three studies,^1-3^ with Anjana et al.,^1^ and Waliszewska-Prosół, et al.^3^ reporting data for consecutive Fz, Cz, and Pz electrodes, and Ozata et al.^2^ reporting an average value. Nevertheless, we decided to perform a pooled analysis of all comparisons. However, it should be strongly emphasized that the results of this meta-analysis, presented in Figure S.F. 8.1, should be interpreted with great caution.

Figure S.F. 8.1. Differences in N200 latencies: forest plot


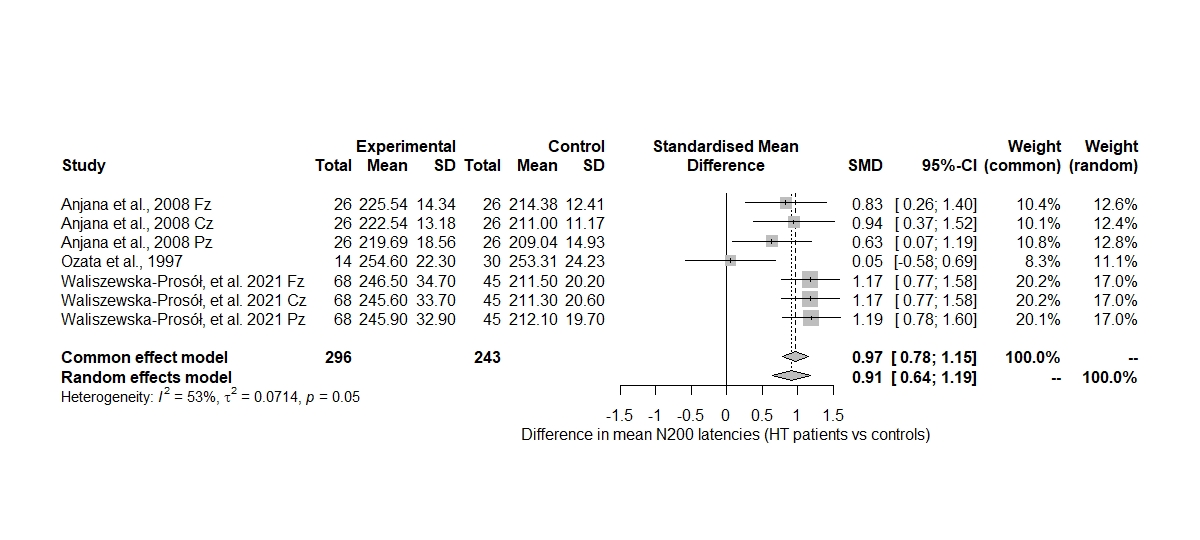


The results indicated a statistically significant difference between HT patients and controls in the N200 latency with the size SMD = 0.912 [0.638; 1.186]. Funnel and Baujat plots are presented below.

Figure S.F. 8.2. N200 latency: Baujat plot


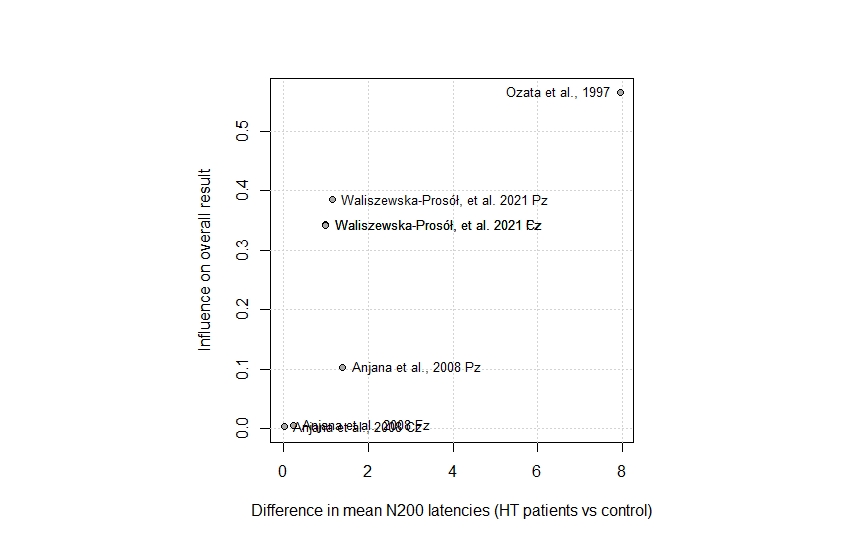


Figure S.F. 8.3. N200 latency: Funnel plot


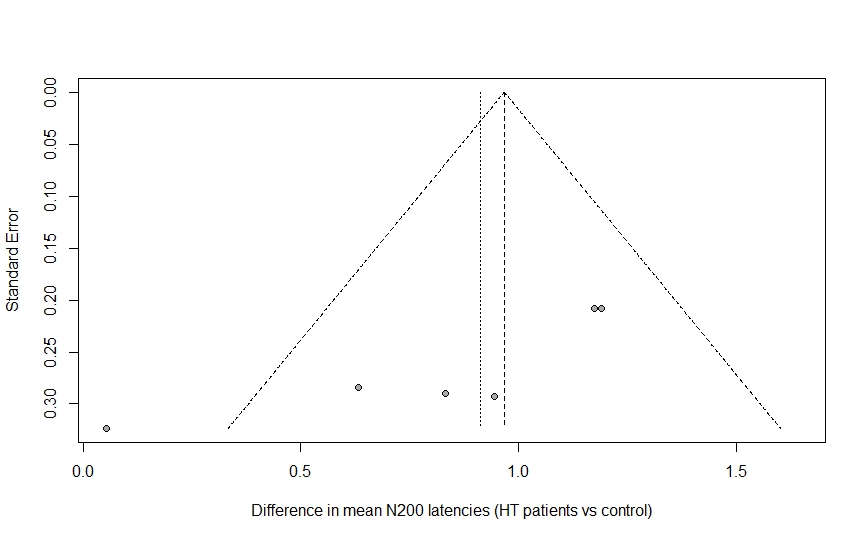


Statistically significant heterogeneity was observed, Q = 12.68; p = 0.048. A meta-regression showed that the female ratio was not a statistically significant moderator, QM (1) = 1.081, p = .30. The mean age of the participants turned out to be a statistically significant moderator, QM (1) = 9.947, p = .002, and after taking it into account, the results were homogeneous, Q(5) = 2.736, p = .74. They indicated that the higher the mean age, the higher the SMD (see bubble plot in Figure S.F. 8.4).

Figure S.F. 8.4. N200 latency and mean age: bubble plot


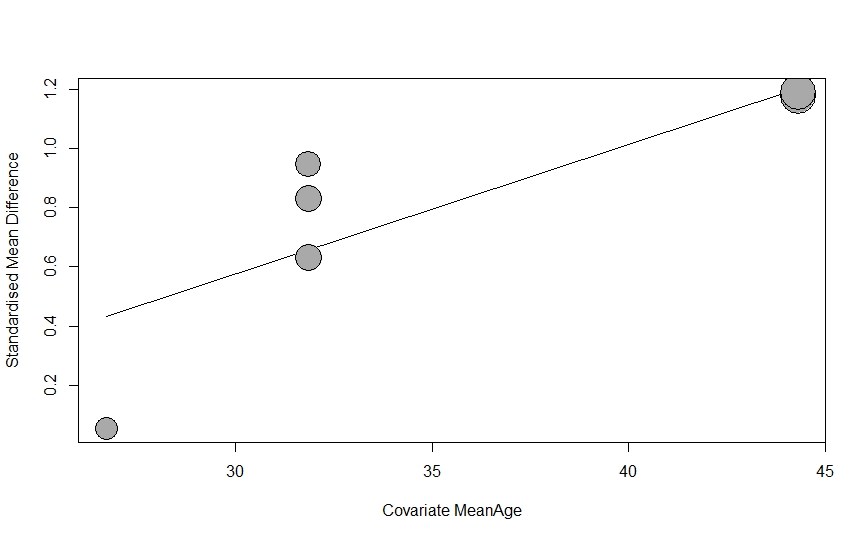


The next statistically significant moderator was mean TSH level, QM (1) = 11.692, p < .001. After taking into account the TSH level, the results were homogeneous, Q(5) = 0.991 p = .96. The analysis showed that the higher the TSH level, the lower the SMD (see bubble plot in Figure S.F. 8.5).

Figure S.F. 8.5. N200 latency and mean TSH levels: bubble plot


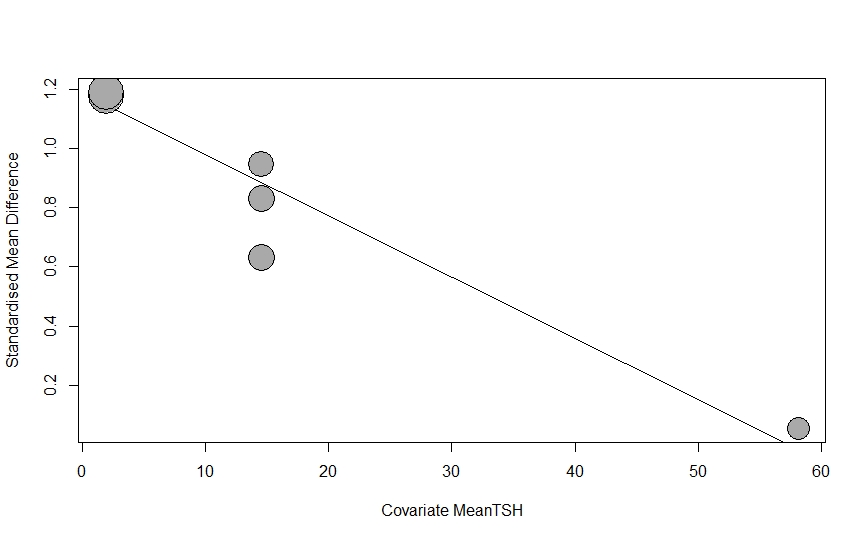


Mean fT4 level also turned out to be a statistically significant moderator, QM (1) = 9.773, p = .002, and after taking it into account, the results were homogeneous, Q(5) = 2.909, p = .71. The higher the fT4 level, the higher the SMD (see bubble plot in Figure S.F. 8.6.).

Figure S.F. 8.6. N200 latency and mean fT4 levels: bubble plot


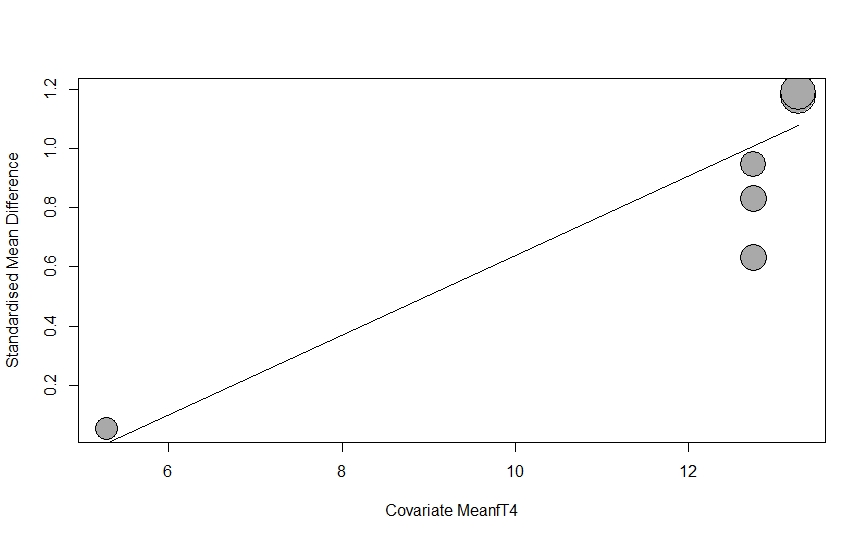


Another statistically significant moderator was mean fT3 level, QM (1) = 11.411, p < .001. After taking it into account, the results were homogeneous, Q(5) = 1.271 p = .94. With increasing fT3, SMD also increased (see bubble plot in Figure S.F. 8.7).

Figure S.F. 8.7. N200 latency and mean fT3 levels: bubble plot


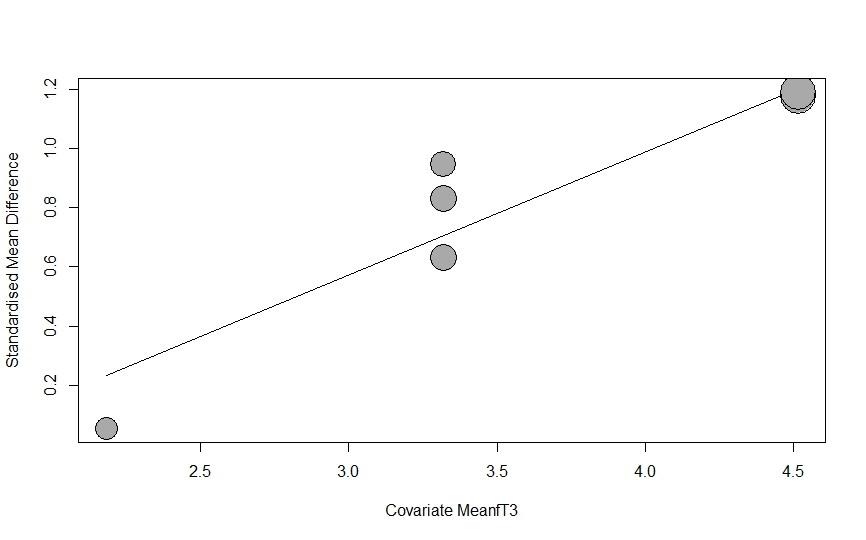


However, it should be noted that these comparisons only come from three studies. Therefore additional sensitivity analyses were performed in the form of jackknife analyses, and extreme caution should be exercised when generalizing the obtained results.

P200 latency was reported in only two studies,^1,2^ one of which reported means and SDs for three electrodes. However, this number (k = 4) was too small to allow for further pooled analyses.

Further inspection of the included studies indicated that N100 and P200 amplitude was reported in one study,^1^ N200 in two studies,^1,3^ with data available for three electrodes in each study. However, given that this would be a comparison of only two studies, we decided not to perform pooled calculations. P300 amplitude was reported in a total of four studies,^1-4^ with Anjana et al.,^1^ Ozata et al.,^2^ and Waliszewska-Prosół et al.^3^ reporting data from three electrodes, while Sharma et al.^4^ reported data for both HT and SCH. For P300 amplitude, we decided to perform a meta-analysis of a total of 11 comparisons (see Figure S.F. 8.8).

Figure S.F. 8.8. Differences in P300 amplitudes: forest plot


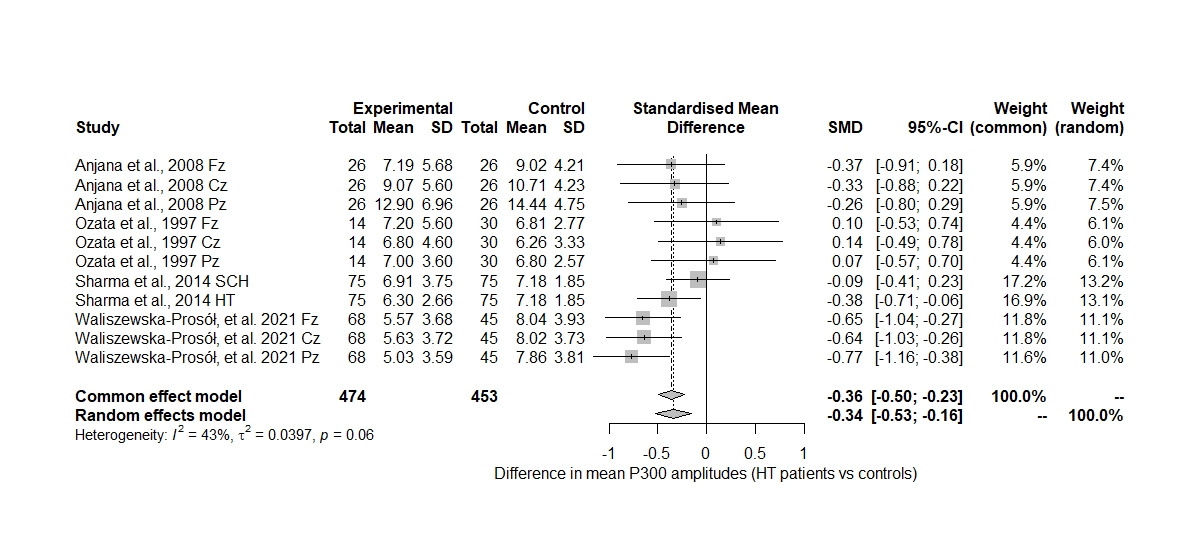


The results of the analyses showed a statistically significant difference between groups with an effect size of SMD = -0.343 [-0.527; -0.160] (see Figures S.F. 8.9 – 8.10 for funnel and Baujat plots). The results were homogeneous, Q = 17.54, p = .06, and therefore, potential moderators were not assessed.

Figure S.F. 8.9. P300 amplitude: Baujat plot


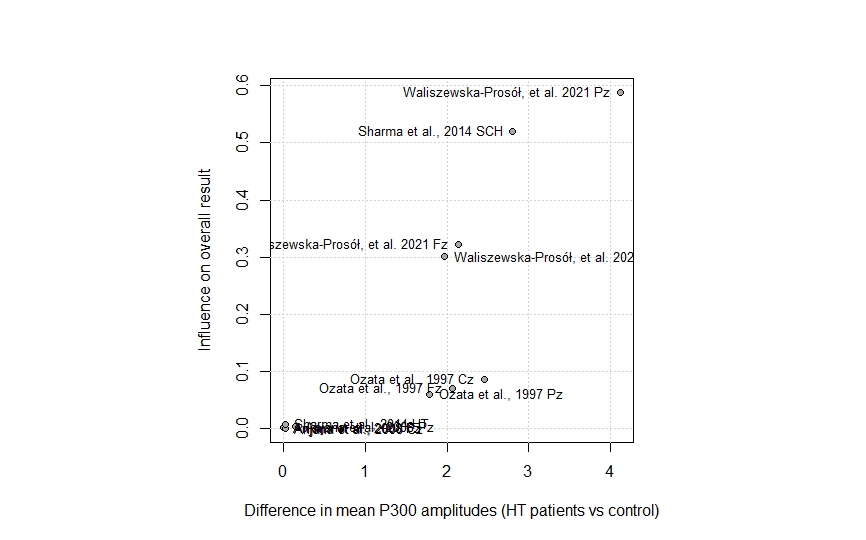


Figure S.F. 8.10. P300 amplitude: Funnel plot


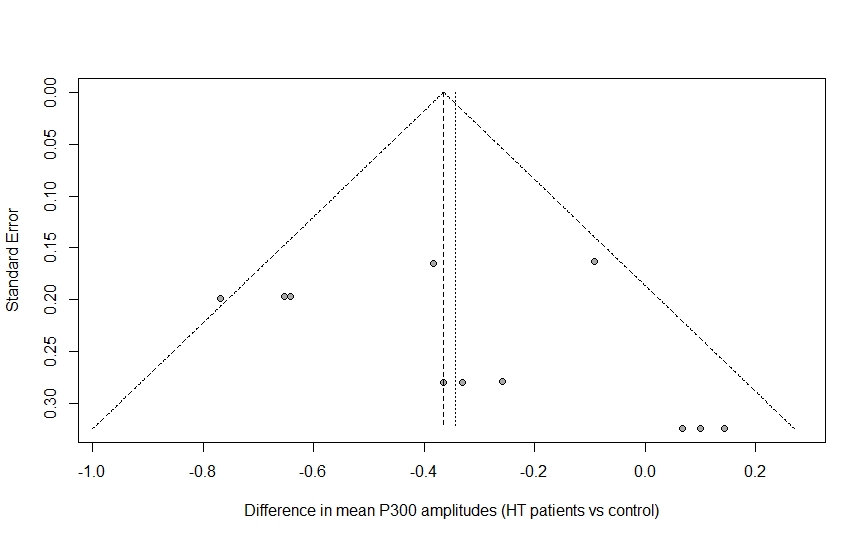


References

1. Anjana Y, Tandon OP, Vaney N, Madhu SV. Cognitive status in hypothyroid female patients: event-related evoked potential study. *Neuroendocrinology*. 2008;88(1):59-66, https://doi.org/10.1159/000117713

2. Ozata M, Ozkardes A, Corakci A, Beyhan Z, Gundogan MA. Event-related brain potentials in patients with hypothyroidism. *Endocr Pract*. 1997;3(6):349-352, https://doi.org/10.4158/EP.3.6.349

3. Waliszewska-Prosół M, Bladowska J, Budrewicz S, Sąsiadek M, Dziadkowiak E, Ejma M. The evaluation of Hashimoto's thyroiditis with event-related potentials and magnetic resonance spectroscopy and its relation to cognitive function. *Sci Rep*. 2021;11(1):2480. Published 2021 Jan 28, https://doi.org/10.1038/s41598-021-82281-6

4. Sharma K, Behera JK, Sood S, Rajput R, Satpal, Praveen P. Study of cognitive functions in newly diagnosed cases of subclinical and clinical hypothyroidism. *J Nat Sci Biol Med*. 2014;5(1):63-66, https://doi.org/10.4103/0976-9668.127290

*Supplementary Figure 1. Prevalence of cognitive impairment in hypothyroid patients: funnel plot*


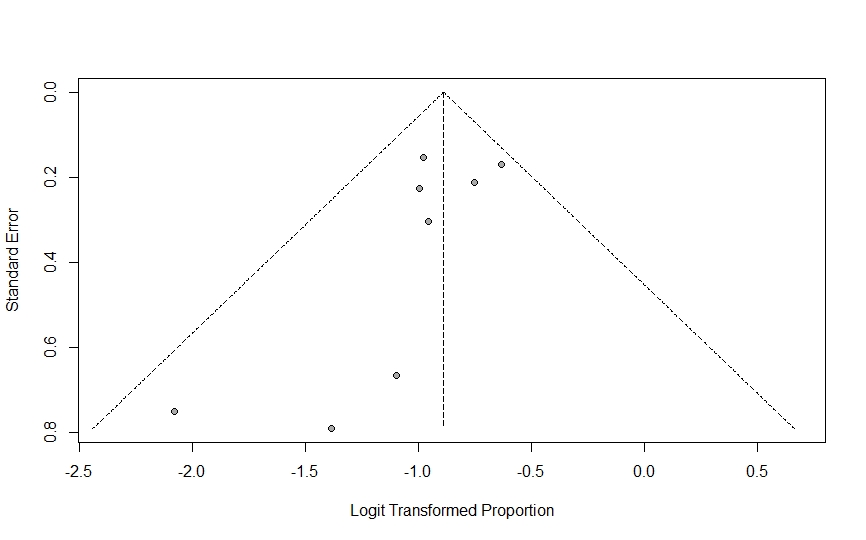


*Supplementary Figure 2. Prevalence of cognitive impairment in hypothyroid patients: baujat plot*


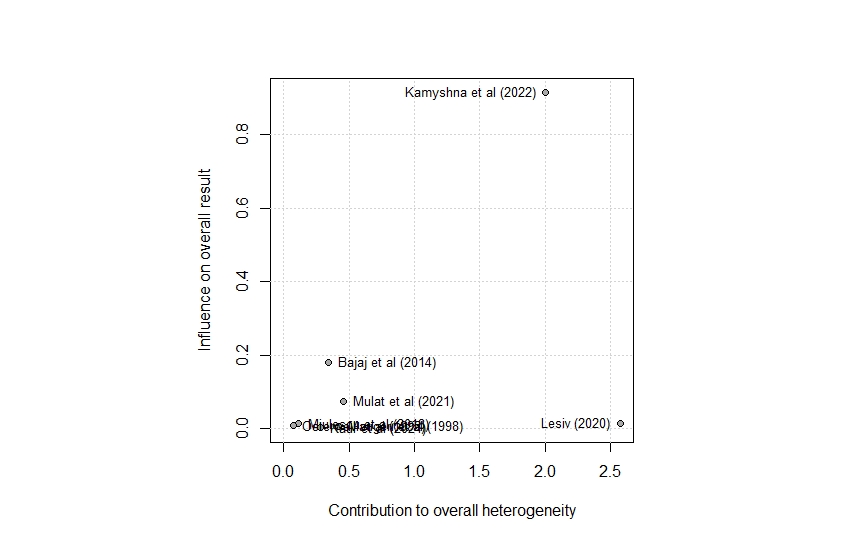


*Supplementary Figure 3. Correlations between TSH and MMSE results: Funnel plot*


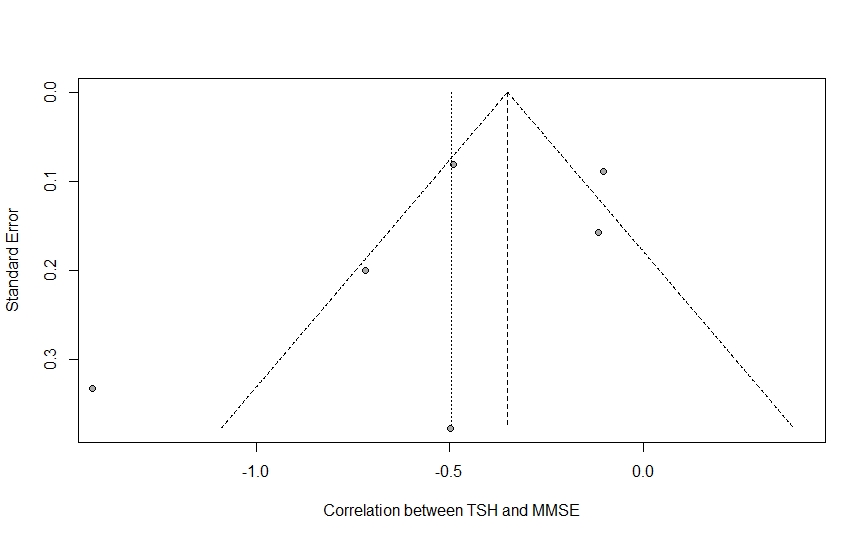


*Supplementary Figure 4. Correlations between TSH and MMSE results: Baujat plot*


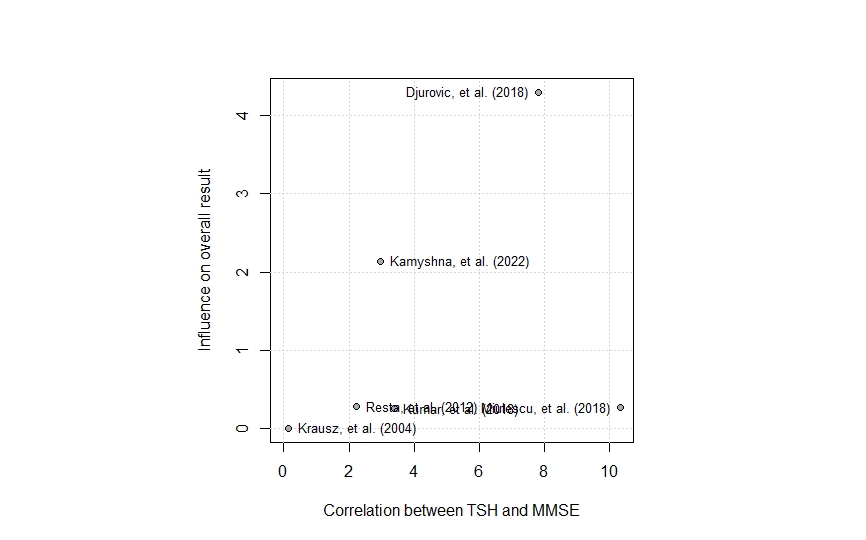


*Supplementary Figure 5. Severity of neurocognitive impairment assessed with MMSE: Baujat plot*


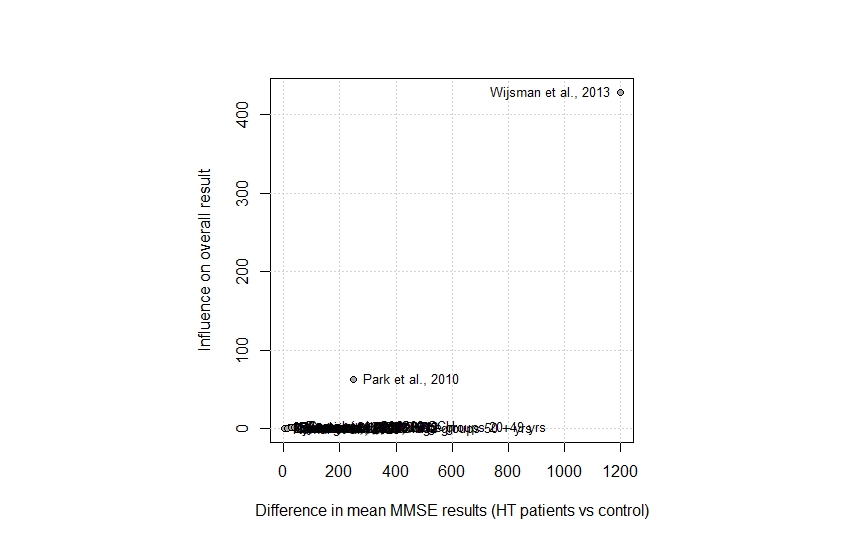


*Supplementary Figure 6. Severity of neurocognitive impairment assessed with MMSE: Funnel plot*


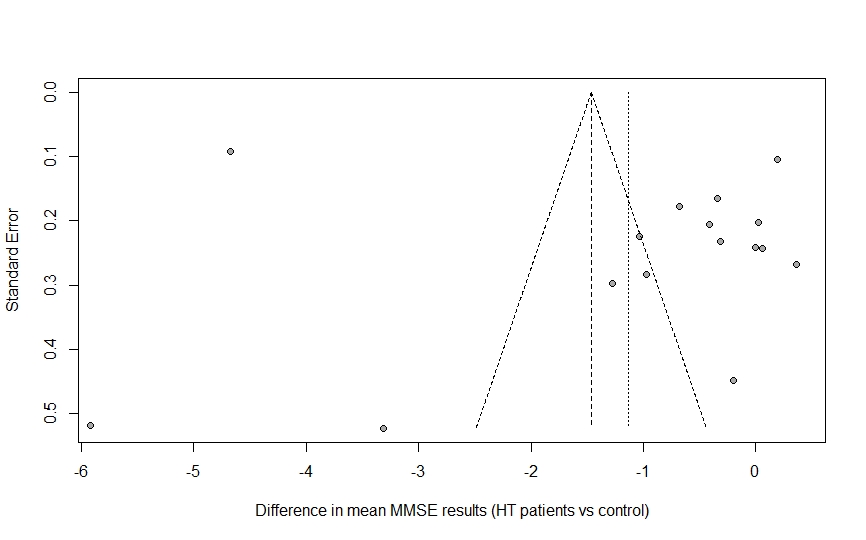


*Supplementary Figure 7. MMSE and mean fT3: Bubble plot*


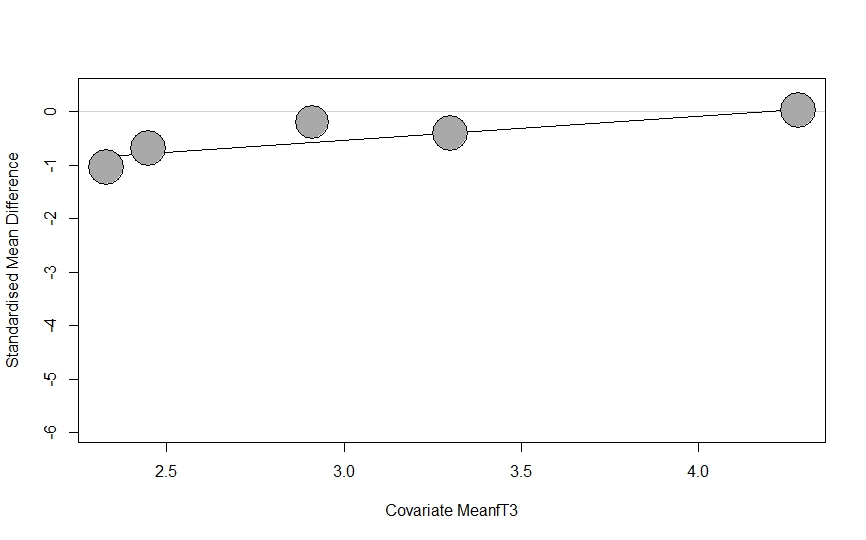


*Supplementary Figure 8. WMS Memory Quotient: Baujat plot*


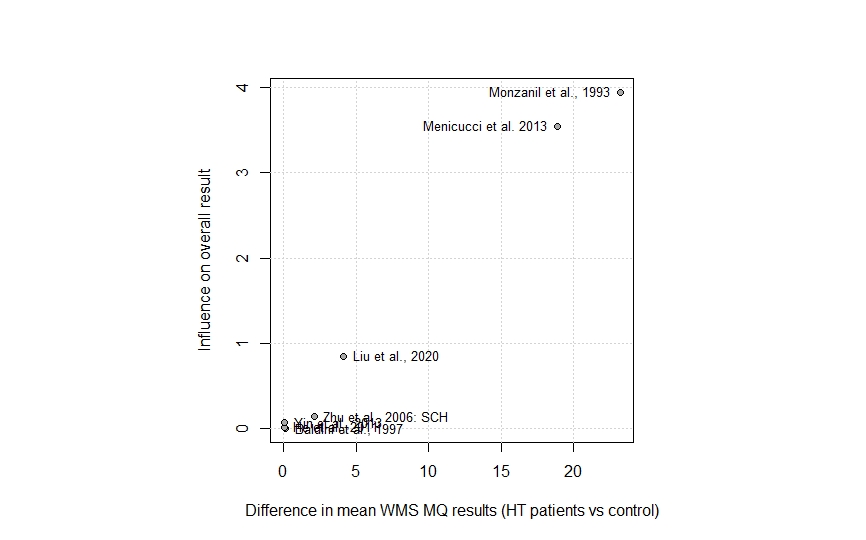


*Supplementary Figure 9. WMS Memory Quotient: funnel plot*


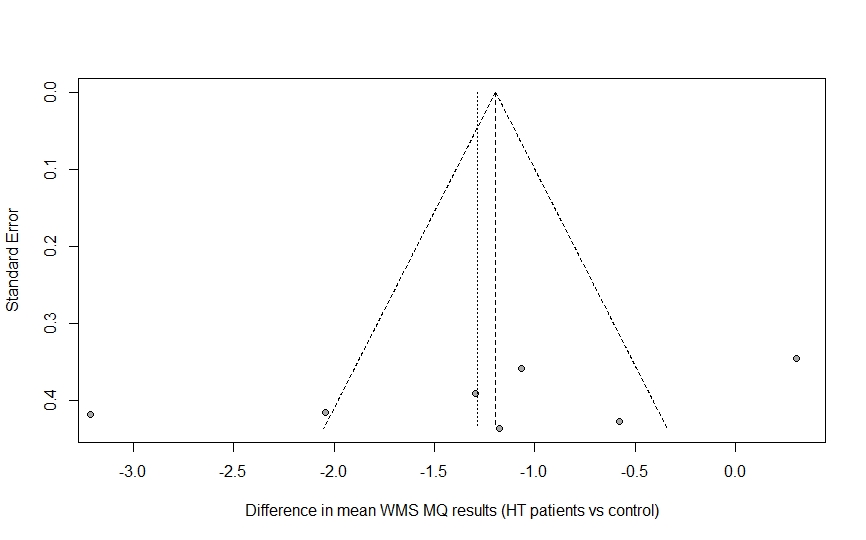


*Supplementary Figure 10. Severity of neurocognitive impairment assessed with WMS Mental Control subscale: forest plot*


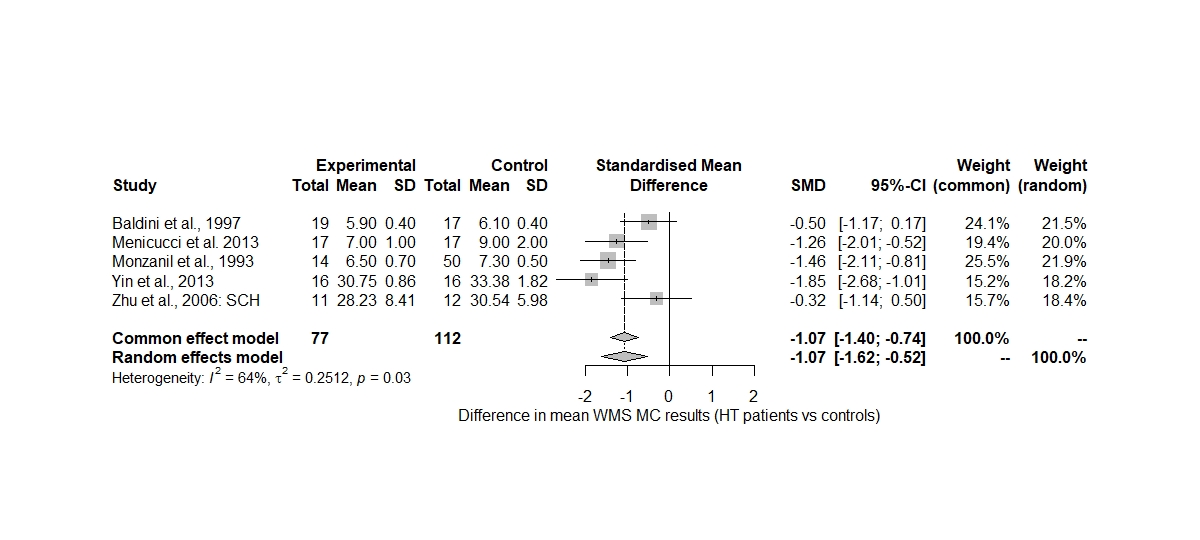


*Supplementary Figure 11. WMS Mental Control: Baujat plot*


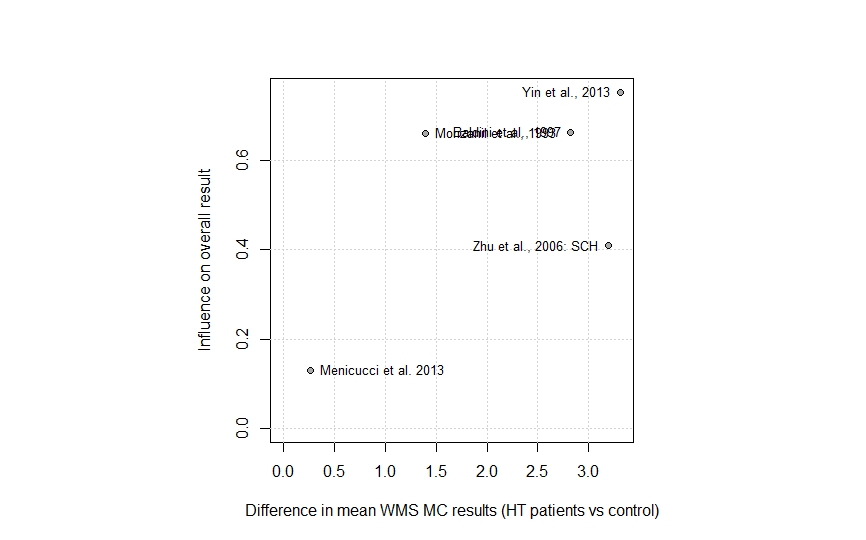


*Supplementary Figure 12. WMS Mental Control: funnel plot*


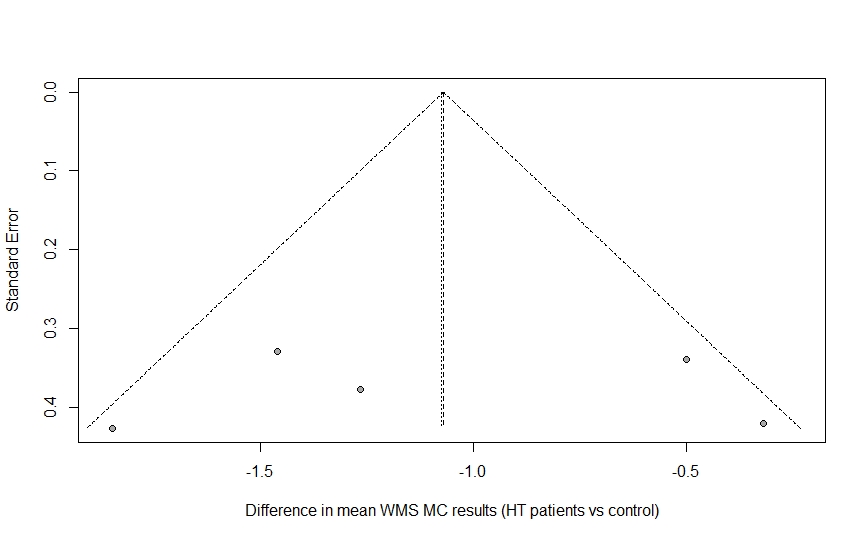


*Supplementary Figure 13. P300 latency: Baujat plot*


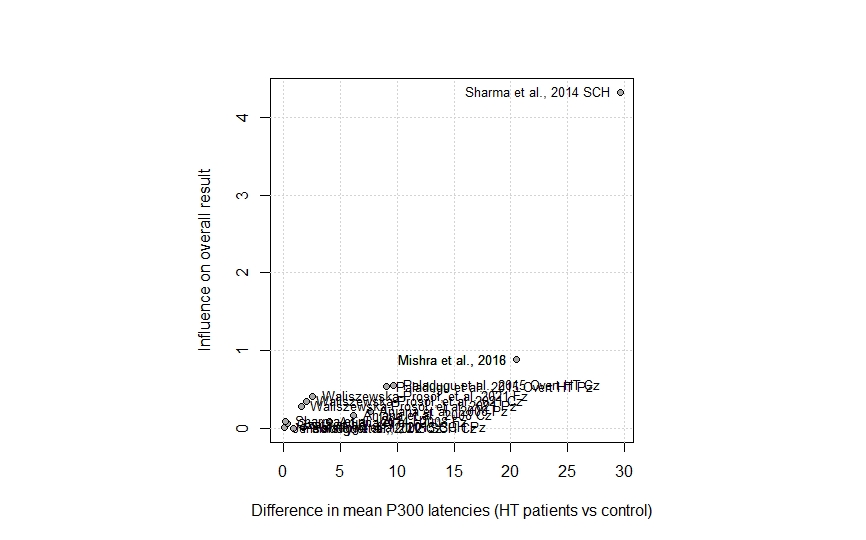


*Supplementary Figure 14. P300 latency: Funnel plot*


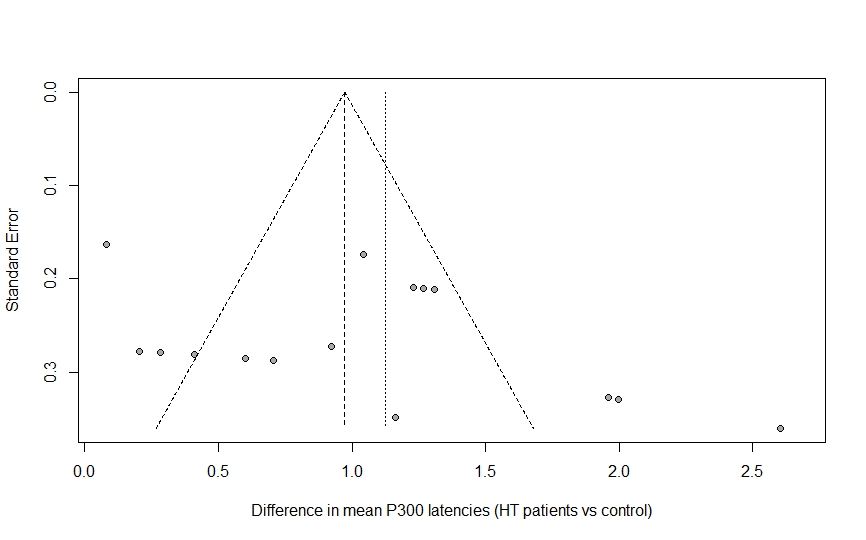

Supplement: Supplementary file 11 — Supporting Information [file ALZ-21-e70924-s011.docx]
